# Genomic and transcriptomic characterization of carbohydrate-active enzymes in the anaerobic fungus *Neocallimastix cameroonii* var. *constans*

**DOI:** 10.1093/g3journal/jkaf137

**Published:** 2025-06-16

**Authors:** Elaina M Blair, Tejas A Navaratna, Colleen B Ahern, Ramya Ragunathan, Jennifer L Brown, Stephen J Mondo, Anna Lipzen, Radwa A Hanafy, Kurt LaButti, Jayson Talag, Kerrie Barry, Mansi Chovatia, Mei Wang, Jessy Gonzalez, Xuefeng Peng, Igor V Grigoriev, Michelle A O’Malley

**Affiliations:** Department of Chemical Engineering, University of California, Santa Barbara, Santa Barbara, CA 93106, USA; Department of Chemical Engineering, University of California, Santa Barbara, Santa Barbara, CA 93106, USA; Department of Chemical Engineering, University of California, Santa Barbara, Santa Barbara, CA 93106, USA; Department of Chemical Engineering, University of California, Santa Barbara, Santa Barbara, CA 93106, USA; Department of Chemical Engineering, University of California, Santa Barbara, Santa Barbara, CA 93106, USA; U.S. Department of Energy Joint Genome Institute, Lawrence Berkeley National Laboratory, Berkeley, CA 94720, USA; U.S. Department of Energy Joint Genome Institute, Lawrence Berkeley National Laboratory, Berkeley, CA 94720, USA; Department of Chemical and Biomolecular Engineering, University of Delaware, Newark, DE 19716, USA; U.S. Department of Energy Joint Genome Institute, Lawrence Berkeley National Laboratory, Berkeley, CA 94720, USA; Arizona Genomics Institute, School of Plant Sciences, University of Arizona, Tucson, AZ 85721, USA; U.S. Department of Energy Joint Genome Institute, Lawrence Berkeley National Laboratory, Berkeley, CA 94720, USA; U.S. Department of Energy Joint Genome Institute, Lawrence Berkeley National Laboratory, Berkeley, CA 94720, USA; U.S. Department of Energy Joint Genome Institute, Lawrence Berkeley National Laboratory, Berkeley, CA 94720, USA; Department of Chemical Engineering, University of California, Santa Barbara, Santa Barbara, CA 93106, USA; Department of Chemical Engineering, University of California, Santa Barbara, Santa Barbara, CA 93106, USA; U.S. Department of Energy Joint Genome Institute, Lawrence Berkeley National Laboratory, Berkeley, CA 94720, USA; Department of Plant and Microbial Biology, University of California, Berkeley, Berkeley, CA 94720, USA; Department of Chemical Engineering, University of California, Santa Barbara, Santa Barbara, CA 93106, USA; Joint BioEnergy Institute, Emeryville, CA 94608, USA; Department of Bioengineering, University of California, Santa Barbara, Santa Barbara, CA 93106, USA

**Keywords:** anaerobic fungi, lignocellulose, RNA-seq, CAZymes, enzyme, genome assembly

## Abstract

Anaerobic gut fungi effectively degrade lignocellulose in the guts of large herbivores, but there remain a limited number of isolated, publicly available, and sequenced strains that impede our understanding of the role of anaerobic fungi within microbial communities. We isolated and characterized a new fungal isolate, *Neocallimastix cameroonii* var. *constans*, providing a transcriptomic and genomic understanding of its ability to degrade diverse carbohydrates. This anaerobic fungal strain was stably cultivated for multiple years in vitro among members of an initial enrichment microbial community derived from goat feces, and it demonstrated the ability to pair with other microbial members, namely, archaeal methanogens to produce methane from lignocellulose. Genomic analysis revealed a higher number of predicted carbohydrate-active enzymes encoded in the *N. cameroonii* var. *constans* genome compared to most other sequenced anaerobic fungi. The carbohydrate-active enzyme profile for this isolate contained 660 glycoside hydrolases, 160 carbohydrate esterases, 194 glycosyltransferases, and 85 polysaccharide lyases. Differential gene expression analysis showed the upregulation of thousands of genes (including predicted carbohydrate-active enzymes) when *N. cameroonii* var. *constans* was grown on lignocellulose (reed canary grass) compared to less complex substrates, such as cellulose (filter paper), cellobiose, and glucose. AlphaFold was used to predict functions of transcriptionally active yet poorly annotated genes, revealing feruloyl esterases that likely play an important role in lignocellulose degradation by anaerobic fungi. The combination of this strain's genomic and transcriptomic characterization, omics-informed structural prediction, and robustness in microbial co-culture make it a well-suited platform to conduct future investigations into bioprocessing and enzyme discovery.

## Introduction

Anaerobic gut fungi are robust lignocellulose degraders, with extensive enzymatic machinery involved in breaking down plant biomass (Hooker *et al*. 2019). They are pivotal members of large herbivore gut microbiomes where they interact with members of a diverse microbial community, including methanogens that catabolize fungal fermentation products (Li *et al*. 2021). Several anaerobic gut fungal genera have been cultivated, and the number of isolated strains continues to increase (Hanafy *et al*. 2022). Still, the genomes of anaerobic gut fungi encode many proteins of yet unknown function (Gruninger *et al*. 2018; Swift *et al*. 2021a), which may bestow these organisms with additional enzymatic mechanisms than those that can be predicted from current annotation tools alone (Lankiewicz *et al*. 2022).

Complicating the sequencing-based analysis of anaerobic gut fungi is their extremely high adenine-thymine (AT) content and many repeat regions in their genomes (Brownlee 1989; Wilken *et al*. 2021), which together make it challenging to assemble high-quality genomes (Edwards *et al*. 2017). However, long-read sequencing has vastly advanced the potential for obtaining high-quality genomes for these microbes (Youssef *et al*. 2013; Wilken *et al*. 2019), enabling the annotation of genes and even the construction of genome-scale metabolic models (Wilken *et al*. 2021). Transcriptomic analyses have improved fungal genome annotations and have identified a trove of potential genes involved in lignocellulose breakdown. For example, exploiting catabolite repression patterns across a range of anaerobic fungal strains has revealed enzymes associated with fungal cellulosomes (Solomon *et al*. 2016; Henske *et al*. 2017), sugar and carbohydrate transporters (Seppälä *et al*. 2019), and stress response genes associated with metabolic reprogramming (Swift *et al*. 2021b). This has accelerated efforts to characterize gut fungal enzymes and sugar transporters via heterologous expression (Perli *et al*. 2021; Podolsky *et al*. 2021; Liu *et al*. 2022; Lillington *et al*. 2023) to link sequence to function. Moreover, while very few anaerobic fungal proteins have high-resolution structures (Dementiev *et al*. 2023), new approaches in cryoEM (Bai *et al*. 2015) and protein structure prediction via AlphaFold (Jumper *et al*. 2021) are accelerating the pace of gene characterization from unconventional microbes. Comparative -omics studies across multiple anaerobic fungal isolates are necessary to identify groups of genes that are uniquely conserved within this clade, which provide a critical starting point to unmask gene function. However, only 7 strains of anaerobic fungi have published genomes with accompanied transcriptomics characterization (Hanafy *et al*. 2023), which severely hampers efforts aimed at linking gene sequence to function.

Anaerobic gut fungi are useful members of the herbivore gut microbiome, particularly for biomass degradation, and other microbial members within the community participate in cross-feeding roles. For example, methanogenic archaea, which play a key role in industrial biogas production (Lyu *et al*. 2018), use hydrogen made by anaerobic fungi and bacteria to generate methane in the herbivore gut (Tapio *et al*. 2017).

This study reports the isolation and high-quality genome and transcriptome of a new fungal isolate, *Neocallimastix cameroonii* var. *constans*, and its synthetic pairing with methanogens. This fungal strain was isolated from a laboratory-cultivated anaerobic microbial community derived from goat feces and cultured for multiple years on lignocellulose, with the initial in vitro growth and first 10 transfers on alfalfa (Peng *et al*. 2021 ). Genomic sequencing, carbohydrate-active enzyme (CAZyme) profiling, phylogenetic analysis, transcriptomic sequencing (RNA-seq), and structure prediction of under-characterized proteins of *N. cameroonii* var. *constans* were performed on cultures grown on a range of different substrates. These results show that this strain has high CAZyme gene content and is predicted to encode a larger number of glycoside hydrolases compared to most other anaerobic gut fungi sequenced to date. Based on structural predictions, there are additional predicted CAZymes (including feruloyl esterases) encoded in this fungal isolate genome that are missed with more traditional annotation methods. Several feruloyl esterases are predicted in proteins containing dockerin domains, thus supporting the importance of cellulosomes in anaerobic fungal carbohydrate degradation. It is also shown that co-cultures containing *N. cameroonii* var. *constans* and methanogenic archaea can produce methane, similar to other reported strains of anaerobic gut fungi (Cheng *et al*. 2009; Swift *et al*. 2019; Leggieri *et al*. 2021).

## Materials and methods

### Microbial enrichment and roll tube isolation of anaerobic fungi

The anaerobic fungus *N. cameroonii* var. *constans* was isolated from a consortium of fungi, methanogens, and bacteria. The consortium was enriched from feces of a San Clemente Island goat at the Santa Barbara Zoo through extended cultivation on an alfalfa substrate and regular antibiotic treatment with penicillin and streptomycin as described previously (Peng *et al*. 2021). Shortly after the 10-passage consecutive batch culture enrichment (Peng *et al*. 2021), the substrate was changed to reed canary grass, and the community was cultivated for approximately 3 years via regular anaerobic passage in Hungate tubes (9 mL MC– media (Peng *et al*. 2018) with approximately 0.1 g dried, milled reed canary grass) with a transfer every 3–4 days. Cultures were passaged using a sterile syringe-needle anaerobic technique, with 1 mL of growing culture transferred to fresh media each passage.

Roll tube isolation was used to select for a single anaerobic fungal isolate (Haitjema *et al*. 2014). After 3 years of cultivation, chloramphenicol (prepared in 40 vol% molecular grade ethanol) was supplemented at each passage. Cultures were passaged in Hungate tubes with 9 mL Medium C (Davies *et al*. 1993), approximately 0.1 g reed canary grass, and chloramphenicol (100 µg/mL final concentration) until methane could no longer be detected via gas chromatograph (TRACE 1300, Thermo Fisher Scientific) and the culture did not appear turbid. After subsequent growth observed by pressure production and grass clumping, 0.1 mL of the culture supernatant was used to inoculate an anaerobic roll tube, and a fungal isolate was selected with the roll tube isolation method (Haitjema *et al*. 2014), with thallus picking performed inside an anaerobic chamber (cat. no. AS-580, Anaerobe Systems, Morgan Hill, California, USA). The roll tube was incubated for approximately 4 days until visible thalli formed on the agar surface. A single thallus was picked from the roll tube and inoculated into a Hungate tube containing 10 mL Medium C with 100 µg/mL chloramphenicol (and reed canary grass supplied as a carbon source). The roll tube isolation process was repeated 4 times to ensure axenic cultivation.

### Routine cultivation and culture media

Monocultures of *N. cameroonii* var. *constans* were routinely grown anaerobically at 39°C in Hungate tubes containing 9 mL of either MC– media (Peng *et al*. 2018) with vitamin supplement (0.1% v:v, ATCC cat. no. MD-VS, made in house or ordered from ATCC) or Medium C (Davies *et al*. 1993) and approximately 0.1 g lignocellulosic substrate (either dried, milled reed canary grass or sorghum). Cultures were passaged every 3–5 days, and 1 mL growing culture was used to inoculate fresh media.

### Morphological and phylogenetic characterization of the fungal isolate

Approximately 10–20 µL of growing fungal culture was prepared on a microscope slide and imaged using a Zeiss Primovert transmitted light microscope for morphological characterization (cat. no. 415510–1101-000, Carl Zeiss Microscopy, Oberkochen, Germany) with a 20× objective and a SPOT Idea 28.2 5-MP camera (SPOT Imaging, Sterling Heights, Michigan, USA). Spot 5.1 software was used to capture images and add scale bars. Fungal zoospores were identified based on cell size (cell bodies ranging from 5 to 10 μm), circular morphology, and movement with the presence of flagella.

The full ITS1-D1/D2 large ribosomal subunit (LSU) genomic region was amplified from genomic DNA extracted with the DNeasy PowerSoil Pro kit (Qiagen) according to manufacturer protocol, using primers ITS5 (5′-GGAAGTAAAAGTCGTAACAAGG-3′) and GG-NL4 (5′-TCAACATCCTAAGCGTAGGTA-3′) (Hanafy *et al*. 2020) and cloned into chemically competent DH5α *Escherichia coli* using the Invitrogen TOPO TA Cloning Kit for Subcloning. The D1/D2 LSU region was amplified from 7 colonies by colony PCR using Phusion polymerase and primers NL1 (5′- GCATATCAATAAGCGGAGGAAAAG-3′) and GG-NL4, and the amplicons were submitted for Sanger sequencing. Sequences were aligned with a reference data set of anaerobic fungal D1-D2 LSU sequences, obtained from the NCBI-GenBank nr database (Supplementary Table 1), using MUSCLE (Edgar 2004) with default parameters and manually refined in Geneious software. The generated alignment was used for constructing a maximum likelihood phylogenetic tree using IQ-TREE 2 (Hoang *et al*. 2018; Minh *et al*. 2020), with *Chytriomyces* sp. WB235A as the outgroup. A best fit substitution model (TN + F + G4) was chosen according to the Bayesian Information Criterion. Bootstrap values were calculated based on 1,000 replicates. The final tree was visualized and edited using the Interactive Tree of Life (iTOL) platform (Letunic and Bork 2021). Based on combined morphological and LSU analysis, the fungal isolate was named *N. cameroonii* var. *constans* because of phylogenetic clustering within the *N. cameroonii* clade (Supplementary Fig. 1).

### Harvesting tissue for DNA extractions

Fungal monocultures were grown for 3 days prior to harvest. They were cultivated in anaerobic serum bottles containing 80 mL MC– media (Peng *et al*. 2018), with 0.2 µm filtered vitamin supplement (0.1% v:v) and glucose (5 g/L) added post autoclaving. Ten bottles were combined and vacuum filtered through sterile miracloth and rinsed with Millipore water. The fungal mat was then removed from the miracloth with tweezers and put in a 50mL Falcon tube. It was flash frozen in liquid nitrogen and stored in a −80°C freezer until shipment to the Arizona Genome Institute on dry ice.

### Genomic DNA extraction and sequencing

DNA was extracted at the Arizona Genomics Institute using a modified cetyltrimethylammonium bromide protocol. DNA was then sequenced at the Joint Genome Institute (JGI) with the PacBio SEQUEL IIe using the protocol for 6–10 kb with BluePippin size selection with 1 × 1800 min sequencing movie times. The CCS reads were filtered for artifacts and then assembled with Flye version 2.9-b1768 [-t 32 –pacbio-hifi] (https://github.com/fenderglass/Flye) (Kolmogorov *et al*. 2019) and subsequently polished with 2 rounds of RACON version 1.4.13 racon [-u -t 36] (https://github.com/lbcb-sci/racon) (Vaser *et al*. 2017). The final genome assembly was annotated using the JGI Annotation pipeline (Grigoriev *et al*. 2014). GC% was quantified using the infoseq command in EMBOSS version 6.6.0 (Rice *et al*. 2000) for unmasked assemblies and CDS-only FASTA files downloaded from MycoCosm (Grigoriev *et al*. 2014).

### CAZyme gene annotation

Genomes were accessed and downloaded from MycoCosm (Grigoriev *et al*. 2014). For comparisons of CAZyme genome content and predictions across the fungal kingdom, dbCAN-sub (Zheng *et al*. 2023) was run using default parameters on all genomes on the California NanoSystems Institute's Pod cluster at UCSB, and the outputs containing CAZyme annotations and substrate predictions were parsed using custom R scripts. All unique substrates were summed during tabulation, even when multiple substrates were predicted for a given CAZyme. The prediction of multiple substrates may represent enzyme promiscuity, or, alternately, model ambiguity requiring experimental determination.

For tabulation of dockerin and scaffoldin domains in *N. cameroonii* var. constans, which is outside the scope of dbCAN-sub, HMMER searches (hmmer.org, version 3.1b2) (Finn *et al*. 2011) of the CDS aa.fasta file downloaded from MycoCosm were carried out using PF02013.hmm for dockerin and cohesin3.hmm. Annotations for CAZymes, for the enumeration of dockerins and carbohydrate-binding modules (CBMs) fused to enzymatic domains in the genome of *N. cameroonii* var. *constans*, were downloaded from MycoCosm (Grigoriev *et al*. 2014) and parsed using custom R scripts. All HMMER search models, CAZyme list, and open reading frame file are available at https://github.com/O-Malley-Lab/N_var_constans.

### Cultivation for RNA extraction, sequencing, and assembly

Hungate tubes containing 9 mL MC– media (Peng *et al*. 2018), vitamin supplement (0.1% v:v, made in house from ATCC cat. no. MD-VS recipe), and either glucose (5 g/L), cellobiose (5 g/L), filter paper (0.1 g, cut in strips), or reed canary grass (0.1 g, 1 mm sieved) (4 biological replicates per condition) were inoculated with 1 mL from a 3-day-old fungal culture grown on MC– with reed canary grass. Cultures were grown for 2–3 days and then poured into 50 mL Falcon tubes containing 10 mL RNAlater, made in-house (Malmstrom 2015; Erster *et al*. 2021) and centrifuged for 30 min at 10,000×g and 4°C using a fixed angle rotor (Eppendorf 5810 R, rotor F-34-6-38). After centrifugation, supernatant was decanted. Pellets were flash frozen in liquid nitrogen and stored at −80°C until RNA extraction.

RNA extractions were performed using the Qiagen RNeasy Mini Kit, following the manufacturer-provided protocol: “Purification of total RNA from plant cells and tissues and filamentous fungi” with the addition of the optional DNase on-column digest. Cells were lysed via liquid nitrogen grinding, and a QIAshredder was used to homogenize each sample. RNA was eluted in 50 µL of RNAse-free water, and the eluent was then re-eluted through the column to concentrate the RNA. RNA concentration was checked on the Invitrogen Qubit 2.0 fluorometer, with all samples > 25 ng/µL. Quality was evaluated with the Agilent 2200 TapeStation or 2100 Bioanalyzer—all samples had a RIN or RINe score above 7.

Extracted RNA was sent to the JGI and sequenced using the Illumina NovaSeq S4 with run type 2 × 151 bp. PCR was employed to make stranded sequencing libraries. The JGI used their pipeline for quality control; BBDuk removed artifacts from the 3′ end (settings: *k*-mer = 25, 1 mismatch allowed, phred trimming at Q6) and reads with Ns, PhiX, or RNA spike-in. Short reads were also removed (<1/3 of initial read length and/or <25 bp). Alignment to the newly sequenced genome was performed with HISAT2 (v. 2.2.1) (Kim *et al*. 2015); output files were sorted and indexed using SAMtools v. 1.7 (Li *et al*. 2009). deepTools (v. 3.1) was used to calculate strand-specific coverage (Ramírez *et al*. 2014), and raw gene counts were calculated with featureCounts v. 1.5.2 (settings: -s 2 -p –primary options) (Liao *et al*. 2014).

### Differential gene expression analysis, multidimensional scaling plot, and heatmap

The raw gene count matrix generated by featureCounts (see above) was used as the input for DESeq2 v. 1.40.2 (Love *et al*. 2014). Genes with 0 counts were removed prior to running DESeq2. After using the DESeq command, pairwise conditions were compared using the contrast function. Plots showing significantly regulated genes between the pairwise comparisons were made using the DESeq2 plotMA command with alpha set to 0.05 (Supplementary Fig. 2). Except for in this supplemental figure, genes with an average transcript per million (TPM) value less than 2 for at least 1 condition were excluded from further analysis. Code for calculating TPM values can be found at https://github.com/O-Malley-Lab/N_var_constans. Significance in analyses was set to a *P*-adjusted value < 0.05.

The multidimensional scaling (MDS) plot was created using the edgeR package in R (Robinson *et al*. 2010). After applying the TPM cutoff as in the preceding paragraph, the raw gene count matrix was normalized using trimmed-mean-of-M-values (TMM) normalization. The MDS plot was then generated from these counts using Euclidean distance via the plotMDS function.

The pheatmap package in R was used to generate the differential expression heatmaps. *P*-values from the filtered data set were used to generate a heatmap of 100 genes with the minimum of *P*-values across the 3 comparative conditions (cellobiose vs glucose, filter paper vs glucose, and reed canary grass vs glucose). The *P*-values for the condition of reed canary grass vs glucose dominate the minimum *P*-values used in this analysis. Additional heatmaps were therefore created using the minimum *P*-values for cellobiose vs glucose only (Supplementary Fig. 3), filter paper vs glucose only (Supplementary Fig. 4), and reed canary grass vs glucose only (Supplementary Fig. 5). Additionally, a heatmap of 100 CAZyme-annotated genes with the minimum of *P*-values across the 3 comparative conditions (cellobiose vs glucose, filter paper vs glucose, and reed canary grass vs glucose) was created (Supplementary Fig. 6). Gene-level annotations for CAZymes were downloaded from MycoCosm (Grigoriev *et al*. 2014).

### CAZyme structure predictions

Structures of highly differentially regulated proteins containing dockerin and/or CBM domains but lacking further enzymatic annotations were predicted using AlphaFold (Jumper *et al*. 2021), which was implemented on California NanoSystems Institute's high performance computing clusters. Proteins of interest were selected by sorting predicted CAZyme genes that had only dockerin and/or CBM domain annotations (MycoCosm) by lowest adjusted *P*-values for differential gene expression. The highest ranked structure prediction from AlphaFold for each protein was used for further analyses. The per-residue local distance difference test (pLDDT) confidence scores for the protein structure models were retrieved from the B-factor field of the coordinate section of the output pdb file and the final structures were visualized using UCSF ChimeraX (Meng *et al*. 2023). Structural homology searches for annotation were carried out using the Foldseek web interface (van Kempen *et al*. 2024). The protein sequences were also run on InterPro (Blum *et al*. 2025) for comparison.

### Co-cultivation of anaerobic fungi with methanogenic archaea and methane analysis

*Methanobrevibacter smithii* (DSM 861), *Methanobrevibacter thaueri* (DSM 11995), and *Methanobacterium bryantii* (DSM 863) were obtained from the DSMZ culture collection. These methanogens were revived from cryostocks and cultivated at 39°C on methanogen media, which is MC– medium (Peng *et al*. 2018) with the following additions per liter: 10 mL of 100× trace elements solution (made in house from the ATCC MD-TMS recipe), 2 g sodium acetate anhydrous, and 4 g sodium formate. Methanogen media was bubbled with carbon dioxide prior to aliquoting, and media was aliquoted under a flow of 80% hydrogen 20% carbon dioxide.

For co-cultivation, penicillin-streptomycin (Thermo Fisher Scientific cat. no. 15140122) was added post autoclaving (final concentrations of 100 units/mL penicillin and 100 μg/mL streptomycin) to a Hungate tube containing 0.1 g dry, milled reed canary grass and 9 mL Medium C (Davies *et al*. 1993) or MC– (Peng *et al*. 2018); 0.5 mL of methanogen-containing culture (see above) and 0.5 mL of anaerobic fungi-containing Medium C or MC– culture were then added. For comparing *N. cameroonii* var. *constans* co-cultures to *Neocallimastix californiae* co-cultures, MC– media was used; Medium C was used for the experiment comparing methanogen monocultures to co-cultures with *N. cameroonii* var. *constans*. Cultures were incubated at 39°C; after 4 days of co-culture growth, gas pressures were measured via pressure transducer, and 100 μL of headspace gas was injected into a Shimadzu GC-14A equipped with a flame ionization detector for methane quantification by comparison with a standard curve. Statistics were computed in GraphPad Prism v. 10.4.2.

## Results and discussion

### *N. cameroonii* var. *constans* was isolated from a mixed goat fecal consortium

*N. cameroonii* var. *constans* was isolated as part of an extensive enrichment study, where communities from goat feces were cultivated on different biomass substrates and characterized via whole-genome shotgun sequencing (Peng *et al*. 2021). *Neocallimastix* was the most abundant anaerobic fungal genus cultivated on the 3 different lignocellulosic substrates tested, and out of the 18 fungal metagenome-assembled genomes produced from the Peng *et al.* study, 12 are likely the same species as *N. californiae* (Peng *et al*. 2021) (also known as *N. cameroonii* var. *californiae*). The fungal isolate, *N. cameroonii* var. *constans*, is of particular interest because it belongs to that species and contributed to those MAGs, and because prior to isolation, it was stably cultivated for 3 years in a consortium composed mainly of fungi and methanogens (Peng *et al*. 2021). Given its longevity in cultivation compared to other anaerobic fungi, its capacity to survive cryopreservation, and its ability to stably pair with other anaerobes, it represents a robust strain amenable to long-term culture that was characterized further in this study.

**Fig. 1.**
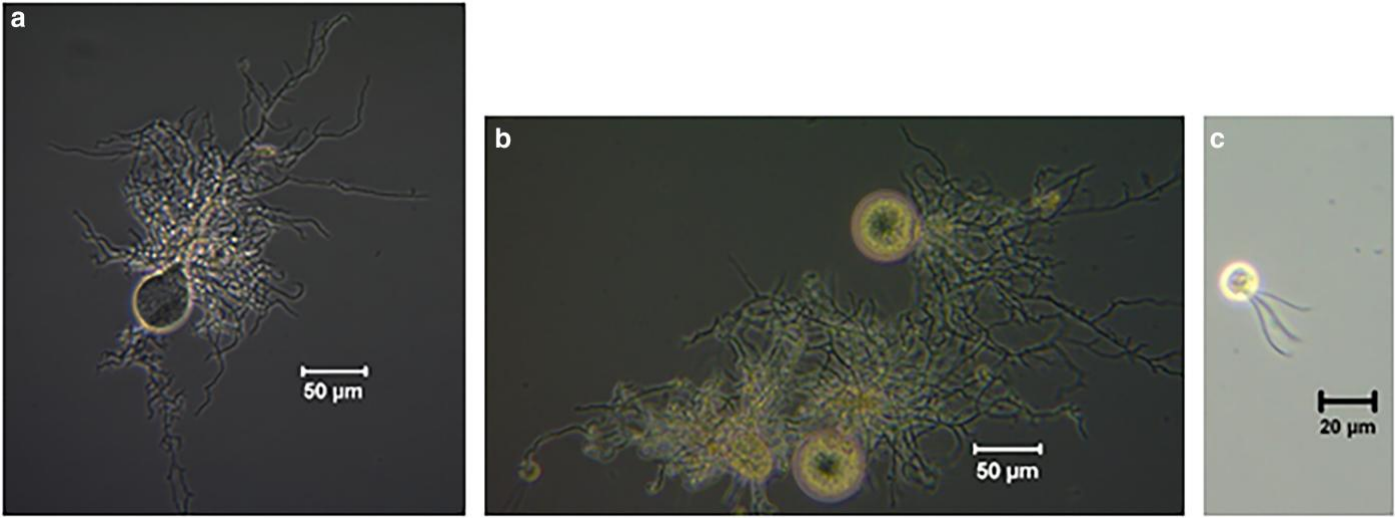
Microscopy shows monocentric thalli a and b), rhizoids a and b), and polyflagellate zoospore c) morphology of *N. cameroonii* var. *constans*.

This isolate, *N. cameroonii* var. *constans* (characterized as a variant of the *cameroonii/californiae* species), is shown in micrograph in Fig. 1, and its fungal structure, including sporangia, rhizoids, and zoospores, is all consistent with assignment to the *Neocallimastix* genus. *Neocallimastix* fungi have an extensive rhizoidal network and monocentric thalli (Orpin 1975; Hess *et al*. 2020). Like other anaerobic fungi, *N. cameroonii* var. *constans* has a genome of 187 Mbp that is very AT-rich. There is a broad range of copy number variation among genes, but no evidence of specific polyploidy. The GC% of the entire genome is 18.2%, which is within the 16–22% range reported for other anaerobic fungi (Wilken *et al*. 2020). For coding regions, the GC content is 28.1%. Sequencing statistics are presented in Table 1. *N. cameroonii* var. *constans* as isolated here was likely to be a member of the species complex containing other conspecific *Neocallimastix* variants in the enriched microbial goat communities and shares >99.5% ITS2 sequence identity with several *Neocallimastix* MAGs from Peng *et al*. (2021). To better understand the relationship between these species in relation to other *Neocallimastigomycota*, we analyzed LSU sequences (Hanafy *et al*. 2020) and built a phylogenic tree (Supplementary Fig. 1).

**Table 1.** Genome statistics for *N. cameroonii* var. *constans* and select other anaerobic fungi.

	*N. cameroonii* var. *constans*	*Anaeromyces robustus* (Haitjema *et al*. 2017)	*Caecomyces churrovis* (Brown *et al*. 2021)	*N. californiae* (Haitjema *et al*. 2017)	*Neocallimastix lanati* (Wilken *et al*. 2021)	*Piromyces finnis* (Haitjema *et al*. 2017)	*Piromyces* sp. UH3-1
Assembly size (Mbp)	187.1	71.69	165.5	193.0	201.0	56.46	84.1
Read coverage	61.03×	20×	88.81×	20×	62.05×	Not reported	33.02×
Scaffolds	558	1035	7737	1819	970	232	84
GC%	18.2%	16.3%	19.0%	18.2%	18.3%	21.2%	19.5%
Coding GC%	28.1%	26.2%	29.1%	28.4%	28.0%	28.2%	28.6%
Dockerin domain-containing proteins	543	281	421	439	632	234	596
Scaffoldins	96	32	41	60	98	23	101

### Numerous predicted CAZymes are encoded in the *N. cameroonii* var. *constans* genome

Characterizing the CAZyme repertoire for a given fungus is a valuable tool for predicting its ecology. *Batrachochytrium dendrobatidis*, the well-publicized frog pathogen, is notable for its genomic expansion of chitin-binding domains (Abramyan and Stajich 2012). *Mycena rebaudengoi*, which grows on dead and decaying plant matter in forests, contains 118 putative lignin-active enzymes. Similarly, anaerobic fungal isolates have many enzymes targeted to a broad range of carbohydrates, including xylan, beta-glucan, chitin, beta-mannan, pectin, beta-galactan, and starch.

**Fig. 2.**
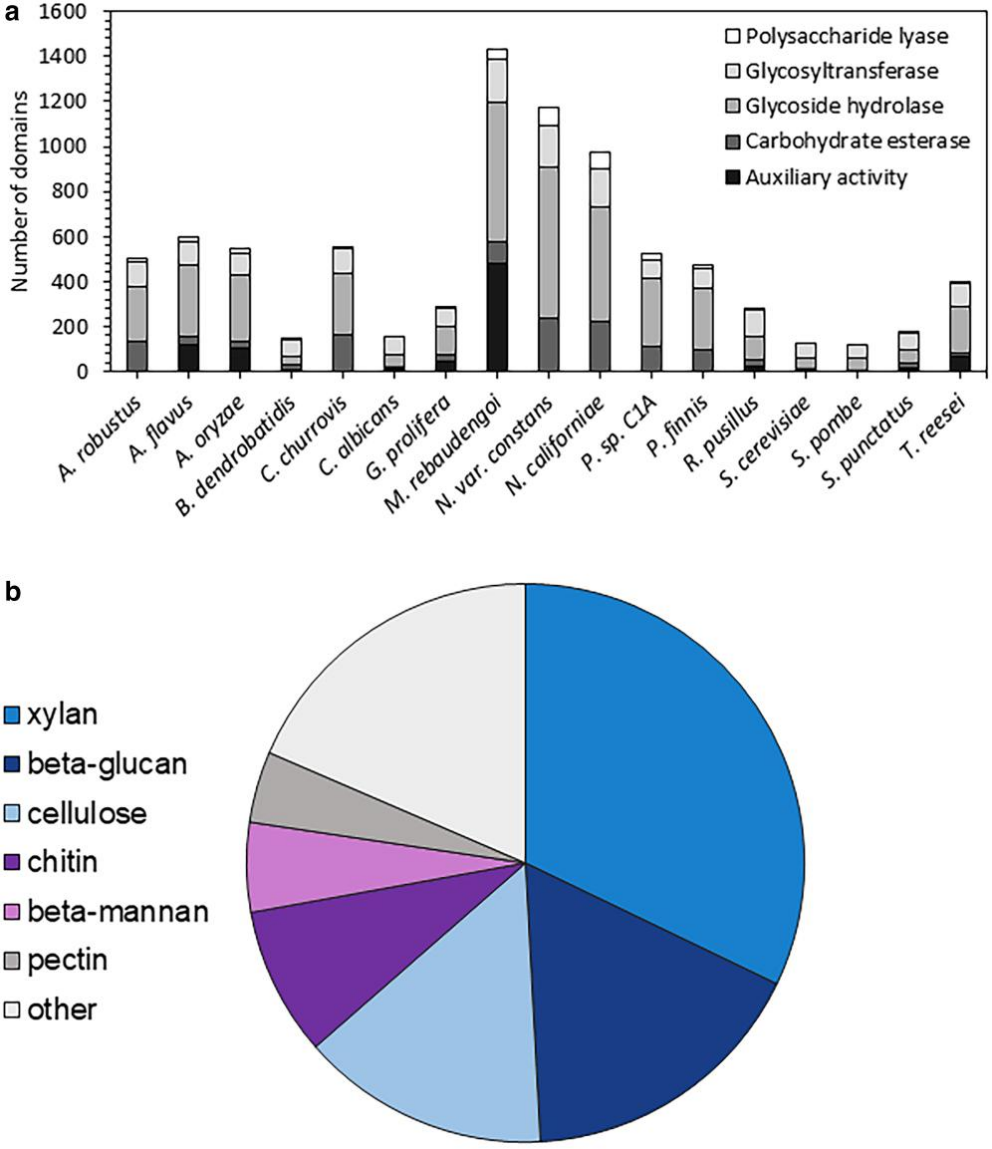
a) *N. cameroonii* var. *constans* has among the highest CAZyme domain content compared to other sequenced fungi. Abbreviations are as follows: *A. robustus* (*Anaeromyces robustus*), *A. flavus* (*Aspergillus flavus*), *A. oryzae* (*Aspergillus oryzae*), *B. dendrobatidis* (*Batrachochytrium dendrobatidis*), *C. churrovis* (*Caecomyces churrovis*), *C. albicans* (*Candida albicans*), *G. prolifera* (*Gonapodya prolifera*), *M. rebaudengoi* (*Mycena rebaudengoi*), *N.* var. *constans* (*N. cameroonii* var. *constans*), *N. californiae* (*Neocallimastix californiae*), *P.* sp. *C1A* (*Pecoramyces* sp. C1A), *P. finnis* (*Piromyces finnis*), *R. pusillus* (*Rhizomucor pusillus*), *S. cerevisiae* (*Saccharomyces cerevisiae*), *S. pombe* (*Schizosaccharomyces pombe*), *S. punctatus* (*Spizellomyces punctatus*), and *T. reesei* (*Trichoderma reesei*). Proteins were annotated using dbCAN3. *A. robustus*, *C. churrovis*, *N. cameroonii* var. *constans*, *N. californiae*, *P*. sp. C1A, and *P. finnis* are anaerobic gut fungi (*Neocallimastigomycota*), while the other fungi shown are not. b) Substrate prediction for CAZymes identified in *N. cameroonii* var. *constans* using dbCAN-sub. For clarity, the 30% of enzymes lacking a substrate prediction were omitted for the chart.

Using dbCAN3 (Zheng *et al*. 2023), we annotated CAZyme domains for comparison across fungi from various phyla. The closely related strain *N. californiae* (Haitjema *et al*. 2017) had very similar CAZyme composition and count as well as genome size (Fig. 2a). While the mushroom-forming basidiomycete *M. rebaudengoi* (Grigoriev *et al*. 2014) contained more total CAZymes, a large fraction of these were categorized as auxiliary activities, which consist of redox enzymes including lignin-active lytic polysaccharide mono-oxygenases among others (Cantarel *et al*. 2009). These observations confirm that anaerobic fungi contain the largest genomic repertoire of core CAZymes, consisting of carbohydrate esterases, glycoside hydrolases, glycosyltransferases, and polysaccharide lyases (Fig. 2).

With dbCAN-sub (Zheng *et al*. 2023), we further enumerated putative substrates for identified CAZymes in *N. cameroonii* var. *constans*. Substrate analysis confirmed extensive lignocellulose-degrading activity, with a near-majority (Fig. 2b) of CAZymes targeted at xylan, and beta-glucan and cellulose being 2 other highly represented substrates.

### CAZymes contain a broad range of cellulosome-associated features

Anaerobic fungi produce cellulosomes, which are multienzyme free or surface-anchored assemblies more widely studied in many cellulolytic bacteria (Doi *et al*. 2003; Artzi *et al*. 2017). Across species of anaerobic fungi, conserved dockerin domains are found in many CAZymes (Nagy *et al*. 2007), and these have been shown to play important roles in cellulosome assembly and biomass breakdown (Haitjema *et al*. 2017; Gilmore *et al*. 2020). In *N. cameroonii* var. *constans*, dockerin and CBM motifs are widespread, with the annotated genome containing 543 dockerin-containing proteins and 96 scaffoldins (Table 1). The distribution of dockerin domains also shows a marked CAZyme-class dependence (Table 2; Supplementary Table 16). Consistent with their presumed roles in the synthesis, as opposed to catabolism of carbohydrates, glycosyltransferases (GTs) in *N. cameroonii* var. *constans* are bereft of dockerins or CBMs (Table 2). Glycosyl hydrolases (GHs), however, frequently contain multiple dockerin domains (Supplementary Fig. 7), indicating key roles in lignocellulosic breakdown.

**Table 2.** Cellulosome and CAZyme annotations in *N. cameroonii* var. *constans*.

CAZyme class	Protein count	Proteins containing dockerin domains	Fraction containing dockerin domains	Proteins with annotated CBM	Fraction with CBM
Carbohydrate esterase (Ce)	160	20	0.13	64	0.40
Glycoside hydrolase (GH)	660	208	0.32	155	0.23
Glycosyltransferase (GT)	194	0	0	0	0.00
Polysaccharide lyase (PL)	85	2	0.02	34	0.40

Annotations downloaded from MycoCosm were used to quantify the fraction of CAZyme-containing open reading frames that also contained dockerins and/or carbohydrate-binding domains (CBMs). Note that Table 2 represents protein counts, not domain counts; i.e. some proteins have multiple domains. Statistics for 6 other anaerobic fungi are presented in Supplementary Table 16.

### Substrate complexity determines *N. cameroonii* var. *constans* CAZyme expression

**Fig. 3.**
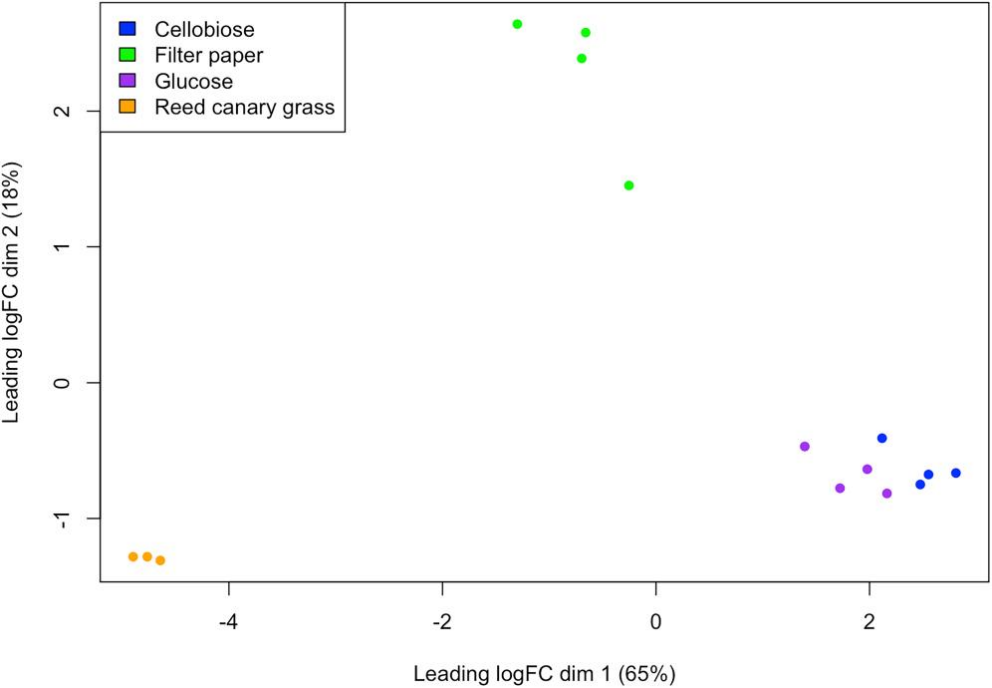
A MDS plot of *N. cameroonii* var. *constans* growth on 4 substrates shows that gene expression highly clusters by substrate type. The leading log-fold-change (leading logFC) is the root mean square average of the top 500 largest log2 fold changes between each sample pair.

MDS analysis showed that transcriptomic data from samples of *N. cameroonii* var. *constans* clustered significantly based on the substrate on which they were cultured (Fig. 3). The *N. cameroonii* var. *constans* samples grown on glucose, filter paper, and reed canary grass clustered far from each other on the MDS plot, while the samples grown on cellobiose clustered closely with those grown on glucose. As cellobiose is a disaccharide of glucose, *N. cameroonii* var. *constans* experiences similar gene expression in response to glucose and cellobiose.

**Fig. 4.**
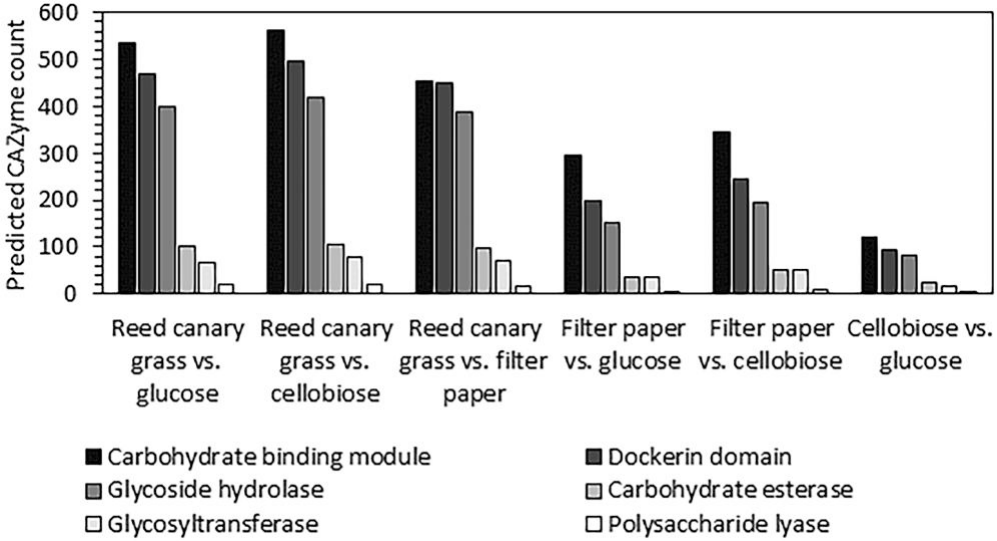
Numerous predicted CAZymes are differentially regulated across different carbon sources in *N. cameroonii* var. *constans*. For each substrate comparison, the number of genes containing at least 1 of the 6 CAZyme domains is enumerated. Genes are only included if they are significantly differentially expressed (*P*adj < 0.05) and have an average TPM greater than 2 for 1 or both conditions compared.

As expected, we found that CAZyme expression varied depending on the carbon substrate available to this fungal isolate. DESeq2 (Love *et al*. 2014) analysis showed that the highest levels of differentially expressed CAZymes are found when comparing reed canary grass (the most recalcitrant substrate tested) to the other less complex carbon sources (filter paper, cellobiose, and glucose) (Fig. 4; Supplementary Tables 2–13). Filter paper is more difficult to break down than cellobiose or glucose, which was reflected in hundreds of differentially expressed predicted CAZymes when comparing filter paper to cellobiose or glucose. Cellobiose cultures, with the closest structural similarity to glucose of the substrates, showed the most similar pattern of CAZyme expression to glucose.

**Fig. 5.**
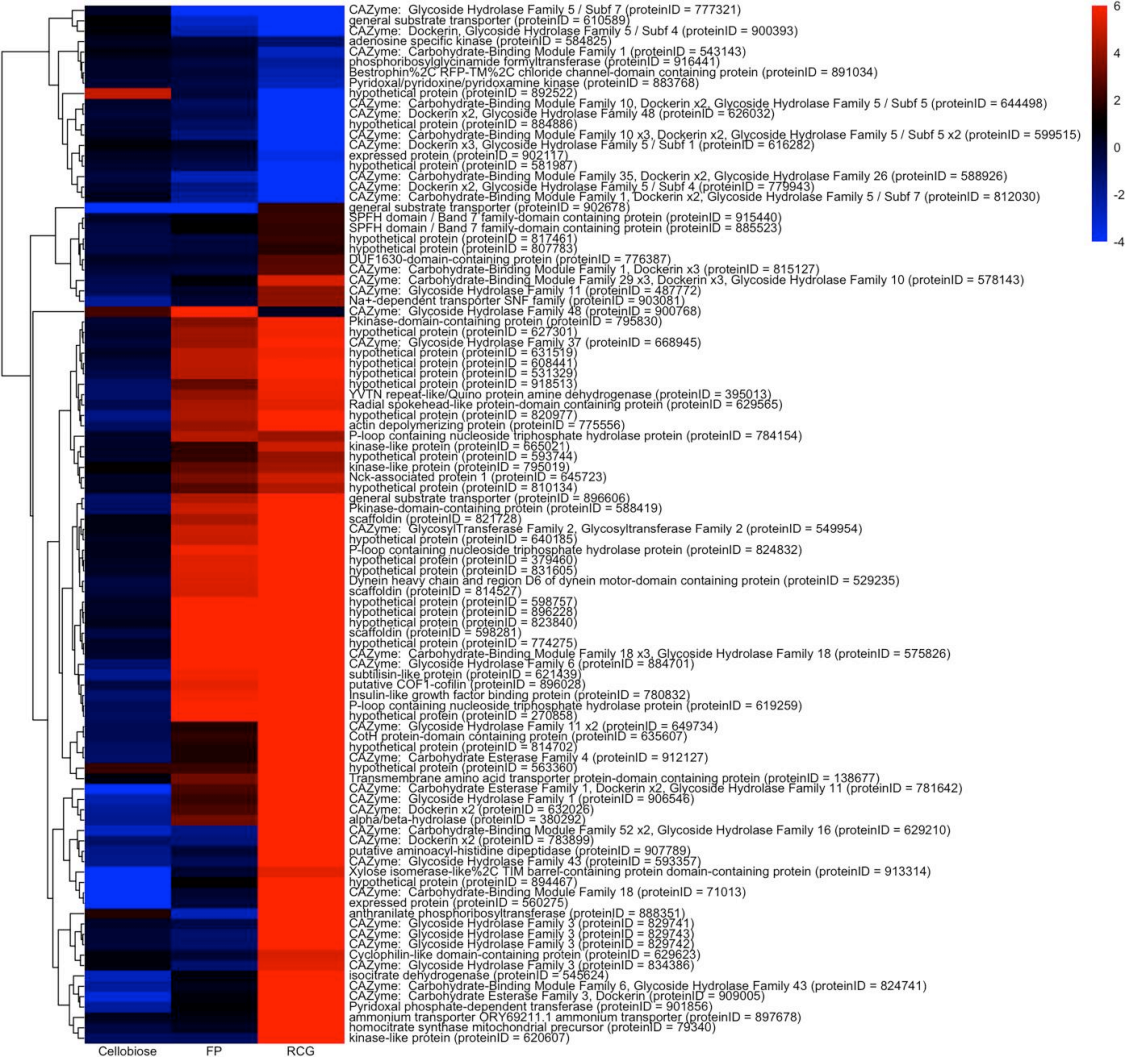
Differential expression analysis of *N. cameroonii* var. *constans* grown on cellobiose, filter paper (FP), or reed canary grass (RCG), each compared to glucose. Adjusted *P*-values were calculated for pairwise comparisons for each substrate (cellobiose, filter paper, reed canary grass) vs glucose. For each gene, the lowest *P*-adjusted value across each of these comparisons was designated as the minimum *P*-value “pmin” (all pmin values were <0.05), and genes with the 100 lowest pmin are shown in this heatmap. Colors correspond to the log2 fold change value of a predicted gene for each condition. For the genes annotated as CAZymes, domains are listed, with their predicted counts within that gene. For example, “Dockerin x3” indicates that 3 dockerin domains were identified by dbCAN3 in the associated gene. Gene ordering is based on expression similarity. ProteinID refers to the annotated MycoCosm designation.

Using DESeq2 (Love *et al*. 2014), we identified significantly differentially expressed genes for the different comparative growth conditions. Gene expression of *N. cameroonii* var. *constans* grown on reed canary grass, filter paper, and cellobiose was compared to that on glucose as a baseline. The number of significantly upregulated genes was highest in the reed canary grass/glucose comparison, followed by filter paper/glucose and then cellobiose/glucose. This is observed in both the all-genes data set (Fig. 5; Supplementary Figs. 3–5) and the CAZymes-only data set (Supplementary Fig. 6).

When comparing each growth substrate, pairwise comparisons showed substantially more differentially expressed genes between reed canary grass and each of the other substrates than pairwise comparisons among filter paper, cellobiose, and glucose (Supplementary Figs. 2, 8, and 9). Approximately 53–64% of expressed genes (with an average TPM > 2 for at least 1 condition) were significantly differentially expressed between reed canary grass and each of the other substrates, while only 8–28% were significantly differentially expressed for pairwise comparisons between filter paper, cellobiose, and glucose (Supplementary Fig. 9). In pairwise comparisons with reed canary grass, the majority (∼60–65%) of significantly differentially expressed genes were upregulated in reed canary grass cultures (Supplementary Fig. 9). These results confirm that more genes are upregulated in breaking down a complex lignocellulosic material and utilizing its components. However, it is important to note that reed canary grass cultures also contained the highest number of significantly *downregulated* genes relative to the other substrates (Supplementary Fig. 9), and interestingly, these downregulated genes also include numerous CAZymes (Supplementary Table 14). These observations are consistent with large-scale changes in gene regulation patterns occurring when grown on complex lignocellulosic material.

**Fig. 6.**
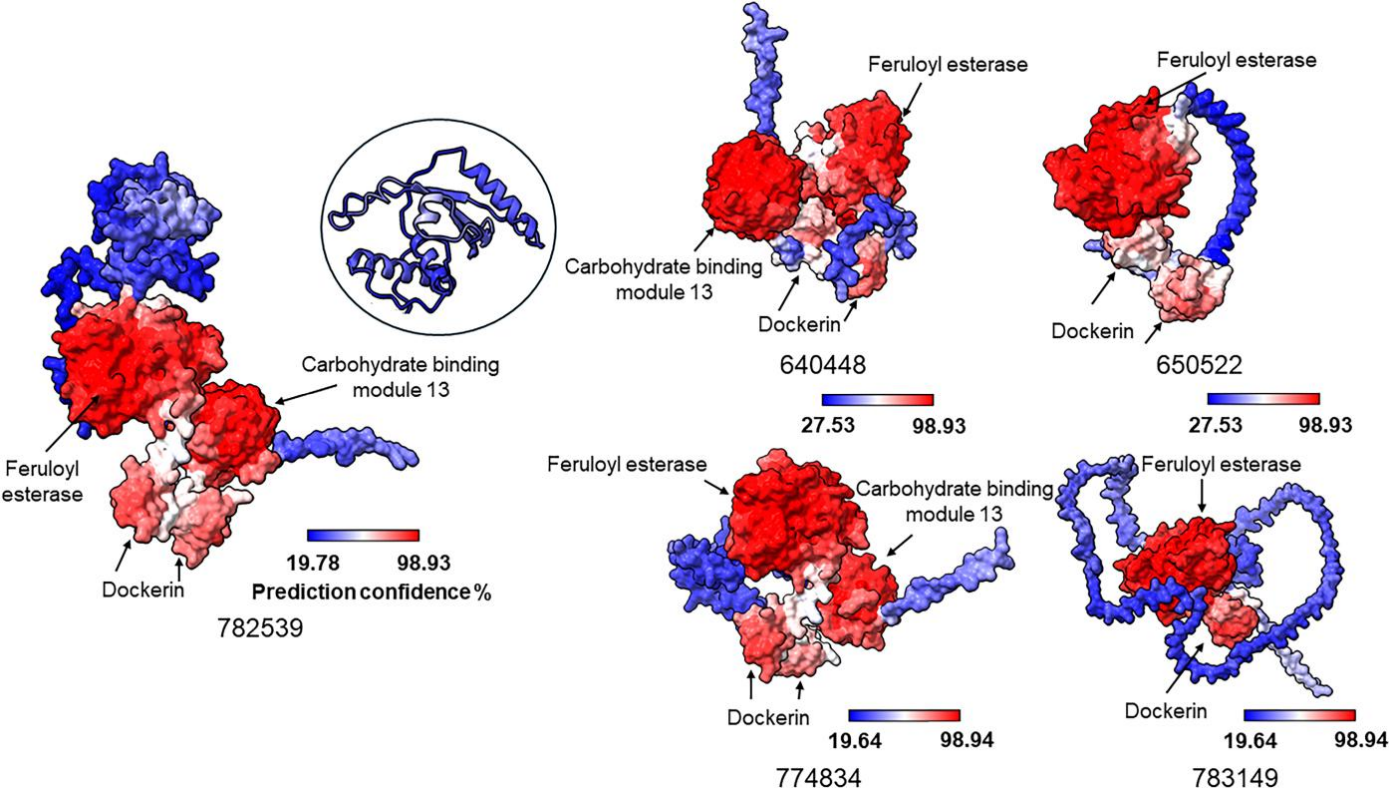
AlphaFold predicted structures show additional unannotated enzyme domains encoded by genes that were highly upregulated when cultures of *N. cameroonii* var. *constans* were grown on reed canary grass. Predicted structures for MycoCosm ProteinIDs: 782539, 640448, 650522, 774834, and 783149 are shown. Structural homology searching with Foldseek predicts that these proteins are feruloyl esterases. Supplementary Table 15 lists adjusted *P*-values and log fold change values for pairwise comparisons of these genes when cultures were grown on different substrates.

Since numerous genes found in anaerobic gut fungal genomes encode proteins of unknown function (Lankiewicz *et al*. 2022), such as those annotated as “hypothetical proteins,” it was hypothesized that there are unannotated CAZymes in the *N. cameroonii* var. *constans* genome. Specifically, many predicted CAZyme genes are annotated as containing 1 or more dockerin domains, but the majority of the protein length lacks specific enzymatic annotations. AlphaFold (Jumper *et al*. 2021) was used to better annotate proteins that were highly differentially regulated during growth on lignocellulosic substrate (reed canary grass). Multiple proteins were identified that appear to contain a feruloyl esterase domain along with dockerin domain(s), with some also containing a CBM 13 (CBM13) (Fig. 6). The CBM13 domain was first identified in plant lectins and can bind to sugars multivalently (Fujimoto 2013). In these proteins from *N. cameroonii* var. *constans*, sequence-based annotation only revealed the possible existence of the superfamily alpha/beta hydrolase fold (Fig. 6), which encompasses a wide range of catalytic and noncatalytic proteins across multiple domains of life (Holmquist 2000).

### Feruloyl esterases are structurally predicted in under-annotated CAZyme genes

AlphaFold (Jumper *et al*. 2021) and Foldseek (van Kempen *et al*. 2024) predicted the existence of feruloyl esterase domains in several under-annotated genes in this genome (Fig. 6). Feruloyl esterases catalyze bond scission between lignin and polysaccharides, an essential step in the metabolism of plant matter by anaerobic fungi (Dilokpimol *et al*. 2016). Furthermore, some level of structural (and sequence) diversity exists among these feruloyl esterases, with proteins containing a variable number of dockerins, CBMs, and, most interestingly, a lid-like domain (Fig. 6) which has been implicated in substrate binding (Suzuki *et al*. 2014; Perez-Garcia *et al*. 2023). However, this putative lid-like domain was not predicted with much confidence by AlphaFold (pLDDT < 50), though it seems to have some structural elements (4 alpha helices and 1 beta sheet), indicating it might be only structured in a multiprotein complex or stable under certain conditions such as in presence of calcium or substrate, which has been reported previously (Suzuki *et al*. 2014). A different low confidence element was predicted in protein 774834 (Fig. 6; Supplementary Table 15). Additionally, these predicted feruloyl esterases were upregulated in the presence of lignocellulosic biomass (reed canary grass), but not all were upregulated in the presence of cellulose-only filter paper (Supplementary Table 15), increasing confidence that these enzymes may be involved in the breaking of bonds at the interface of lignin and polysaccharides. The Foldseek prediction of feruloyl esterase domains in many of these is consistent with HMM-based annotation by PANTHER (Thomas *et al*. 2022) even though Superfamily searches (Pandurangan *et al*. 2019) only returned broad alpha-beta hydrolase classifications. The structure-based matching reported here shows broad core domain conservation but also reveals diverse additional protein modules that may be involved in modulating activity and/or localization. The sequence-level diversity of annotated and unannotated feruloyl esterases in this anerobic fungus genome is consistent with recent experimental support for multidomain feruloyl esterase-containing proteins in other anaerobic fungi (Shi *et al*. 2025). While the results presented here are primarily predictions of structure, they invite additional experimental efforts to further identify and characterize these putative feruloyl esterases with regard to protein abundance and substrate specificity.

### *N. cameroonii* var. *constans* and methanogen co-cultures convert lignocellulose to methane

**Fig. 7.**
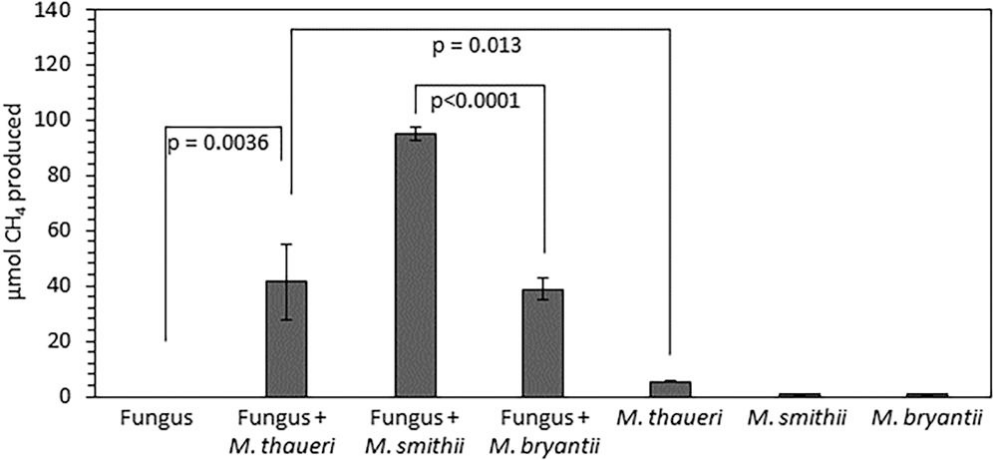
Methane production is significantly higher for co-cultures of *N. cameroonii* var. *constans* and methanogens compared to methanogen monocultures, which shows the ability of anaerobic fungi to degrade lignocellulose into fermentation products that methanogens can convert to methane. Methane was not detected in fungal monocultures. Error bars represent the standard deviation of 4 biological replicates. Statistical comparisons were made using Welch's *t*-test and other comparisons are omitted for clarity, with all co-culture to monoculture comparisons being statistically significant at *P* < 0.05.

*N. cameroonii* var. *constans* was isolated from a previously characterized microbial community, where the community produced methane as a major end product and maintained its membership and metabolic output for multiple years of cultivation (Peng *et al*. 2021). Anaerobic fungi and methanogens exist in commensal and symbiotic relationships in the rumen, with hydrogen produced by fungi being converted to methane by methanogenic archaea such as in natural consortia (Jin *et al*. 2011; Gilmore *et al*. 2019) and synthetic co-cultures (Swift *et al*. 2019; Leggieri *et al*. 2021). We hypothesized that *N. cameroonii* var. *constans* would pair with methanogens in synthetic co-cultures to convert lignocellulose to methane. Methane production was measured for fungal-methanogen co-cultures: 3 different methanogens were paired with *N. cameroonii* var. *constans* and *N. californiae* (Fig. 7; Supplementary Fig. 10). Productive partnership (evidenced by methane detection) was observed for all tested samples via the generation of methane from lignocellulosic substrates, with much higher methane outputs in co-culture compared to methanogen monoculture (Fig. 7; Supplementary Fig. 10). Low methane yield occurred in monoculture due to carryover from inoculum containing hydrogen and methane. In cultures grown on Medium C, higher methane production was observed in co-cultures with *M. smithii* compared with *M. bryantii* and *M. thauerii*, which produced similar methane levels (Fig. 7). These results are consistent with those observed by Peng *et al*. (2021), in which membership by both methanogenic archaea and fungi was important for methane production. *N. cameroonii* var. *constans* is therefore a versatile chassis organism—the strain can be cultivated in communities, both natural and synthetically designed to capture metabolic interactions found in the rumen.

## Conclusions

This new isolate, *N. cameroonii* var. *constans*, is a robust anaerobic fungal strain with a considerable CAZyme portfolio, with potentially many more enzymes involved in degradation than previously characterized. Structure-based prediction with AlphaFold revealed diverse, unannotated, catalytic domains motivating future experimental validation. This strain's ease of cultivation is supported by its growth on a variety of different substrates, including reed canary grass, filter paper, cellobiose, and glucose (Supplementary Fig. 11), with transcriptional analyses showing a clear impact of substrate on gene expression. Additionally, its ability to exist long term in stable, naturally derived consortia as well as pair with methanogens in synthetic communities helps elucidate its potential for further microbial community studies, to reveal complex metabolic and spatial interactions between anaerobic fungi and other rumen members. This novel strain is a strong candidate for an anaerobic fungal chassis, and as such, it offers a step toward illuminating complicated anaerobic microbial interactions and substrate degradation mechanisms.

## Supplementary Material

jkaf137_Supplementary_Data

## Data Availability

The genome assembly and annotations are available from MycoCosm (https://mycocosm.jgi.doe.gov/Neocon1), and this Whole Genome Shotgun project has been deposited at DDBJ/ENA/GenBank under the accession JBODTK000000000 with BioProject PRJNA1052201 and with SRA accession SRP483220. The version described in this paper is version JBODTK010000000. The transcriptome data have BioProjects PRJNA1062873 through PRJNA1062887. The transcriptome reads are deposited to SRA with accession numbers SRP483209 through SRP483218 and SRP557719 through SRP557723. Additional supporting files for RNA-seq analyses and CAZyme annotations are available at https://github.com/O-Malley-Lab/N_var_constans. The fungal strain, *N. cameroonii* var. *constans*, has been deposited to the Jena Microbial Resource Collection (JMRC) Germany with strain number JMRC:SF:015734. Supplemental material available at G3 online.

## References

[jkaf137-B1] Abramyan J, Stajich JE. 2012. Species-specific chitin-binding module 18 expansion in the amphibian pathogen *Batrachochytrium dendrobatidis*. mBio. 3(3):e00150-12. 10.1128/mBio.00150-12. 22718849 PMC3569864

[jkaf137-B2] Artzi L, Bayer EA, Moraïs S. 2017. Cellulosomes: bacterial nanomachines for dismantling plant polysaccharides. Nat Rev Microbiol. 15(2):83–95. 10.1038/nrmicro.2016.164. 27941816

[jkaf137-B3] Bai X, McMullan G, Scheres SHW. 2015. How cryo-EM is revolutionizing structural biology. Trends Biochem Sci. 40(1):49–57. 10.1016/j.tibs.2014.10.005. 25544475

[jkaf137-B4] Blum M, Andreeva A, Florentino LC, Chuguransky SR, Grego T, Hobbs E, Pinto BL, Orr A, Paysan-Lafosse T, Ponamareva I, et al 2025. InterPro: the protein sequence classification resource in 2025. Nucleic Acids Res. 53(D1):D444–D456. 10.1093/nar/gkae1082. 39565202 PMC11701551

[jkaf137-B5] Brown JL, Swift CL, Mondo SJ, Seppala S, Salamov A, Singan V, Henrissat B, Drula E, Henske JK, Lee S, et al 2021. Co-cultivation of the anaerobic fungus *Caecomyces churrovis* with *Methanobacterium bryantii* enhances transcription of carbohydrate binding modules, dockerins, and pyruvate formate lyases on specific substrates. Biotechnol Biofuels. 14(1):234. 10.1186/s13068-021-02083-w. 34893091 PMC8665504

[jkaf137-B6] Brownlee AG . 1989. Remarkably AT-rich genomic DNA from the anaerobic fungus *Neocallimastix*. Nucleic Acids Res. 17(4):1327–1335. 10.1093/nar/17.4.1327. 2922283 PMC331806

[jkaf137-B7] Cantarel BL, Coutinho PM, Rancurel C, Bernard T, Lombard V, Henrissat B. 2009. The carbohydrate-active EnZymes database (CAZy): an expert resource for glycogenomics. Nucleic Acids Res. 37(Database):D233–D238. 10.1093/nar/gkn663. 18838391 PMC2686590

[jkaf137-B8] Cheng YF, Edwards JE, Allison GG, Zhu W-Y, Theodorou MK. 2009. Diversity and activity of enriched ruminal cultures of anaerobic fungi and methanogens grown together on lignocellulose in consecutive batch culture. Bioresour Technol. 100(20):4821–4828. 10.1016/j.biortech.2009.04.031. 19467591

[jkaf137-B9] Davies DR, Theodorou MK, Lawrence MIG, Trinci APJ. 1993. Distribution of anaerobic fungi in the digestive tract of cattle and their survival in faeces. J Gen Microbiol. 139(6):1395–1400. 10.1099/00221287-139-6-1395. 8360630

[jkaf137-B10] Dementiev A, Lillington SP, Jin S, Kim Y, Jedrzejczak R, Michalska K, Joachimiak A, O’Malley MA. 2023. Structure and enzymatic characterization of CelD endoglucanase from the anaerobic fungus *Piromyces finnis*. Appl Microbiol Biotechnol. 107(19):5999–6011. 10.1007/s00253-023-12684-0. 37548665 PMC10485095

[jkaf137-B11] Dilokpimol A, Mäkelä MR, Aguilar-Pontes MV, Benoit-Gelber I, Hildén KS, de Vries RP. 2016. Diversity of fungal feruloyl esterases: updated phylogenetic classification, properties, and industrial applications. Biotechnol Biofuels. 9(1):231. 10.1186/s13068-016-0651-6. 27795736 PMC5084320

[jkaf137-B12] Doi RH, Kosugi A, Murashima K, Tamaru Y, Han SO. 2003. Cellulosomes from mesophilic bacteria. J Bacteriol. 185(20):5907–5914. 10.1128/JB.185.20.5907-5914.2003. 14526000 PMC225047

[jkaf137-B13] Edgar RC . 2004. MUSCLE: a multiple sequence alignment method with reduced time and space complexity. BMC Bioinformatics. 5(1):113. 10.1186/1471-2105-5-113. 15318951 PMC517706

[jkaf137-B14] Edwards JE, Forster RJ, Callaghan TM, Dollhofer V, Dagar SS, Cheng Y, Chang J, Kittelmann S, Fliegerova K, Puniya AK, et al 2017. PCR and omics based techniques to study the diversity, ecology and biology of anaerobic fungi: insights, challenges and opportunities. Front Microbiol. 8:1657. 10.3389/fmicb.2017.01657. 28993761 PMC5622200

[jkaf137-B15] Erster O, Shkedi O, Benedek G, Zilber E, Varkovitzky I, Shirazi R, Oriya Shorka D, Cohen Y, Bar T, Yechieli R, et al 2021. Improved sensitivity, safety, and rapidity of COVID-19 tests by replacing viral storage solution with lysis buffer. PLoS One. 16(3):e0249149. 10.1371/journal.pone.0249149. 33784369 PMC8009371

[jkaf137-B16] Finn RD, Clements J, Eddy SR. 2011. HMMER web server: interactive sequence similarity searching. Nucleic Acids Res. 39(suppl_2):W29–W37. 10.1093/nar/gkr367. 21593126 PMC3125773

[jkaf137-B17] Fujimoto Z . 2013. Structure and function of carbohydrate-binding module families 13 and 42 of glycoside hydrolases, comprising a β-trefoil fold. Biosci Biotechnol Biochem. 77(7):1363–1371. 10.1271/bbb.130183. 23832347

[jkaf137-B18] Gilmore SP, Lankiewicz TS, Wilken SE, Brown JL, Sexton JA, Henske JK, Theodorou MK, Valentine DL, O’Malley MA. 2019. Top-down enrichment guides in formation of synthetic microbial consortia for biomass degradation. ACS Synth Biol. 8(9):2174–2185. 10.1021/acssynbio.9b00271. 31461261

[jkaf137-B19] Gilmore SP, Lillington SP, Haitjema CH, de Groot R, O’Malley MA. 2020. Designing chimeric enzymes inspired by fungal cellulosomes. Synth Syst Biotechnol. 5(1):23–32. 10.1016/j.synbio.2020.01.003. 32083193 PMC7015840

[jkaf137-B20] Grigoriev IV, Nikitin R, Haridas S, Kuo A, Ohm R, Otillar R, Riley R, Salamov A, Zhao X, Korzeniewski F, et al 2014. MycoCosm portal: gearing up for 1000 fungal genomes. Nucleic Acids Res. 42(D1):D699–D704. 10.1093/nar/gkt1183. 24297253 PMC3965089

[jkaf137-B21] Gruninger RJ, Nguyen TTM, Reid ID, Yanke JL, Wang P, Abbott DW, Tsang A, McAllister T. 2018. Application of transcriptomics to compare the carbohydrate active enzymes that are expressed by diverse genera of anaerobic fungi to degrade plant cell wall carbohydrates. Front Microbiol. 9:1581. 10.3389/fmicb.2018.01581. 30061875 PMC6054980

[jkaf137-B22] Haitjema CH, Gilmore SP, Henske JK, Solomon KV, de Groot R, Kuo A, Mondo SJ, Salamov AA, LaButti K, Zhao Z, et al 2017. A parts list for fungal cellulosomes revealed by comparative genomics. Nat Microbiol. 2(8):17087. 10.1038/nmicrobiol.2017.87. 28555641

[jkaf137-B23] Haitjema CH, Solomon KV, Henske JK, Theodorou MK, O’Malley MA. 2014. Anaerobic gut fungi: advances in isolation, culture, and cellulolytic enzyme discovery for biofuel production. Biotechnol Bioeng. 111(8):1471–1482. 10.1002/bit.25264. 24788404

[jkaf137-B24] Hanafy RA, Dagar SS, Griffith GW, Pratt CJ, Youssef NH, Elshahed MS. 2022. Taxonomy of the anaerobic gut fungi (*Neocallimastigomycota*): a review of classification criteria and description of current taxa. Int J Syst Evol Microbiol. 72(7):005322. 10.1099/ijsem.0.005322. 35776761

[jkaf137-B25] Hanafy RA, Johnson B, Youssef NH, Elshahed MS. 2020. Assessing anaerobic gut fungal diversity in herbivores using D1/D2 large ribosomal subunit sequencing and multi-year isolation. Environ Microbiol. 22(9):3883–3908. 10.1111/1462-2920.15164. 32656919

[jkaf137-B26] Hanafy RA, Wang Y, Stajich JE, Pratt CJ, Youssef NH, Elshahed MS. 2023. Phylogenomic analysis of the *Neocallimastigomycota*: proposal of *Caecomycetaceae* fam. nov., *Piromycetaceae* fam. nov., and emended description of the families *Neocallimastigaceae* and *Anaeromycetaceae*. Int J Syst Evol Microbiol. 73(2):005735. 10.1099/ijsem.0.005735. 36827202

[jkaf137-B27] Henske JK, Gilmore SP, Knop D, Cunningham FJ, Sexton JA, Smallwood CR, Shutthanandan V, Evans JE, Theodorou MK, O’Malley MA. 2017. Transcriptomic characterization of *Caecomyces churrovis*: a novel, non-rhizoid-forming lignocellulolytic anaerobic fungus. Biotechnol Biofuels. 10(1):305. 10.1186/s13068-017-0997-4. 29270219 PMC5737911

[jkaf137-B28] Hess M, Paul SS, Puniya AK, van der Giezen M, Shaw C, Edwards JE, Fliegerová K. 2020. Anaerobic fungi: past, present, and future. Front Microbiol. 11:584893. 10.3389/fmicb.2020.584893. 33193229 PMC7609409

[jkaf137-B29] Hoang DT, Chernomor O, von Haeseler A, Minh BQ, Vinh LS. 2018. UFBoot2: improving the ultrafast bootstrap approximation. Mol Biol Evol. 35(2):518–522. 10.1093/molbev/msx281. 29077904 PMC5850222

[jkaf137-B30] Holmquist M . 2000. Alpha/beta-hydrolase fold enzymes: structures, functions and mechanisms. Curr Protein Pept Sci. 1(2):209–235. 10.2174/1389203003381405. 12369917

[jkaf137-B31] Hooker CA, Lee KZ, Solomon KV. 2019. Leveraging anaerobic fungi for biotechnology. Curr Opin Biotechnol. 59:103–110. 10.1016/j.copbio.2019.03.013. 31005803

[jkaf137-B32] Jin W, Cheng Y-F, Mao S-Y, Zhu W-Y. 2011. Isolation of natural cultures of anaerobic fungi and indigenously associated methanogens from herbivores and their bioconversion of lignocellulosic materials to methane. Bioresour Technol. 102(17):7925–7931. 10.1016/j.biortech.2011.06.026. 21719276

[jkaf137-B33] Jumper J, Evans R, Pritzel A, Green T, Figurnov M, Ronneberger O, Tunyasuvunakool K, Bates R, Žídek A, Potapenko A, et al 2021. Highly accurate protein structure prediction with AlphaFold. Nature. 596(7873):583–589. 10.1038/s41586-021-03819-2. 34265844 PMC8371605

[jkaf137-B34] Kim D, Langmead B, Salzberg SL. 2015. HISAT: a fast spliced aligner with low memory requirements. Nat Methods. 12(4):357–360. 10.1038/nmeth.3317. 25751142 PMC4655817

[jkaf137-B35] Kolmogorov M, Yuan J, Lin Y, Pevzner PA. 2019. Assembly of long, error-prone reads using repeat graphs. Nat Biotechnol. 37(5):540–546. 10.1038/s41587-019-0072-8. 30936562

[jkaf137-B36] Lankiewicz TS, Lillington SP, O’Malley MA. 2022. Enzyme discovery in anaerobic fungi (Neocallimastigomycetes) enables lignocellulosic biorefinery innovation. Microbiol Mol Biol Rev. 86(4):e00041-22. 10.1128/mmbr.00041-22. 35852448 PMC9769567

[jkaf137-B37] Leggieri PA, Kerdman-Andrade C, Lankiewicz TS, Valentine MT, O’Malley MA. 2021. Non-destructive quantification of anaerobic gut fungi and methanogens in co-culture reveals increased fungal growth rate and changes in metabolic flux relative to mono-culture. Microb Cell Fact. 20(1):199. 10.1186/s12934-021-01684-2. 34663313 PMC8522008

[jkaf137-B38] Letunic I, Bork P. 2021. Interactive Tree Of Life (iTOL) v5: an online tool for phylogenetic tree display and annotation. Nucleic Acids Res. 49(W1):W293–W296. 10.1093/nar/gkab301. 33885785 PMC8265157

[jkaf137-B39] Li H, Handsaker B, Wysoker A, Fennell T, Ruan J, Homer N, Marth G, Abecasis G, Durbin R; 1000 Genome Project Data Processing Subgroup. 2009. The sequence alignment/map format and SAMtools. Bioinformatics. 25(16):2078–2079. 10.1093/bioinformatics/btp352. 19505943 PMC2723002

[jkaf137-B40] Li Y, Meng Z, Xu Y, Shi Q, Ma Y, Aung M, Cheng Y, Zhu W. 2021. Interactions between anaerobic fungi and methanogens in the rumen and their biotechnological potential in biogas production from lignocellulosic materials. Microorganisms. 9(1):190. 10.3390/microorganisms9010190. 33477342 PMC7830786

[jkaf137-B41] Liao Y, Smyth GK, Shi W. 2014. featureCounts: an efficient general purpose program for assigning sequence reads to genomic features. Bioinformatics. 30(7):923–930. 10.1093/bioinformatics/btt656. 24227677

[jkaf137-B42] Lillington SP, Hamilton M, Cheng J-F, Yoshikuni Y, O’Malley MA. 2023. Expression and characterization of spore coat CotH kinases from the cellulosomes of anaerobic fungi *(Neocallimastigomycetes)*. Protein Expr Purif. 210:106323. 10.1016/j.pep.2023.106323. 37331410

[jkaf137-B43] Liu Z, Wen S, Wu G, Wu H. 2022. Heterologous expression and characterization of *Anaeromyces robustus* xylanase and its use in bread making. Eur Food Res Technol. 248(9):2311–2324. 10.1007/s00217-022-04047-2.

[jkaf137-B44] Love MI, Huber W, Anders S. 2014. Moderated estimation of fold change and dispersion for RNA-Seq data with DESeq2. Genome Biol. 15(12):550. 10.1186/s13059-014-0550-8. 25516281 PMC4302049

[jkaf137-B45] Lyu Z, Shao N, Akinyemi T, Whitman WB. 2018. Methanogenesis. Curr Biol. 28(13):R727–R732. 10.1016/j.cub.2018.05.021. 29990451

[jkaf137-B46] Malmstrom R . 2015. RNAlater Recipe. Available from protocols.io. [accessed 2024 Dec 6]. 10.17504/protocols.io.c56y9d.

[jkaf137-B47] Meng EC, Goddard TD, Pettersen EF, Couch GS, Pearson ZJ, Morris JH, Ferrin TE. 2023. UCSF ChimeraX: tools for structure building and analysis. Protein Sci. 32(11):e4792. 10.1002/pro.4792. 37774136 PMC10588335

[jkaf137-B48] Minh BQ, Schmidt HA, Chernomor O, Schrempf D, Woodhams MD, von Haeseler A, Lanfear R. 2020. IQ-TREE 2: new models and efficient methods for phylogenetic inference in the genomic era. Mol Biol Evol. 37(5):1530–1534. 10.1093/molbev/msaa015. 32011700 PMC7182206

[jkaf137-B49] Nagy T, Tunnicliffe RB, Higgins LD, Walters C, Gilbert HJ, Williamson MP. 2007. Characterization of a double dockerin from the cellulosome of the anaerobic fungus *Piromyces equi*. J Mol Biol. 373(3):612–622. 10.1016/j.jmb.2007.08.007. 17869267

[jkaf137-B50] Orpin CG . 1975. Studies on the rumen flagellate *Neocallimastix frontalis*. J Gen Microbiol. 91(2):249–262. 10.1099/00221287-91-2-249. 1462

[jkaf137-B51] Pandurangan AP, Stahlhacke J, Oates ME, Smithers B, Gough J. 2019. The SUPERFAMILY 2.0 database: a significant proteome update and a new webserver. Nucleic Acids Res. 47(D1):D490–D494. 10.1093/nar/gky1130. 30445555 PMC6324026

[jkaf137-B52] Peng X, Swift CL, Theodorou MK, O’Malley MA. 2018. Methods for genomic characterization and maintenance of anaerobic fungi. In: de Vries RP, Tsang A, Grigoriev IV, editors. Fungal Genomics. Vol. 1775. 2nd ed. New York (NY): Humana Press. p. 53–67. (Methods in Molecular Biology). 10.1007/978-1-4939-7804-5_5 29876808

[jkaf137-B53] Peng X, Wilken SE, Lankiewicz TS, Gilmore SP, Brown JL, Henske JK, Swift CL, Salamov A, Barry K, Grigoriev IV, et al 2021. Genomic and functional analyses of fungal and bacterial consortia that enable lignocellulose breakdown in goat gut microbiomes. Nat Microbiol. 6(4):499–511. 10.1038/s41564-020-00861-0. 33526884 PMC8007473

[jkaf137-B54] Perez-Garcia P, Chow J, Costanzi E, Gurschke M, Dittrich J, Dierkes RF, Molitor R, Applegate V, Feuerriegel G, Tete P, et al 2023. An archaeal lid-containing feruloyl esterase degrades polyethylene terephthalate. Commun Chem. 6(1):193. 10.1038/s42004-023-00998-z. 37697032 PMC10495362

[jkaf137-B55] Perli T, Vos AM, Bouwknegt J, Dekker WJC, Wiersma SJ, Mooiman C, Ortiz-Merino RA, Daran J-M, Pronk JT. 2021. Identification of oxygen-independent pathways for pyridine nucleotide and coenzyme A synthesis in anaerobic fungi by expression of candidate genes in yeast. mBio. 12(3):e00967-21. 10.1128/mBio.00967-21. 34154398 PMC8262920

[jkaf137-B56] Podolsky IA, Seppälä S, Xu H, Jin Y-S, O’Malley MA. 2021. A SWEET surprise: anaerobic fungal sugar transporters and chimeras enhance sugar uptake in yeast. Metab Eng. 66:137–147. 10.1016/j.ymben.2021.04.009. 33887459

[jkaf137-B57] Ramírez F, Dündar F, Diehl S, Grüning BA, Manke T. 2014. deepTools: a flexible platform for exploring deep-sequencing data. Nucleic Acids Res. 42(W1):W187–W191. 10.1093/nar/gku365. 24799436 PMC4086134

[jkaf137-B58] Rice P, Longden I, Bleasby A. 2000. EMBOSS: the European molecular biology open software suite. Trends Genet. 16(6):276–277. 10.1016/s0168-9525(00)02024-2. 10827456

[jkaf137-B59] Robinson MD, McCarthy DJ, Smyth GK. 2010. Edger: a Bioconductor package for differential expression analysis of digital gene expression data. Bioinformatics. 26(1):139–140. 10.1093/bioinformatics/btp616. 19910308 PMC2796818

[jkaf137-B60] Seppälä S, Yoo JI, Yur D, O’Malley MA. 2019. Heterologous transporters from anaerobic fungi bolster fluoride tolerance in Saccharomyces cerevisiae. Metab Eng Commun. 9:e00091. 10.1016/j.mec.2019.e00091. 31016136 PMC6475669

[jkaf137-B61] Shi Q, Ma J, Abdel-Hamid AM, Li Y, Zhong P, Wang D, Sun Z, Tu T, Zhu W, Cheng Y, et al 2025. Mining of latent feruloyl esterase resources in rumen and insight into dual-functional feruloyl esterase-xylanase from *Pecoramyces ruminantium F1*. Bioresour Technol. 418:131854. 10.1016/j.biortech.2024.131854. 39617352

[jkaf137-B62] Solomon KV, Haitjema CH, Henske JK, Gilmore SP, Borges-Rivera D, Lipzen A, Brewer HM, Purvine SO, Wright AT, Theodorou MK, et al 2016. Early-branching gut fungi possess a large, comprehensive array of biomass-degrading enzymes. Science. 351(6278):1192–1195. 10.1126/science.aad1431. 26912365 PMC5098331

[jkaf137-B63] Suzuki K, Hori A, Kawamoto K, Thangudu RR, Ishida T, Igarashi K, Samejima M, Yamada C, Arakawa T, Wakagi T, et al 2014. Crystal structure of a feruloyl esterase belonging to the tannase family: a disulfide bond near a catalytic triad. Proteins. 82(10):2857–2867. 10.1002/prot.24649. 25066066

[jkaf137-B64] Swift CL, Brown JL, Seppälä S, O’Malley MA. 2019. Co-cultivation of the anaerobic fungus *Anaeromyces robustus* with *Methanobacterium bryantii* enhances transcription of carbohydrate active enzymes. J Ind Microbiol Biotechnol. 46(9-10):1427–1433. 10.1007/s10295-019-02188-0. 31089985

[jkaf137-B65] Swift CL, Louie KB, Bowen BP, Olson HM, Purvine SO, Salamov A, Mondo SJ, Solomon KV, Wright AT, Northen TR, et al 2021a. Anaerobic gut fungi are an untapped reservoir of natural products. Proc Natl Acad Sci U S A. 118(18):e2019855118. 10.1073/pnas.2019855118. 33906945 PMC8106346

[jkaf137-B66] Swift CL, Malinov NG, Mondo SJ, Salamov A, Grigoriev IV, O’Malley MA. 2021b. A genomic catalog of stress response genes in anaerobic fungi for applications in bioproduction. Front Fungal Biol. 2:708358. 10.3389/ffunb.2021.708358. 37744151 PMC10512342

[jkaf137-B67] Tapio I, Snelling TJ, Strozzi F, Wallace RJ. 2017. The ruminal microbiome associated with methane emissions from ruminant livestock. J Animal Sci Biotechnol. 8(1):7. 10.1186/s40104-017-0141-0. PMC5244708 28123698

[jkaf137-B68] Thomas PD, Ebert D, Muruganujan A, Mushayahama T, Albou L, Mi H. 2022. PANTHER: making genome-scale phylogenetics accessible to all. Protein Sci. 31(1):8–22. 10.1002/pro.4218. 34717010 PMC8740835

[jkaf137-B69] van Kempen M, Kim SS, Tumescheit C, Mirdita M, Lee J, Gilchrist CLM, Söding J, Steinegger M. 2024. Fast and accurate protein structure search with Foldseek. Nat Biotechnol. 42(2):243–246. 10.1038/s41587-023-01773-0. 37156916 PMC10869269

[jkaf137-B70] Vaser R, Sović I, Nagarajan N, Šikić M. 2017. Fast and accurate de novo genome assembly from long uncorrected reads. Genome Res. 27(5):737–746. 10.1101/gr.214270.116. 28100585 PMC5411768

[jkaf137-B71] Wilken SE, Monk JM, Leggieri PA, Lawson CE, Lankiewicz TS, Seppälä S, Daum CG, Jenkins J, Lipzen AM, Mondo SJ, et al 2021. Experimentally validated reconstruction and analysis of a genome-scale metabolic model of an anaerobic Neocallimastigomycota fungus. mSystems. 6(1):e00002–e00021. 10.1128/mSystems.00002-21. 33594000 PMC8561657

[jkaf137-B72] Wilken SE, Seppälä S, Lankiewicz TS, Saxena M, Henske JK, Salamov AA, Grigoriev IV, O’Malley MA. 2020. Genomic and proteomic biases inform metabolic engineering strategies for anaerobic fungi. Metab Eng Commun. 10:e00107. 10.1016/j.mec.2019.e00107. 31799118 PMC6883316

[jkaf137-B73] Wilken SE, Swift CL, Podolsky IA, Lankiewicz TS, Seppälä S, O’Malley MA. 2019. Linking ‘omics’ to function unlocks the biotech potential of non-model fungi. Curr Opin Syst Biol. 14:9–17. 10.1016/j.coisb.2019.02.001.

[jkaf137-B74] Youssef NH, Couger MB, Struchtemeyer CG, Liggenstoffer AS, Prade RA, Najar FZ, Atiyeh HK, Wilkins MR, Elshahed MS. 2013. The genome of the anaerobic fungus *Orpinomyces* sp. strain C1A reveals the unique evolutionary history of a remarkable plant biomass degrader. Appl Environ Microbiol. 79(15):4620–4634. 10.1128/AEM.00821-13. 23709508 PMC3719515

[jkaf137-B75] Zheng J, Ge Q, Yan Y, Zhang X, Huang L, Yin Y. 2023. dbCAN3: automated carbohydrate-active enzyme and substrate annotation. Nucleic Acids Res. 51(W1):W115–W121. 10.1093/nar/gkad328. 37125649 PMC10320055

