# Supplementary material for: Genomic and transcriptomic characterization of carbohydrate-active enzymes in the anaerobic fungus Neocallimastix cameroonii var. constans
Source: G3 (Bethesda). 2025 Jun 16;15(8):jkaf137. doi: 10.1093/g3journal/jkaf137 (PMC12341934; doi:10.1093/g3journal/jkaf137)
Supplement: jkaf137_Supplementary_Data [file jkaf137_supplementary_data.docx]

**
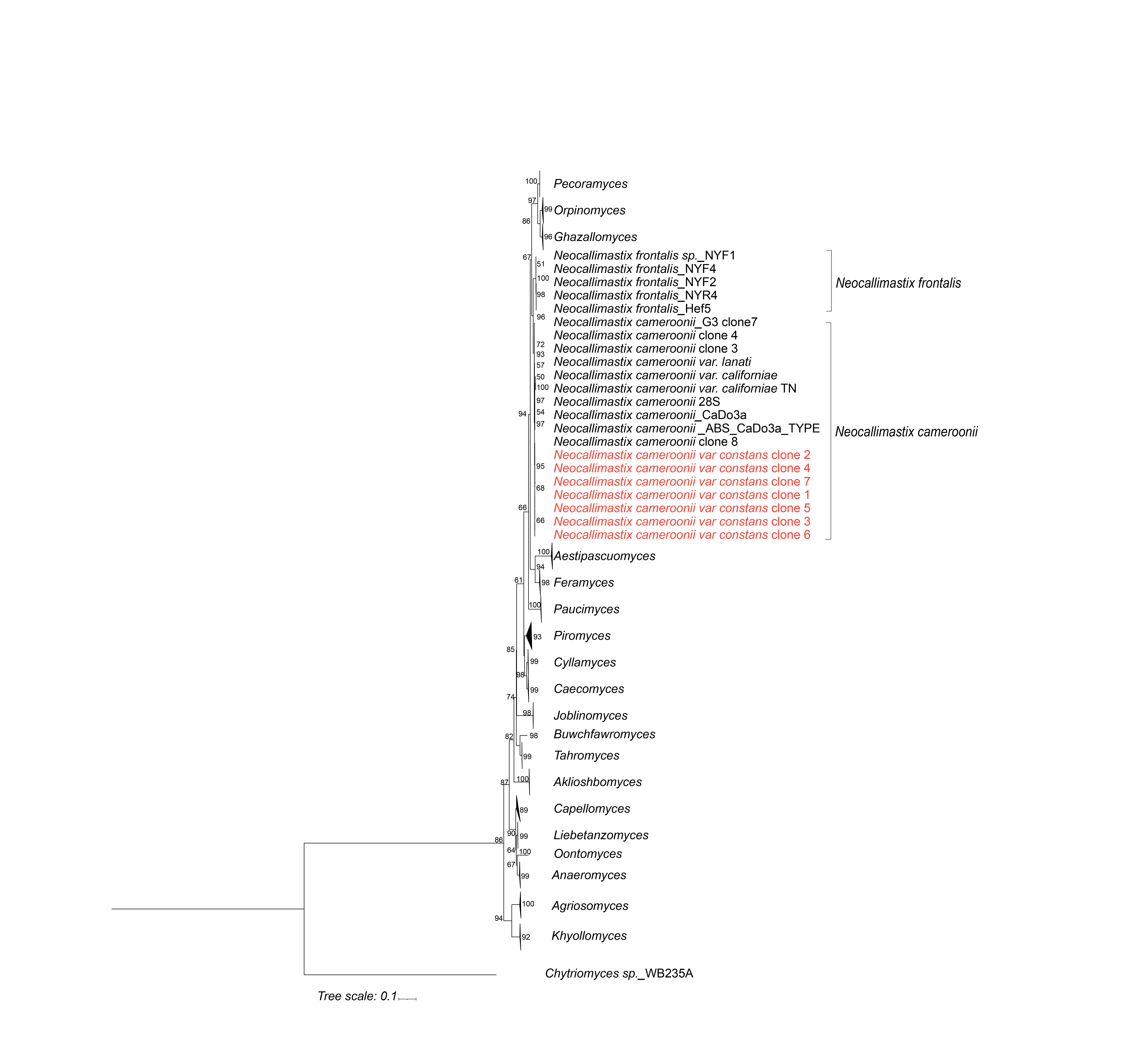
**

**Figure S1**. Phylogenetic tree made with D1/D2 Large Ribosomal Subunit (LSU) sequences. Sequences and accession numbers are listed in Table S1. *Neocallimastix cameroonii* var. constans is placed within the *Neocallimastix* genus and is closely related to other *camerooni* strains.


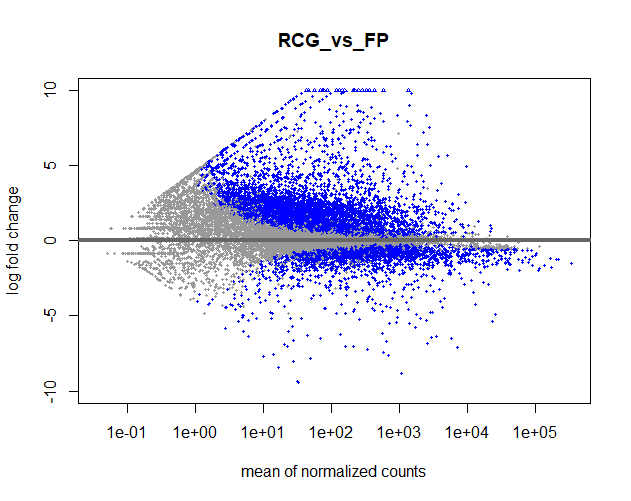

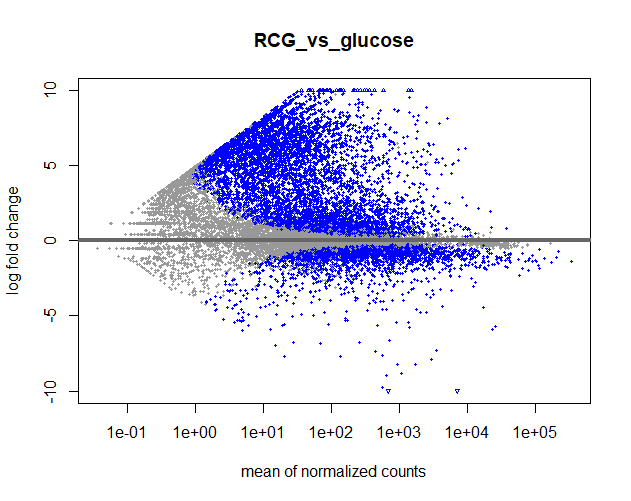


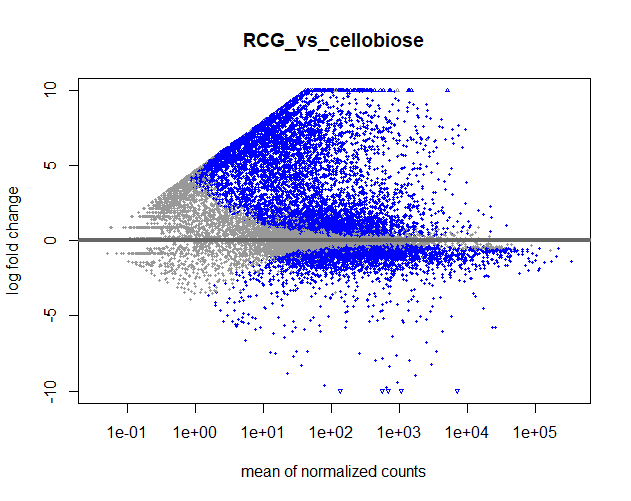

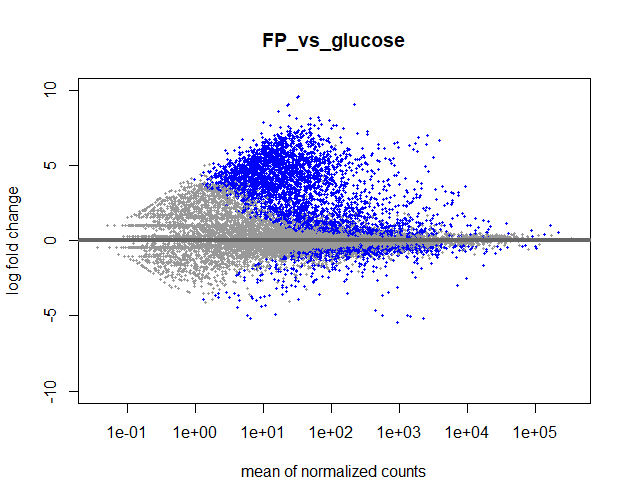


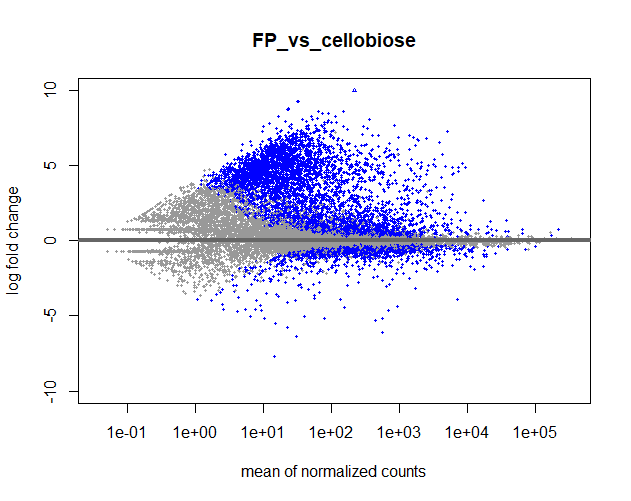

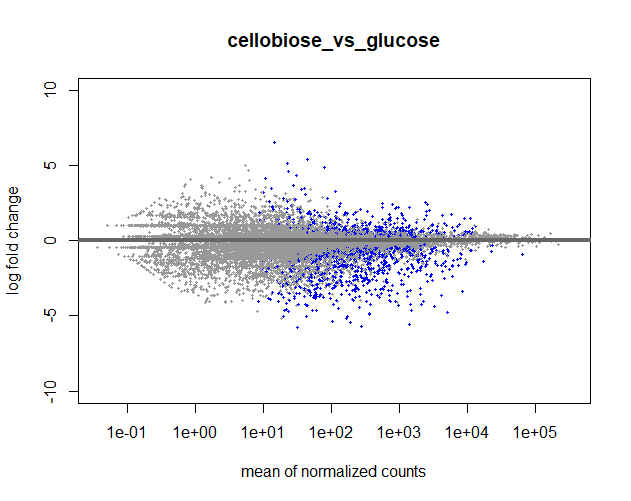


**Figure S2**. Plots show DESeq2 pairwise substrate comparisons; blue dots are significantly differentially expressed (padj<0.05). RCG stands for reed canary grass; FP stands for filter paper. Figures were made with DESeq2 plotMA command. No TPM cutoff was applied for these figures.


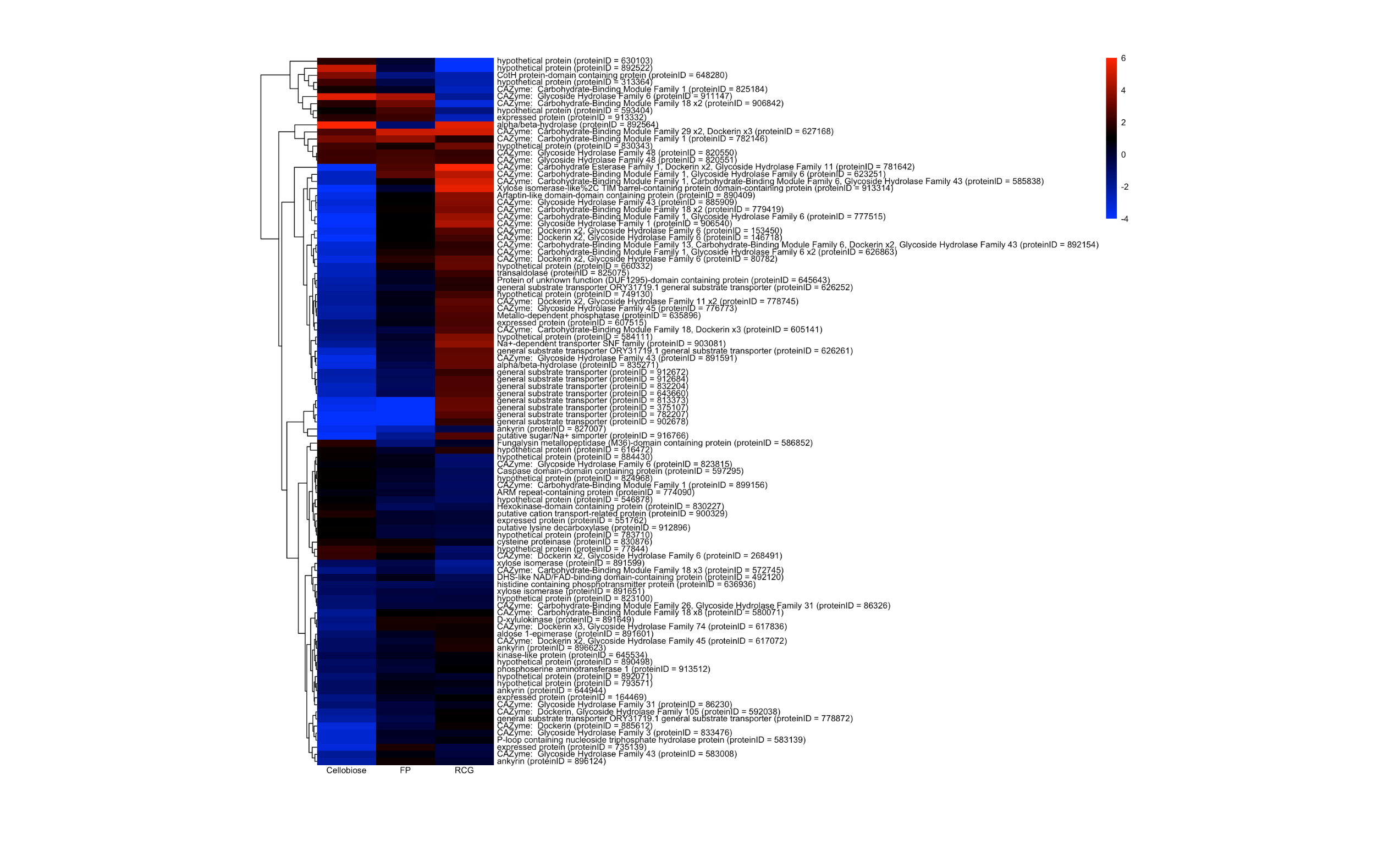


**Figure S3.** A heatmap of 100 predicted genes with the lowest p-value taken from the condition of cellobiose compared to glucose. Each row represents the log2 fold change value of a gene for each column’s substrate with respect to glucose.


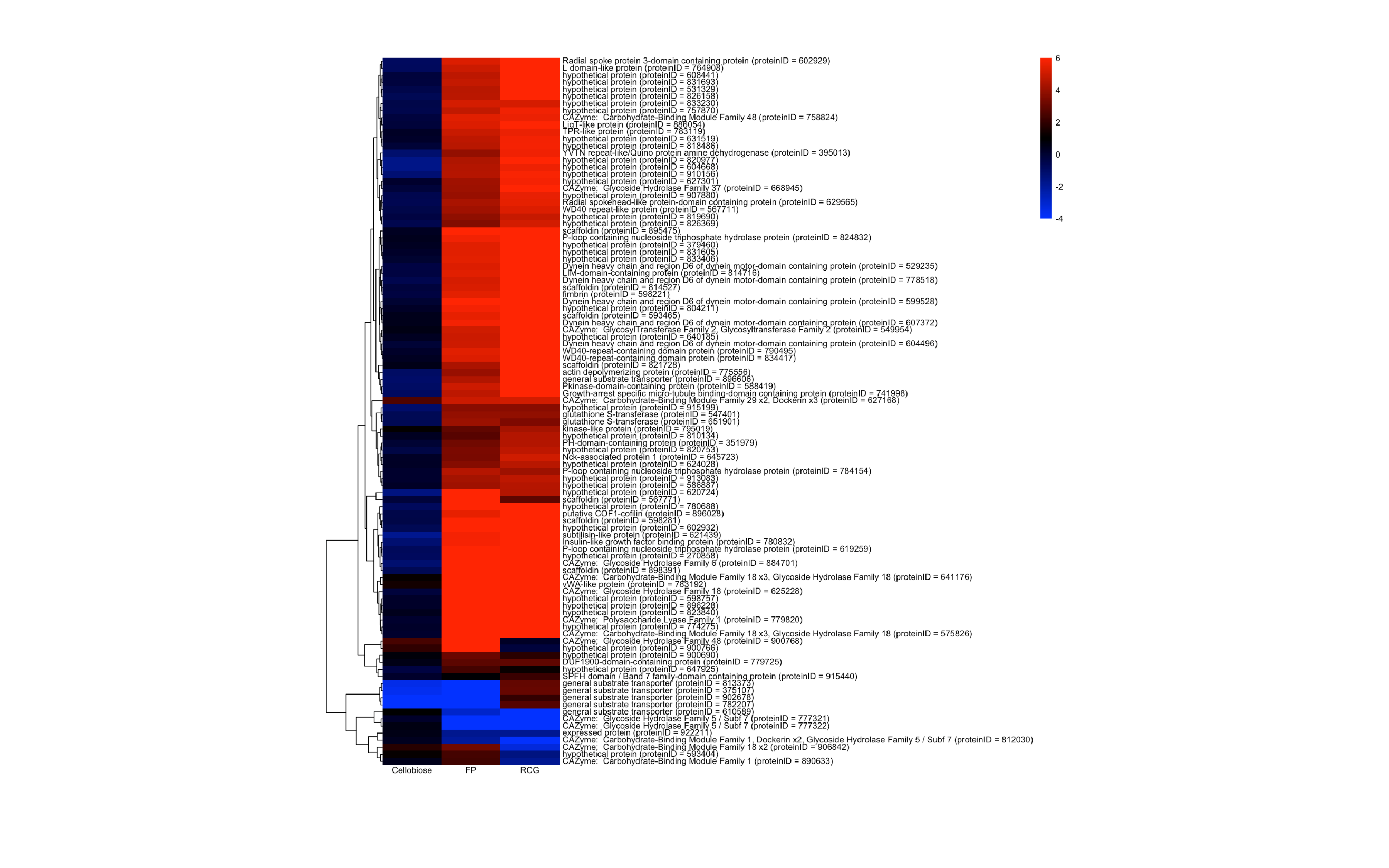


**Figure S4.** A heatmap of 100 predicted genes with the lowest p-value taken from the condition of filter paper (FP) compared to glucose. Each row represents the log2 fold change value of a gene for each column’s substrate with respect to glucose.


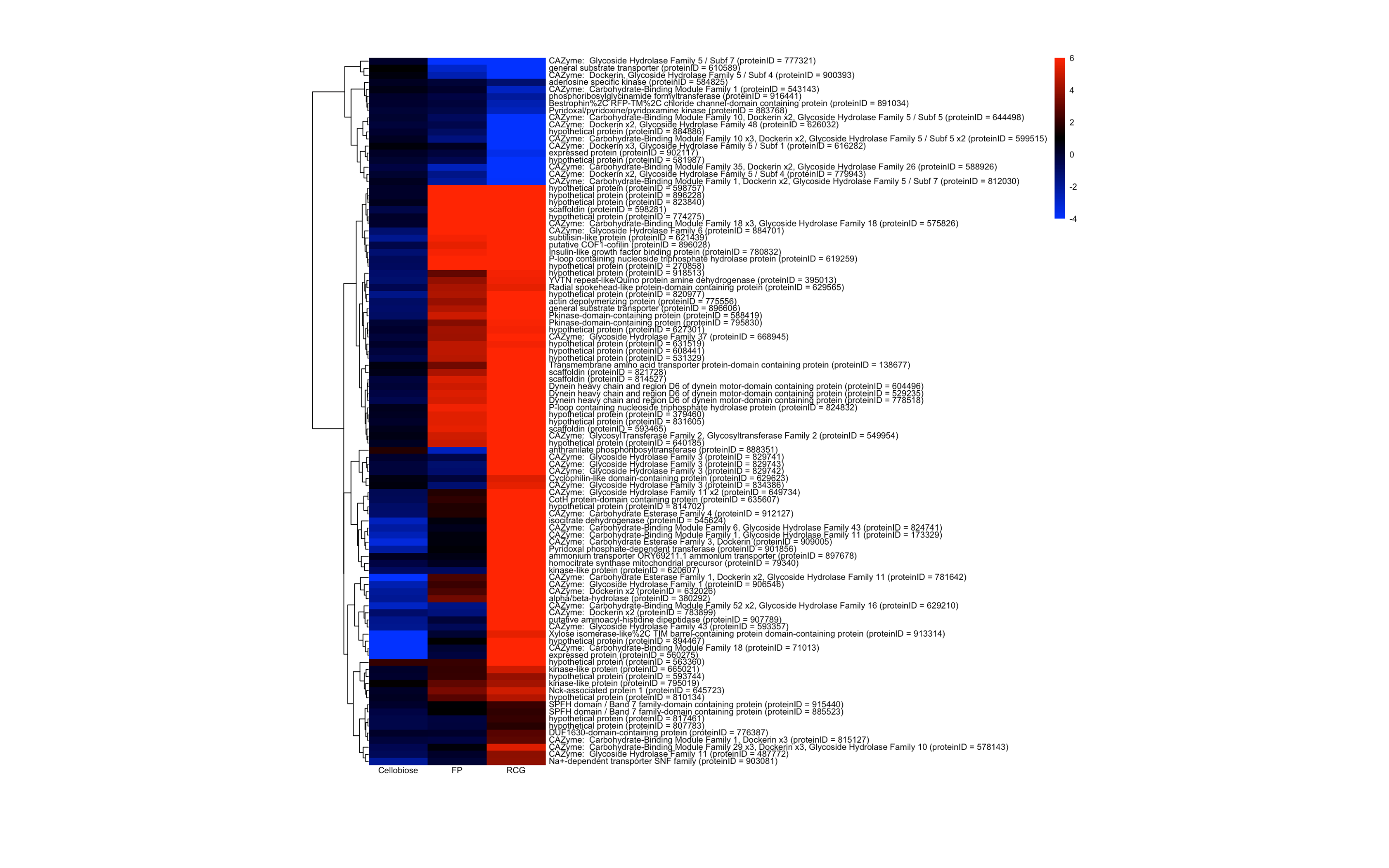


**Figure S5.** A heatmap of 100 predicted genes with the lowest p-value taken from the condition of reed canary grass (RCG) compared to glucose. Each row represents the log2 fold change value of a gene for each column’s substrate with respect to glucose.


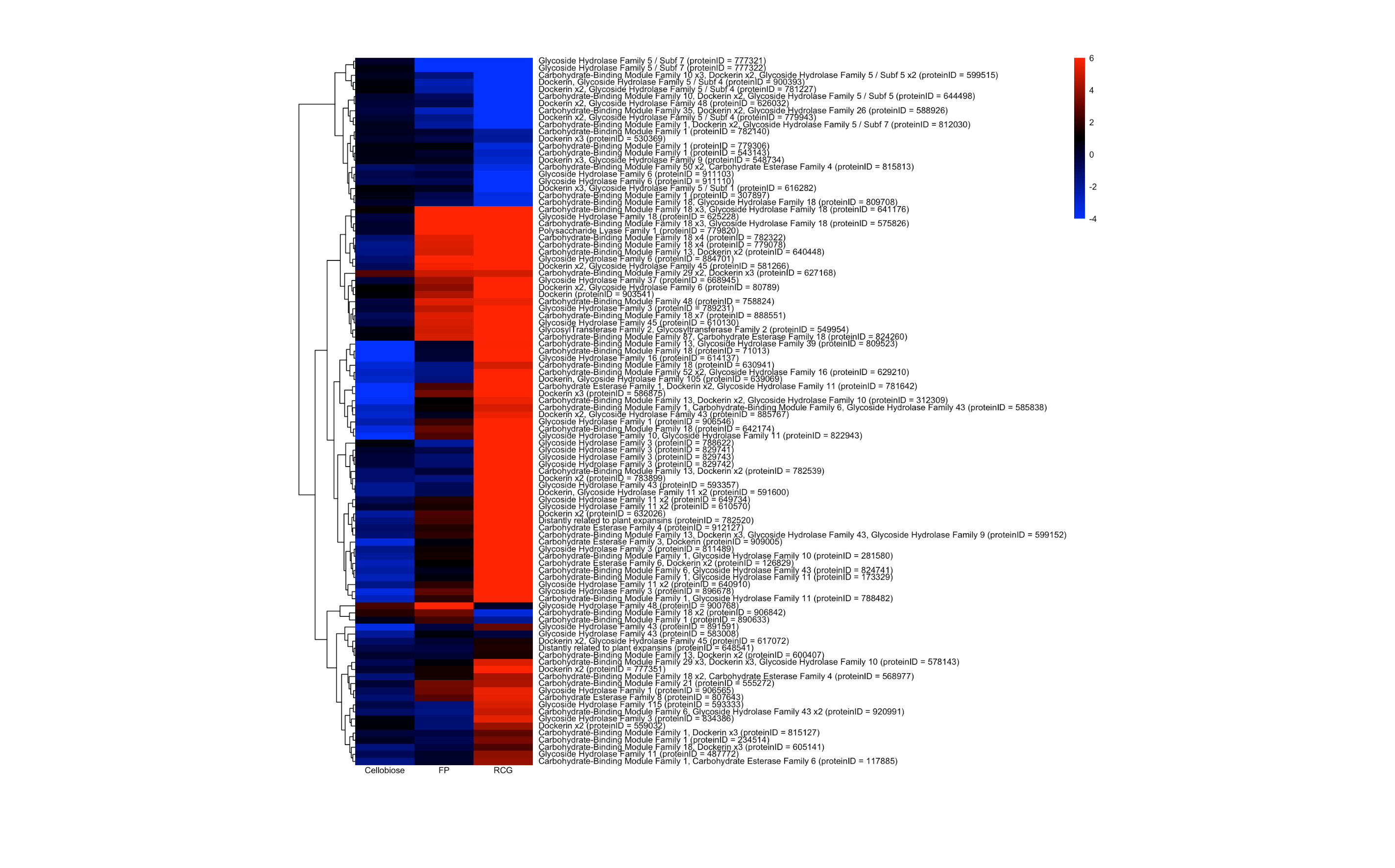
**Figure S6.** A heatmap of 100 predicted CAZymes with the lowest minimum p-values corresponding to the log2 fold change value of a predicted CAZyme for each condition. Each row represents the log2 fold change value of a CAZyme for each column’s substrate with respect to glucose.


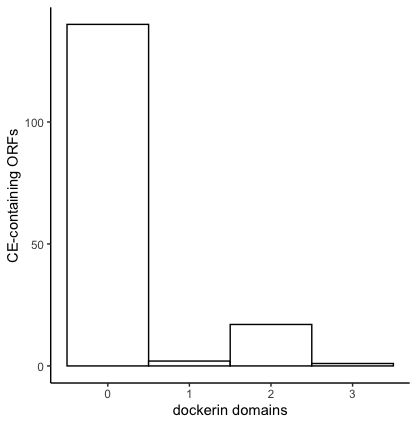

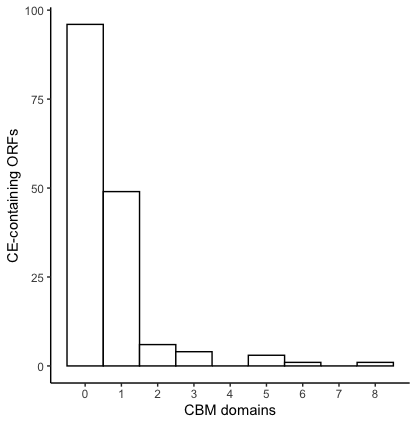

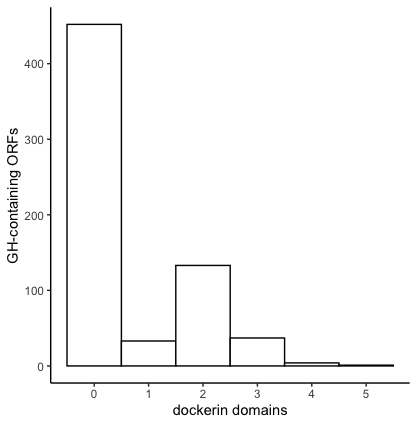

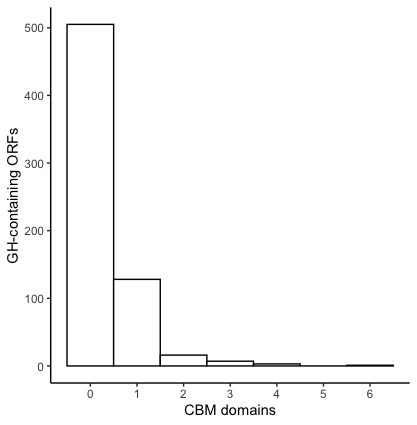


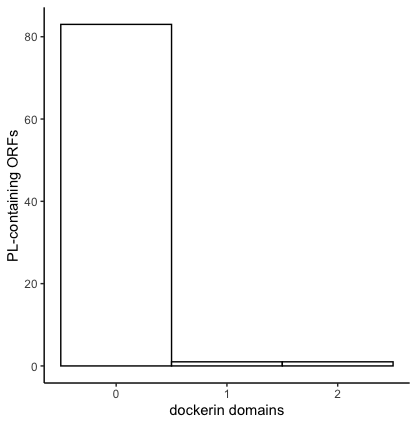

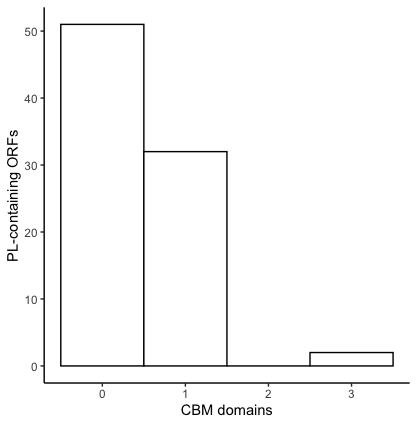


**Figure S7.** Distributions of dockerin domains and carbohydrate-binding modules (CBM) for 3 classes of CAZymes: cellulose esterases (CE), glycoside hydrolases (GH), and polysaccharide lyases (PLs). Glycosyl transferase (GT)-containing ORFs lacked annotations for DOC2 or CBM, corresponding to fungal dockerin sequences or carbohydrate-binding modules respectively (Cantarel et al. 2009).


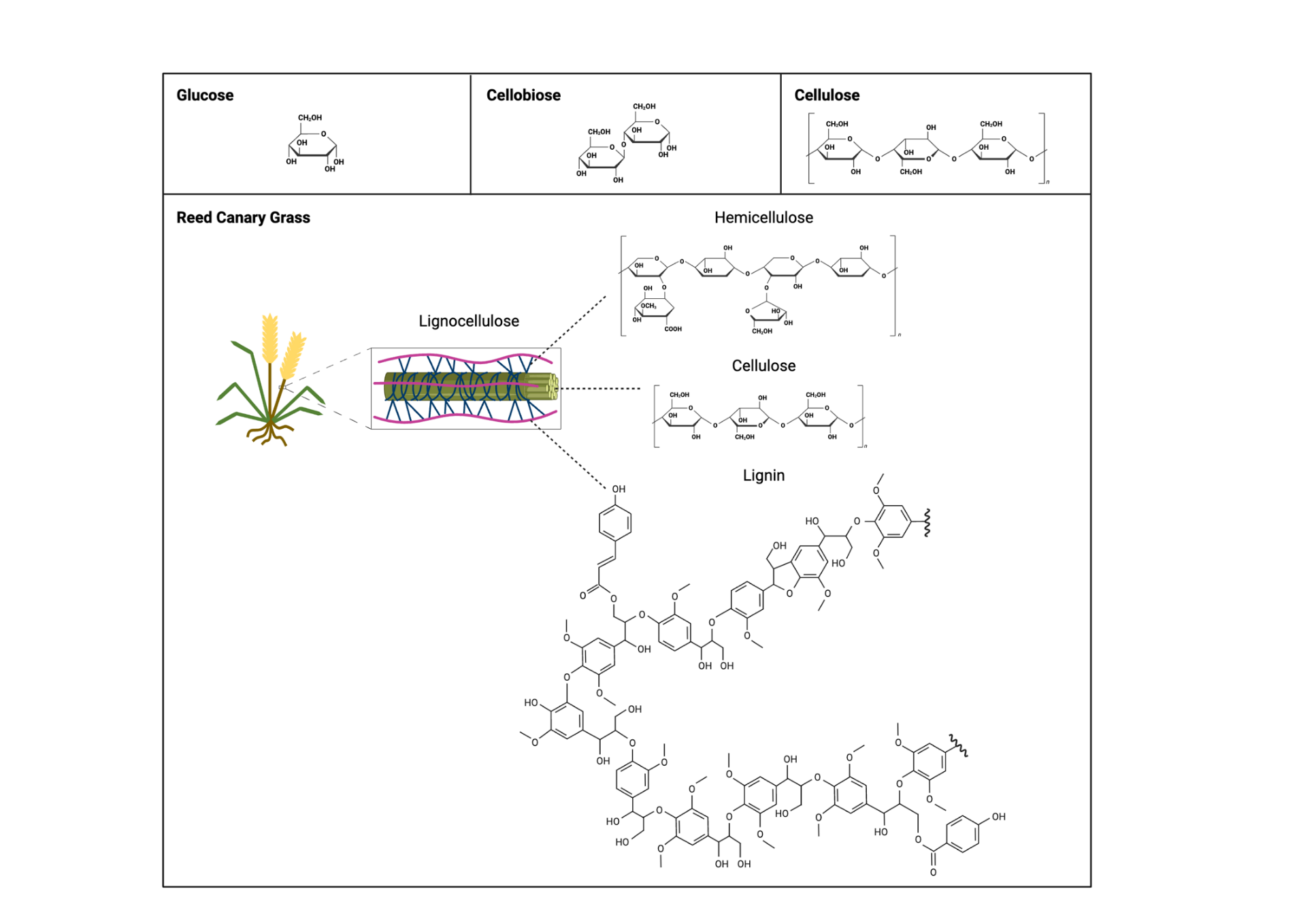


**Figure S8.** Structures of substrates used in *N. cameroonii* var. constans cultures: glucose, cellobiose, cellulose, and lignocellulose. Filter paper is comprised of cellulose, and reed canary grass is comprised of lignocellulose. Parts of the figure were created with BioRender.com. Lignin structure was adapted from (Bertella and Luterbacher 2020).

**Figure S9**. Total counts of differentially expressed genes for pairwise substrate comparisons of *N. cameroonii* var constans grown on reed canary grass, glucose, filter paper, or cellobiose. Significance is set to padj<0.05, and TPM cutoff is set to genes having an average TPM greater than 2 for one or both conditions compared.

**Figure S10.** Methane production of methanogens when co-cultured with *N. californiae* and *N. cameroonii* var. constans shows ability of anaerobic fungi to degrade lignocellulose into fermentation products that methanogens can convert to methane. Note that methane was not detected in fungal monocultures. Error bars represent standard deviation of two biological replicates, and these data represent a separate dataset from the results presented in Figure 7.

**Figure S11**. *N. cameroonii* var. constans is grown in monoculture on MC– media with different carbon substrates; RCG is reed canary grass, and FP is filter paper; controls are uninoculated MC– media with the indicated carbon substrate. These carbon substrates were used when the fungal monoculture was grown for RNA extractions. These data show that cultures are in the exponential phase for some time, including day 2 and day 3, which is when the fungal monocultures were harvested for RNA extraction.

**Table S1.** D1-D2 LSU sequences used to build phylogenetic tree with GenBank accession codes (ID) if available.

| Isolate | ID | Sequence |
| --- | --- | --- |
| Chytriomyces_sp._WB235A_isolate | DQ536493.1 | ACGGCGAGTGAAGCGGGAAAAGCTCAAATTTGAAATCTGGCAGGTTCCCCTGTCCGAATTGTAGTTTCAAGGTGCGTTTTCGGGGGCGGCTTAGGTCCAAGTCTGTTGGAATGCAGCGCCGTAGAGGGTGAGAGCCCCGTAGGGCCTGACGTTCGCTCCTGTATGATACGTATCCGAAGAGTCGCATAGTTTGAGAATGCTGTGCAAATTGGGTGGTAAATATCATCTAAAGCTAAATACTGGCGAGAGACCGATAGCGAACAAGTACCGTGAGGGAAAGATGAAAAGAACTTTGAAAAGAGAGTTAAAAGTACGTGAAATTGTCAAAAGGGAAACGCTTGAAACCAGTATCGGGACTAGATTATCAACCGCAAGGTGCATTAGTCTAGTTCTGGGTTAGCATCAGTTTGGTTTGGAGTAAAAAGCGAGTGGAAGGTAGCCTTTCGAGGTGTTATAGCCGCTCGTCATGCTCTGGACTGGACTGAGGACCGCAGGTATTTTGGGGAACTTGCCGCCATGGAAAGCCCATGTATAAACAACTTGTTGATTATGCGTGTGCCCAAGTGCACGGTAGGGTACCACTTCTACCTTAGGATGCTGA |
| Joblinomyces_apicalis_isolate_GFH683_Clone3 | MT085672 | AACTTGATCTCAAATCAGATAAGAGTACCCGCTGAACTTAAGCATATCAATAAGCGGAGGAAAAGAAACTAACAAGGATTCCCCTAGTAACGGCGAGTGAAGCGGGAAGAGCTCAAATTTGAAATCTTCAAGGTTTGACCTTGACGAATTGTAGTTTAAAGAAGTGTTTTCGGTTAGAGGAGTGGTAAAAGTTCTTTGGAGTAGGACATCATAGAGGGTGAGAATCCCGTATTTGACCATTCTTTCTAGCTTTGTGATACACTTTCAAAGAGTCGGATTGTTTGGGAATGCAGTCCAAAATGGGTGGTAAATTTCATCTAAAGCTAAATATTGGCGAGAGACCGATAGCGAACAAGTACCGTGAGGGAAAGATGAAAAGAACTTTGAAAAGAGAGTTAAACAGTACGTGAAATTGTCAAAAGGGAAGCGTTTGACACCAGTGTTGTTTTTCCGAAAATCAATTAAAAATGAAATCATGCTAGGTTGTATTGACCTTATCAGCTTGCAATTTAGTTTTGGTTTCCTTTTTGATGCACTTTTCGGTTAACAAGTCAACATCAATTTCCTTTGCTGTAAAAAGACGAGAGGAAGGTGGCTTCAATTTCGGTTGAAGTGTTTATAGCCTTTCGCCTCTGCAGTCGGGGAGATTGAGGTCTGCAGCGTAGATCCTTCGGGGCTAGATACAGTCGTGCTAAGTTATAGCCAGTTATAGACAACTTGTTGACTATGATTGGTCTTGTAGCTTACTCGCGGTTGCTATTTTACCTACGCTTAGGATGTTGA |
| Joblinomyces_apicalis_isolate_GFH683_Clone2 | MT085671 | AACTTGATCTCAAATCAGATAAGAGTACCCGCTGAACTTAAGCATATCAATAAGCGGAGGAAAAGAAACTAACAAGGATTCCCCTAGTAACGGCGAGTGAAGCGGGAAGAGCTCAAATTTGAAATCTTCAAGGTTTGACCTTGACGAATTGTAGTTTAAAGAAGTGTTTTCGGTTAGAGGAGTGGTAAAAGTTCTTTGGAATAGGACATCATAGAGGGTGAGAATCCCGTATTTGACCATTCTTTCTAGCTTTGTGATACACTTTCAAAGAGTCGGATTGTTTGGGAATGCAGTCCAAAATGGGTGGTAAATTTCATCTAAAGCTAAATATTGGCGAGAGACCGATAGCGAACAAGTACCGTGAGGGAAAGATGAAAAGAACTTTGAAAAGAGAGTTAAACAGTACGTGAAATTGTCAAAAGGGAAGCGTTTGACACCAGTGTTGTTTTTCCGAAAATCAATTAAAAATGAAATCATGCTAGGTTGTATTGACCTTATCAGCTTGCAATTTGGTTTTGGTTTCCTTTTTGATGCACTTTTCGGTTAACAAGTCAACATCAATTTCCTTTGCTGTAAAAAGACGAGAGGAAGGTGGCTTCAATTTCGGTTGAAGTGTTTATAGCCTTTCGCCTCTGCAGTCGGGGAGATTGAGGTCTGCAGCGTAGATCCTTCGGGGCTAGATACAGTCGTGCTAAGTTATAGCCAGTTATAGACAACTTGTTGACTATGATTGGTCTTGTAGCTTACTCGCGGTTGCTATTTTACCTACGCTTAGGATGTTGA |
| Aklioshbomyces_papillarum_WT2_Clone_9 | MT085739 | AACTTGATCTCAAATCAGATAAGAGTACCCGCTGAACTTAAGCATATCAATAAGCGGAGGAAAAGAAACTAACAAGGATTCCCTTAGTAACGGCGAGTGAAGCGGGAAGAGCTCAAATTTGAAATCTTCAAGGTTTGACCTTGACGAATTGTAGTTTATAGAAGTGTTTTCTGTTGGAGTTTTGGTAAAAGTTCTTTGGAATAGGACATCATAGAGGGTGAGAATCCCGTATTTGACCATGACTTTCCAGCTGTGTGATACACTTTCAAAGAGTCGGATTGTTTGGGAATGCAGTCCAAAATGGGTGGTAAATTTCATCTAAAGCTAAATATTGGCGAGAGACCGATAGCGAACAAGTACCGTGAGGGAAAGATGAAAAGAACTTTGAAAAGAGAGTTAAACAGTACGTGAAATTGTCAAAAGGGAAGCGTTTGACACCAGTGTTGTTTTCTCGAAAATCAATTTAATGAGTTCTTGTTTTTAAGTTATTGTGACTTTATCAGCGCATTTCTTATTTTACTTGACTCATTGAATGCACTTTTCGTTTAACAAGTCAACATCAATTTCTTTGGTGGTAAAAAGTTTAAAGGAAGGTGGCTTGGACTTCGGTTCAAGTGTTTATAGCCTTTTTTCTATGCCATCGGAGAGATTGAGGTCTGCAGCGTAGATCCTTCGGGGTTAGATACCTTTGTGCTAAGTTATAGTTTTTTATAGACAACTTGTTGACTATAATTAACCCTGTAATTTACTCGCTTAGGCTATTTTACCTACGCTTAGGATGTTGA |
| Aklioshbomyces_papillarum_WT2_Clone_10 | MT085740 | AACTTGATCTCAAATCAGATAAGAGTACCCGCTGAACTTAAGCATATCAATAAGCGGAGGAAAAGAAACTAACAAGGATTCCCTTAGTAACGGCGAGTGAAGCGGGAAGAGCTCAAATTTGAAATCTTCAAGGTTTGACCTTGACGAATTGTAGTTTATAGAAGTGTTTTCTGTTGGAGTTTTGGTAAAAGTTCTTTGGAATAGGACATCATAGAGGGTGAGAATCCCGTATTTGACCATGACTTTCCAGCTGTGTGATACACTTTCAAAGAGTCGGATTGTTTGGGAATGCAGTCCAAAATGGGTGGTAAATTTCATCTAAAGCTAAATATTGGCGAGAGACCGATAGCGAACAAGTACCGTGAGGGAAAGATGAAAAGAACTTTGAAAAGAGAGTTAAACAGTACGTGAAATTGTCAAAAGGGAAGCGTTTGACACCAGTGTTGTTTTCTCGAAAATCAATTTAATGAGTTCTTGTTTTTAAGTTATTGTGACTTTATCAGCGCATTTCTTATTTTACTTGACTCATTGAATGCACTTTTCGTTTAACAAGTCAACATCAATTTCTTTGGTGGTAAAAAGTTTAAAGGAAGGTGGCTTGGACCTCGGCTCAAGTGTTTATAGCCTTTTTTCTATGCCATCGGAGAGATTGAGGTCTGCAGCGTAGATCCTTCGGGGTTAGATACCTTTGTGCTAAGTTATAGTTTTTTATAGACAACTTGTTGACTATAATTAACCCTGTAATTTACTCGCTTAGGCTATTTTACCTACGCTTAGGATGTTGA |
| Aklioshbomyces_papillarum_WT2_Clone_7 | MT085737 | AACTTGATCTCAAATCAGATAAGAGTACCCGCTGAACTTAAGCATATCAATAAGCGGAGGAAAAAGAAACTAACAAGGATTCCCTTAGTAACGGCGAGTGAAGCGGGAAGAGCTCAAATTTGAAATCTTCAAGGTTTGACCTTGACGAATTGTAGTTTATAGAAGTGTTTTCTGTTGGAGTTTTGGTAAAAGTTCTTTGGAATAGGACATCATAGAGGGTGAGAATCCCGTATTTGACCATGACTTTCCAGCTGTGTGATACACTTTCAAAGAGTCGGATTGTTTGGGAATGCAGTCCAAAATGGGTGGTAAATTTCATCTAAAGCTAAATATTGGCGAGAGACCGATAGCGAACAAGTACCGTGAGGGAAAGATGAAAAGAACTTTGAAAAGAGAGTTAAACAGTACGTGAAATTGTCAAAAGGGAAGCGTTTGACACCAGTGTTGTTTTCTCGAAAATCAATTTAATGAGTTCTTGTTTTTAAGTTATTGTGACTTTATCAGCGCATTTCTTATTTTACTTGACTCATTGAATGCACTTTTCGTTTAACAAGTCAACATCAATTTCTTTGGTGGTAAAAAGTTTAAAGGAAGGTGGCTTGGACCTCGGCTCAAGTGTTTATAGCCTTTTTTCTATGCCATCGGAGAGATTGAGGTCTGCAGCGTAGATCCTTCGGGGTTAGATACCTTTGTGCTAAGTTATAGTTTTTTATAGACAACTTGTTGACTATAATTAACCCTGTAATTTACTCGCTTAGGCTATTTTACCTACGCTTAGGATGTTGA |
| Oontomyces_anksri_ABS_SSDCIB1_28S_rRNA_gene_partial_sequence_from_TYPE_material | NG_67779.1 | ATCCCCCGAGAAAGAACTAACAAGGATTCCCCTAGTAACGGCGAGTGAAGCGGGAAGAGCTCAAATTTGAAATCTTAAAGGATTGTCCTTTACGAATTGTAGTTTATAGAAGTGTTTTCTGTTGAGGTTTCGGTAAAAGTTCTTTGGAATAGGACATCATAGAGGGTGAGAATCCCGTATTTGACCGTTACTTCTCAGCTGTGTGATACACTTTCAACGAGTCGGATTGTTTGGGAATGCAGTCCAAAATGGGTGGTAAATTTCATCTAAAGCTAAATATTGGCGAGAGACCGATAGCGAACAAGTACCGTGAGGGAAAGATGAAAAGAACTTTGAAAAGAGAGTTAAACAGTACGTGAAATTGTCAAAAGGGAAGCGTTTGACACCAGTGTTGTTTTCTCGAAAATCAATTCAATAGTTTTTAATTCTGAGTAGTCTTGACTTTATCAGCTTGCTGCTCTCTTTTTGGAAATTATTGAATGCACTTTTCGTTTAACAAGTCAACATCAATTTCTTTTGTTGTAAAAGAGTAAGAGGAAGGTGGCTTACGTTTCGGCGTAAGTGTTTATAGCCTTTTACTTCTGCAATCGGAGAGATTGAGGTCTGCAGCGTAGATCCTTCGGGGTTAGATGCATTTGTGCTAAGTTATAGCCTTGTATAGACAACTTGTTGACTATACTCAGTCATGTAGCTTACTCGCTTATGTCATTTTACCTACGCTTAGGATGTTGA |
| Anaeromyces_contortus_isolate_G3G_Clone_8 | MG605686.1 | AACTTGATCTCAAATCAGATAAGAGTACCCGCTGAACTTAAGCATATCAATAAGCGGAGGAAAAGAAACTAACAAGGATTCCCCTAGTAACGGCGAGTGAAGCGGGAAGAGCTCAAATTTGAAATCTTAAAGGGTTTCCCTTTACGAATTGTAGTTTAAAGAAGTGTTTTCTGTTGGAGTTTCGGTAAAAGTTCTTTGGAATAGGACATCATAGAGGGTGAGAATCCCGTATTTGACCGTTACTTTCCAGCTGTGTGATACACTTTCAAAGAGTCGGATTGTTTGGGAATGCAGTCCAAAATGGGTGGTAAATTTCATCTAAAGCTAAATATTGGCGAGAGACCGATAGCGAACAAGTACCGTGAGGGAAAGATGAAAAGAACTTTGAAAAGAGAGTTAAACAGTACGTGAAATTGTCAAAAGGGAAGCGTTTGACACCAGTGTTGTTTAGTTGAAAATCAATTCAATAATTTTTGATTTTGAGTAGTATTGACTTCATCAGCTTGCTGCTTTCTTTTCAGAAATTATTGAATGCACTTTTCAGTTAACAAGTCAACATCAATTTCTTTTGTTGTAAAAGGGTAAGAGGAAGGTGGCTTACGCTTCAGCGTAAGTGTTTATAGCCTTTTACCTCTGCAATCGGAGAGATTGAGGACAGCAGCGTAGACTCTTCGGGGTTAGATACACTTGTGCTAAGCTATAGCCTTGTATAGACAACTTGTTGACTATGCTCAGTCATGTAGCTTACTCGCTTGTGCTATTTTACCTACGCTTAGGATGTTGA |
| Anaeromyces_contortus_isolate_G3C_Clone_6 | MG605683.1 | AACTTGATCTCAAATCAGATAAGAGTACCCGCTGAACTTAAGCATATCAATAAGCGGAGGAAAAGAAACTAACAAGGATTCCCCTAGTAACGGCGAGTGAAGCGGGAAGAGCTCAAATTTGAAATCTTAAAGGGTTTCCCTTTACGAATTGTAGTTTAAAGAAGTGTTTTCTGTTAGAGTTTCGGTAAAAGTTCTTTGGAATAGGACATCATAGTGGGTGAGAATCCCGTATTTGACCGTTACTTTCCAGCTGTGTGATACACTTTCAAAGAGTCGGATTGTTTGGGAATGCAGTCCAAAATGGGTGGTAAATTTCATCTAAAGCTAAATATTGGCGAGAGACCGATAGCGAACAAGTACCGTGAGGGAAAGATGAAAAGAACTTTGAAAAGAGAGTTGAACAGTACGTGAAATTGTCAAAAGGGAAGCGTTTGACACCAGTGTTGTTTAGTTGAAAATCAATTCAATAATTTTTGATTTTGAGTAGTATTGACTTCATCAGCTTGCTGCTTTCTTTTCAGAAATTATTGAATGCACTTTTCAGTTAACAAGTCAACATCAATTTCTTTTGTTGTAAAAGGGTAAGAGGAAGGTGGCTTACGCTTCGGCGTAAGTGTTTATAGCCTTTTACCTCTGCAATCGGAGAGATTGAGGACAGCAGCGTAGACTCTTCGGGGTTAGATACACTTGTGCTAAGCTATAGCCTTGTATAGACAACTTGTTGACTATGTTCAGTCATGTAGCTTACTCGCTTGTGCTATTTTACCTACGCTTAGGATGTTGA |
| Capellamyces_elongis_isolate_GFKJa1916 | MT085701.1 | AACTTGATCTCAAATCAGATAAGAGTACCCGCTGAACTTAAGCATATCACTATGCGGAGGAAAAGAAACTATCACGGATTCCCCTAGTAACGGCGAGTGAAGCGGGAAGAGCTCAAATTTGAAATCTTAAAGGGTTTCCCTTTACGAATTGTAGTTTAAAGAAGTGTTTTCTGTTGGAGTTTCGGTAAAAGTTCTTTGGAATAGGACATCATAGAGGGTGAGAATCCCGTATTTGACCGTTACTTTCCAGCTGTGTGATACACTTTCAAAGAGTCGGATTGTTTGGGAATGCAGTCCAAAATGGGTGGTAAATTTCATCTAAAGCTAAATATTGGCGAGAGACCGATAGCGAACACGTACCGTGAGGGAAAGATGAAAAGAACTTTGAAAAGAGAGTTAAACACTACGTGAAATTGTCAAAAGGGAAGCGTTTGACACCACTGTTGTTTAGTTGAAAATCACTTTAATAATTTTTGATTTTAAGTAGTGTATTGACTTTATCAGCTTGCTGCTTTCTTTTCGAAAATTATTGAATGCACTTTTCAGTTAACAAGTCAACATCAATTTCTTTTGTTGTAAAAGGGTAAGAGGAAGGTGGCTTGCGCTTCGGCTCAAGTGTTTATAGCCTTTTACCTCTGCAATCGGAGAGATTGAGGTCTGCAGCGTAGATCCTTCGGGGTTAGATACATTTGTGCTAAGCTATAGCCTTGTATAGACAACTTGTTGACTATGCTCAGTCATGTAGCTTACTCGCTTATGCTATTTACCTACGCTAGGATGTGAC |
| Capellomyces_foraminis_isolate_BGB11_Clone_C3 | MT085698 | AACTTGATCTCAAATCAGATAAGAGTACCCGCTGAACTTAAGCATATCAATAAGCGGAGGAAAAGAAACTAACAAGGATTCCCCTAGTAACGGCGAGTGAAGCGGGAAGAGCTCAAATTTGAAATCTTAAAGGGTTTCCCTTTACGAATTGTAGTTTAAAGAAGTGTTTTCTGTTGGAGTTTCGGTAAAAGTTCTTTGGAATAGGACATCATAGAGGGTGAGAATCCCGTATTTGACCGTTACTTTCCAGCTGTGTGATACACTTTCAAAGAGTCGGATTGTTTGGGAATGCAGTCCAAAATGGGTGGTAAATTTCATCTAAAGCTAAATATTGGCGAGAGACCGATAGCGAACAAGTACCGTGAGGGAAAGATGAAAAGAACTTTGAAAAGAGAGTTAAACAGTACGTGAAATTGTCAAAAGGGAAGCGTTTGACACCAGTGTTGTTTAGTTGAAAATCAATTTAATAATTTTTGATTCTAAGTAGTATTGACTTTATCAGCTTGCTGCTTTCTTTTCGAAAATTATTGAATGCACTTTTCAGTTAACAAGTCAACATCAATTTCTTTTGTTGTAAAAAGGTAAGAGGAAGGTGGCTTGCGCTTCGGCTCAAGTGTTTATAGCCTTTTACCTCTGCAATCGGAGAGATTGAGGTCTGCAGCGTAGATCCTTCGGGGTTAGATACATTTGTGCTAAGCTATAGCCTTGTATAGACAACTTGTTGACTATGCTCAGTCATGTAGCTTACTCGCTTATGCTATTTTACCTACGCTTAGGATGTTAA |
| Capellomyces_foraminis_isolate_BGB11_Clone_C2 | MT085697 | AACTTGATCTCAAATCAGATAAGAGTACCCGCTGAACTTAAGCATATCAATAAGCGGAGGAAAAGAAACTAACAAGGATTCCCCTAGTAACGGCGAGTGAAGCGGGAAGAGCTCAAATTTGAAATCTTAAAGGGTTTCCCTTTACGAATTGTAGTTTAAAGAAGTGTTTTCTGTTGGAGTTTCGGTAAAAGTTCTTTGGAATAGGACATCATAGAGGGTGAGAATCCCGTATTTGACCGTTACTTTCCAGCTGTGTGATACACTTTCAAAGAGTCGGATTGTTTGGGAATGCAGTCCAAAATGGGTGGTAAATTTCATCTAAAGCTAAATATTGGCGAGAGACCGATAGCGAACAAGTACCGTGAGGGAAAGATGAAAAGAACTTTGAAAAGAGAGTTAAACAGTACGTGAAATTGTCAAAAGGGAAGCGTTTGACACCAGTGTTGTTTAGTTGAAAATCAATTTAATAATTTTTGATTCTAAGTAGTATTGACTTTATCAGCTTGCTGCTTTCTTTTCGAAAATTATTGAATGCACTTTTCAGTTAACAAGTCAACATCAATTTCTTTTGTTGTAAAAAGGTAAGAGGAAGGTGGCTTGCGCTTCGGCTCAAGTGTTTATAGCCTTTTACCTCTGCAATCGGAGAGATTGAGGTCTGCAGCGTAGATCCTTCGGGGTTAGATACATTTGTGCTAAGCTATAGCCTTGTATAGACAACTTGTTGACTATGCTCAGTCATGTAGCTTACTCGCTTATGCTATTTTACCTACGCTTAGGATGTTGA |
| Liebetanzomyces_isolate_Cel1A_Clone_3 | MT085727 | AACTTGATCTCAAATCAGATAAGAGTACCCGCTGAACTTAAGCATATCAATAAGCGGAGGAAAAGAAACTAACAAGGATTCCCCTAGTAACGGCGAGTGAAGCGGGAAGAGCTCAAATTTGAAATCTTAAAGGGTTTCCCTTTACGAATTGTAGTTTAAAGAAGTGTTTTCTGTTGGAGTTTCGGTAAAAGTTCTTTGGAATAGGACATCATAGAGGGTGAGAATCCCGTATTTGACCGTTACTTTCCAGCTGTGTGATACACTTTCAAAGAGTCGGATTGTTTGGGAATGCAGTCCAAAATGGGTGGTAAATTTCATCTAAAGCTAAATATTGGCGAGAGACCGATAGCGAACAAGTACCGTGAGGGAAAGATGAAAAGAACTTTGAAAAGAGAGTTAAACAGTACGTGAAATTGTCAAAAGGGAAGCGTTTGACACCAGTGTTGTTTAGTTGAAAATCAATTCAATAGTTTTTGATTCTAAGTAGTATTGACTTCATCAGCTTGCTGCTTTCTTCTCAGAAATTATTGAATGCACTTTTCAGTTAACAAGTCAACATCAATTTCTTCTGTTGTAAAAGGGTAAGAGGAAGGTGGCTTGCGCTTCGGCTCAAGTGTTTATAGCCTTTTATCTCTGCAATCGGAGAGATTGAGGTCTGCAGCGTAGATCCTTCGGGGTTAGATACATTGGTGCTAAGCTATAGCCTTGTATAGACAACTTGTTGACTATGCTCAGTCATGTAGCTTACTCGCTGATGCTATTTTACCTACGCTTAGGATGTTGA |
| Liebetanzomyces_isolate_Cel1A_Clone_2 | MT085726 | AACTTGATCTCAAATCAGATAAGAGTACCCGCTGAACTTAAGCATATCAATAAGCGGAGGAAAAGAAACTAACAAGGATTCCCCTAGTAACGGCGAGTGAAGCGGGAAGAGCTCAAATTTGAGATCTTAAAGGGTTTCCCTTTACGAATTGTAGTTTAAAGAAGTGTTTTCTGTTGGAGTTTCGGTAAAAGTTCTTTGGAATAGGACATCATAGAGGGTGAGAATCCCGTATTTGACCGTTACTTTCCAGCTGTGTGATACACTTTCAAAGAGTCGGATTGTTTGGGAATGCAGTCCAAAATGGGTGGTAAATTTCATCTAAAGCTAAATATTGGCGAGAGACCGATAGCGAACAAGTACCGTGAGGGAAAGATGAAAAGAACTTTGAAAAGAGAGTTAAACAGTACGTGAAATTGTCAAAAGGGAAGCGTTTGACACCAGTGTTGTTTAGTTGAAAATCAATTCAATAGTTTTTGATTCTAAGTAGTATTGACTTCATCAGCTTGCTGCTTTCTTCTCAGAAATTATTGAATGCACTTTTCAGTTAACAAGTCAACATCAATTTCTTTTGTTGTAAAAGGGTAAGAGGAAGGTGGCTTGCGCTTCGGCTCAAGTGTTTATAGCCTTTTATCTCTGCAATCGGAGAGATTGAGGTCTGCAGCGTAGATCCTTCGGGGTTAGATACATTGGTGCTAAGCTATAGCCTTGTATAGACAACTTGTTGACTATGCTCAGTCATGTAGCTTACTCGCTGATGCTATTTTACCTACGCTTAGGATGTTGA |
| Liebetanzomyces_isolate_Cel1A_Clone_4 | MT085728 | AACTTGATCTCAAATCAGATAAGAGTACCCGCTGAACTTAAGCATATCAATAAGCGGAGGAAAAGAAACTAACAAGGATTCCCCTAGTAACGGCGAGTGAAGCGGGAAGAGCTCAAATTTGAAATCTTAAAGGGTTTCCCTTTACGAATTGTAGTTTAAAGAAGTGTTTTCTGTTGGAGTTTCGGTAAAAGTTCTTTGGAATAGGACATCATAGAGGGTGAGAATCCCGTATTTGACCGTTACTTTCCAGCTGTGTGATACACTTTCAAAGAGTCGGATTGTTTGGGAATGCAGTCCAAAATGGGTGGTAAATTTCATCTAAAGCTAAATATTGGCGAGAGACCGATAGCGAACAAGTACCGTGAGGGAAAGATGAAAAGAACTTTGAAAAGAGAGTTAAACAGTACGTGAAATTGTCAAAAGGGAAGCGTTTGACACCAGTGTTGTTTAGTTGAAAATCAATTCAATAGTTTTTGATTCTAAGTAGTATTGACTTCATCAGCTTGCTGCTTTCTTCTCAGAAATTATTGAATGCACTTTTCAGTTAACAAGTCAACATCAATTTCTTTTGTTGTAAAAGGGTAAGAGGAAGGTGGCTTGCGCTTCGGCTCAAGTGTTTATAGCCTTTTATCTCTGCAATCGGAGAGATTGAGGTCTGCAGCGTAGATCCTTCGGGGTTAGATACATTGGTGCTAAGCTATAGCCTTGTATAGACAACTTGTTGACTATGCTCAGTCATGTAGCTTACTCGCTGATGCTATTTTACCTACGCTTAGGATGTTGA |
| Khyollomyces_ramosus_isolate_ZS33_Clone_8 | MT085710 | AACTTGATCTCAAATCAGATAAGAGTACCCGCTGAACTTAAGCATATCAATAAAGCGGAGGAAAAGAAACTAACAAGGATTCCCCTAGTAACGGCGAGTGAAGCGGGAAGAGCTCAAATTTGAAATCTTAAAGGGTTTCCCTTTACGAATTGTAGTTTAAAGAAGTGTTTTCTGTTGAGGTTTTGGTAAAAGTTCTTTGGAATAGGACATCATAGAGGGTGAGAATCCCGTATTTGACCTTTACTTTCAACTTTGTGATACACTTTCAAAGAGTCGGATTGTTTGGGAATGCAGTCCAAAATGGGTGGTAAATTTCATCTAAAGCTAAATATTGGCGAGAGACCGATAGCGAACAAGTACCGTGAGGGAAAGATGAAAAGAACTTTGAAAAGAGAGTTAAACAGTACGTGAAATTGTCAAAAGGGAAGCGTTTGACACCAGTGTTGTTTTTCTGAAAATCAATTAAAGAACTGTTTATGCTAAGTTGTTTAGACTTTATCAGCTAACTGTTTAGTTTTGAGCTTTTCTTTAATGCACTTTTCAGTTAACAAGTCAACATCAATTTCTTTTGCTGTAAAAAAGCTTGAGGAAGGTGGCTTGAGATTCGTCTTAAGTGTTTATAGCCTCTTGCCTATGCAGTCGGAGAGATTGAGGTCTGCAGCGTAGATCCTTCGGGGCTAAATGCATTTGTGCTAAGTTATAGCATTTTATAGACAACTTGTTGACTATGATTTGTCTTGTAGCTTACTCGCTTATGTTATTTTACTTACGCTTAGGATGTTGA |
| Khyollomyces_ZC_33 | MK881981.1 | ACGGCGAGTGAAGCGGGAAGAGCTCAAATTTGAAATCTTAAAGGGTTTCCCTTTACGAATTGTAGTTTAAAGAAGTGTTTTCTGTTGAGGTTTTGGTAAAAGTTCTTTGGAATAGGACATCATAGAGGGTGAGAATCCCGTATTTGACCTTTACTTTCAACTTTGTGATACACTTTCAAAGAGTCGGATTGTTTGGGAATGCAGTCCAAAATGGGTGGTAAATTTCATCTAAAGCTAAATATTGGCGAGAGACCGATAGCGAACAAGTACCGTGAGGGAAAGATGAAAAGAACTTTGAAAAGAGAGTTAAACAGTACGTGAAATTGTCAAAAGGGAAGCGTTTGACACCAGTGTTGTTTTTCTGAAAATCAATTAAAGAACTGTTTATGCTAAGTTGTTTAGACTTTATCAGCTAACTGTTTAGTTTTGAGCTTTTCTTTAATGCACTTTTCAGTTAACAAGTCAACATCAATTTCTTTTGCTGTAAAAAAGCTTGAGGAAGGTGGCTTGAGATTCGTCTTAAGTGTTTATAGCCTCTTGCCTATGCAGTCGGAGAGATTGAGGTCTGCAGCGTAGATCCTTCGGGGCTAAATGCATTTGTGCTAAGTTATAGCATTTTATAGACAACTTGTTGACTATGATTTGTCTTGTAGCTTACTCGCTTATGTTATTTTACCTACGCTTAGGATGTTGA |
| Khyollomyces_ZC_32 | MK881980.1 | ACGGCGAGTGAAGCGGGAAGAGCTCAAATTTGAAATCTTAAAGGGTTTCCCTTTACGAATTGTAGTTTAAAGAAGTGTTTTCTGTTGAGGTTTTGGTAAAAGTTCTTTGGAATAGGACATCATAGAGGGTGAGAATCCCGTATTTGACCTTTACTTTCAACTTTGTGATACACTTTCAAAGAGTCGGATTGTTTGGGAATGCAGTCCAAAATGGGTGGTAAATTTCATCTAAAGCTAAATATTGGCGAGAGACCGATAGCGAACAAGTACCGTGAGGGAAAGATGAAAAGAACTTTGAAAAGAGAGTTAAACAGTACGTGAAATTGTCAAAAGGGAAGCGTTTGACACCAGTGTTGTTTTTCTGAAAATCAATTAAAGAACTGTTTATGCTAAGTTGTTTAGACTTTATCAGCTAACTGTTTAGTTTTGAGCTTTTCTTTAATGCACTTTTCAGTTAACAAGTCAACATCAATTTCTTTTGCTGTAAAAAAGCTTGAGGAAGGTGGCTTGAGATTCGTCTTAAGTGTTTATAGCCTCTTGCCTATGCAGTCGGAGAGATTGAGGTCTGCAGCGTAGATCCTTCGGGGCTAAATGCATTTGTGCTAAGTTATAGCATTTTATAGACAACTTGTTGACTATGATTTGTCTTGTAGCTTACTCGCTTATGTTATTTTACCTACGCTTAGGATGTTGA |
| Khyollomyces_ZC_31 | MK881979.1 | ACGGCGAGTGAAGCGGGAAGAGCTCAAATTTCGAAATCTTAAAGGGTTTCCCTTTACGAATTGTAGTTTAAAGAAGTGTTTTCTGTTGAGGTTTTGGTAAAAGTTCTTTGGAATAGGACATCATAGAGGGTGAGAATCCCGTATTTGACCTTTACTTTCAACTTTGTGATACACTTTCAAAGAGTCGGATTGTTTGGGAATGCAGTCCAAAATGGGTGGTAAATTTCATCTAAAGCTAAATATTGGCGAGAGACCGATAGCGAACAAGTACCGTGAGGGAAAGATGAAAAGAACTTTGAAAAGAGAGTTAAACAGTACGTGAAATTGTCAAAAGGGAAGCGTTTGACACCAGTGTTGTTTTTCTGAAAATCAATTAAAGAACTGTTTATGCTAAGTTGTTTAGACTTTATCAGCTAACTGTTTAGTTTTGAGCTTTTCTTTAATGCACTTTTCAGTTAACAAGTCAACATCAATTTCTTTTGCTGTAAAAAAGCTTGAGGAAGGTGGCTTGAGATTCGTCTTAAGTGTTTATAGCCTCTTGCCTATGCAGTCGGAGAGATTGAGGTCTGCAGCGTAGATCCTTCGGGGCTAAATGCATTTGTGCTAAGTTATAGCATTTTATAGACAACTTGTTGACTATGATTTGTCTTGTAGCTTACTCGCTTATGTTATTTTACCTACGCTTAGGATGTTGA |
| Agriosomyces_longus_isolate_MS2_Clone_B | MT085709 | AACTTGATCTCAAATCAGATAAGAGTACCCGCTGAACTTAAGCATATCAATAGGCGGAGGAAAAGAAACTAACAAGGATTCCCCTAGTAACGGCGAGTGAAGCGGGAAGAGCTCAAATTTGAAATCTTAAAGGGTTTCCCTTTACGAATTGTAGTTTAAAGAAGTGTTTTCTGTTGAAATTTTGGTAAAAGTTCTTTGGAATAGGACATCATAGAGGGTGAGAATCCCGTATTTGACCATTATCTTCAGCTATGTGATACACTTTCAAAGAGTCGGATTGTTTGGGAATGCAGTCCAAAATGGGTGGTAAATTTCATCTAAAGCTAAATATTGGCGAGAGACCGATAGCGAACAAGTACCGTGAGGGAAAGATGAAAAGAACTTTGAAAAGAGAGTTAAACAGTACGTGAAATTGTCAAAAGGGAAGCGTTTGACACCAGTGTTGTTTCTCTGAAAATCAATTACAGGAAAGTTTATGTTAAGTTGTTAAGACTTTATCAGCTTACTGCTTAATTTTGAGCTTTTCTTTAATGCACTTTTCAGTTTACAAGTCAACATCAATTTCTTTTGTTGTAAAAAGGTAAGGGAAAGGTGGCTCAAGCTTCGGCTTAAGTGTTTATAGTCCTTTGCTTCTGCAATCGGAGAGATTGAGGTCTGCAGCGTAGATCCTTTTTTGGATTAGATACATTTGTGCTAAGTTACAGCTCTTTATAGACAACTTGTTGACTATGATCAGTCTTGTAGCTTACTCGCTTATGCTATTTTACCTACGCTTAGGATGTTGA |
| Agriosomyces_longus_isolate_MS2_Clone_C | MT085708 | AACTTGATCTCAAATCAGATAAGAGTACCCGCTGAACTTAAGCATATCAATAAGCGGAGGAAAAGAAACTAACAAGGATTCCCCTAGTAACGGCGAGTGAAGCGGGAAGAGCTCAAATTTGAAATCTTAAAGGGTTTCCCTTTACGAATTGTAGTTTAAAGAAGTGTTTTCTGTTGAAATTTTGGTAAAAGTTCTTTGGAATAGGACATCATAGAGGGTGAGAATCCCGTATTTGACCATTATCTTCAGCTATGTGATACACTTTCAAAGAGTCGGATTGTTTGGGAATGCAGTCCAAAATGGGTGGTAAATTTCATCTAAAGCTAAATATTGGCGAGAGACCGATAGCGAACGAGTACCGTGAGGGAAAGATGAAAAGAACTTTGAAAAGAGAGTTAAACAGTACGTGAAATTGTCAAAAGGGAAGCGTTTGACACCAGTGTTGTTTCTCTGAAAATCAATTACAGGAAAGTTTATGTTAAGTTGTTAAGACTTTATCAGCTTACTGCTTAATTTTGAGCTTTTCTTTAATGCACTTTTCAGTTTACAAGTCAACATCAATTTCTTTTGTTGTAAAAAGGTAAGGGAAAGGTGGCTCAAGCTTCGGCTTAAGTGTTTATAGTCCTTTGCTTCTGCAATCGGAGAGATTGAGGTCTGCAGCGTAGATCCTTTTTTGGATTAGATACATTTGTGCTAAGTTACAGCTCTTTATAGACAACTTGTTGACTATGATCAGTCTTGTAGCTTACTCGCTTATGCTATTTTACCTACGCTTAGGATGTTGA |
| Aestipascuomyces_R1_CloneA6 | MW019481.1 | AACTTGATCTCAAATCAGATAAGAGTACCCGCTGAACTTAAGCATATCAATAAGCGGAGGAAAAGAAACTAACAAGGATTCCCTTAGTAACGGCGAGTGAAGCGGGAAGAGCTCAAATTTGAAATCTTCAAGGTTCTACCTTGACGAATTGTAGTTTAAAGAAGTGTTTTCTGTTAGTGGAGTGGCAAAAGTTCTTTGGAATAGGACATCATAGAGGGTGAGAATCCCGTATTTGGTCATTCCTCCTAGCTTTGTGATACACTTTCAAAGAGTCGGATTGTTTGGGAATGCAGTCCAAAATGGGTGGTAAATTTCATCTAAAGCTAAATACTGGCGAGAGACCGATAGCGAACAAGTACCGTGAGGGAAAGATGAAAAGAACTTTGAAAAGAGAGTTAAACAGTACGTGAAATTGTCAAAAGGGAAGCGTTTGACACCAGTGTTGTTGTGGAGAAATCAATTGTGTGGAACGGGTTGTTGTGGGGTTGAGACCTTATCAGCTCTCCTTACTCTTTACCTTTTTCACGCAATGCACTTTCTCTTTAACAAGTCAACATCAATTTCTTTTGTTGTAAAAGGGTCAGCGGAAGGTGGCTTTCTTTTCGGAGAAGGTGTTTATAGCCGTTGATCTCTACAATCGGAGAGATTGAGGTCTGCAGCGTAGATCCTTCGGGGTTAGATACGTCGGTGCTAAGTTATAATCCTATATAGACAACTTGTTGACTATATTCGATCTTGTAGCTTACTCGCTGG |
| Aestipascuomyces_R1_CloneA1 | MW019480.1 | AACTTGATCTCAAATCAGATAAGAGTACCCGCTGAACTTAAGCATATCAATAAGCGGAGGAAAAGAAACTAACAAGGATTCCCTTAGTAACGGCGAGTGAAGCGGGAAGAGCTCAAATTTGAAATCTTCAAGGTTCTACCTTGACGAATTGTAGTTTAAAGAAGTGTTTTCTGTTAGTGGAGTGGCAAAAGTTCTTTGGAATAGGACATCATAGAGGGTGAGAATCCCGTATTTGGTCATTCCTCCTAGCTTTGTGATACACTTTCAAAGAGTCGGATTGTTTGGGAATGCAGTCCAAAATGGGTGGTAAATTTCATCTAAAGCTAAATACTGGCGAGAGACCGATAGCGAACAAGTACCGTGAGGGAAAGATGAAAAGAACTTTGAAAAGAGAGTTAAACAGTACGTGAAATTGTCAAAAGGGAAGCGTTTGACACCAGTGTTGTTGTGGAGAAATCAATTGTGTGGAACGGGTTGTTGTGGGGTTGAGACCTTATCAGCTCTCCTTACTCTTTACCTTTTTCACGCAATGCACTTTCTCTTTAACAAGTCAACATCAATTTCTTTTGTTGTAAAAGGGCCGGCGGAAGGTGGCTTTCTTTTCGGAGAAAGTGTTTATAGCCGTTGGTCTCTACAATCGGAGAGATTGAGGTCTGCAGCGTAGATCCTTCGGGGTTAGATACGTTGGTGCTAAGTTATAATCCTACATAGACAACTTGTTGACTATGTTCGATCATGTAGCTTACTCGCTGG |
| Buwchfawromyces_eastonii_isolate_GE09_28S_ribosomal_RNA_gene_partial_sequence | KP205570.1 | AACTTGATCTCAAATCAGATAAGAGTACCCGCTGAACTTAAGCATATCAATAAGCGGAGGAAAAGAAACTAACTAGGATTCCCTCAGTAACGGCGAGTGAAGCGGGAAGAGCTCAAATTTGAAATCTTCAAGGTTTTACCTTGACGAATTGTAGTTTAAAGAAGTGTTTTCTGTTGGAGTTTTGGTAAAAGTTCTTTGGAATAGGACATCATAGAGGGTGAGAATCCCGTATTTGACCATTATTTCCAGCTTTGTGATACACTTTCAAAGAGTCGGATTGTTTGGGAATGCAGTCCAAAATGGGTGGTAAATTTCATCTAAAGCTAAATATTGGCGAGAGACCGATAGCGAACAAGTACCGTGAGGGAAAGATGAAAAGAACTTTGAAAAGAGAGTTAAACAGTACGTGAAATTGTCAAAAGGGAAGCGTTTGACACCAGTGTTGTTTCTACGAAAATCAATTTATGATGGCTTGATGCTAAGCTTTAAGACTTTATCCGCTTGTTGTTTAGTTTTCTTGTCTTTGTGAATGCACTTTTCGTGGAACAAGTCAACATCAATTTCTTTTGCTGTAAAAGGGTAAGAGGAAGGTGGCTTCGGCTTCGGTTGAAGTGTTTATAGCCTTTTATCTCTGCAGTCGGAGAGATTGAGGTCTGCAGCGTAGACTCTTCGGGGTTAGATACGTCGGTGCTAAGCTATAGCTTTTCATAGACAACTTGTTGACTATGTTTAGTCATGTGGCTTACCCGCTGGCGCTATTTTACCTACGCTTAGGATG |
| Tahromyces_munnarensis_isolate_TDFKJa1926 | MT085676 | AACTTGATCTCAAATCAGATAAGAGTACCCGCTGAACTTAAGCATATCAATAAGCGGAGGAAAAGAAACTAACTAGGATTCCCTTAGTAACGGCGAGTGAAGCGGGAAGAGCTCAAATTTGAAATCTTCAAGGTTTTACCTTGACGAATTGTAGTTTAAAGAAGTGTTTTCTGTTGGAGTTTTGGTAAAAGTTCTTTGGAATAGGACATCATAGAGGGTGAGAATCCCGTATTTGACCATTTCTTCCAGCTTTGTGATACACTTTCAAAGAGTCGGATTGTTTGGGAATGCAGTCCAAAATGGGTGGTAAATTTCATCTAAAGCTAAATATTGGCGAGAGACCGATAGCGAACAAGTACCGTGAGGGAAAGATGAAAAGAACTTTGAAAAGAGAGTTAAACAGTACGTGAAATTGTCAAAAGGGAAGCGTTTGACACCAGTGTTGTTTTCTCGAAAATCAATTCATGATGGCTTGATGCTAAGGTAGTATTGACCTTAAAAGCTTGCTGCTTAGTTTTCTTGTCTTTGTGAATGCACTTTTCGTTTAACAAGTCAACATCAATTTCTTTTGCTGTAAAAGGGTAAGAGGAAGGTGGCTTCAGCTTCGGTTGAAGTGTTTATAGCCTTTTACCTCTGCAGTCGGAGAGATTGAGGTCTGCAGCGTAGACTCCTTCGGGGTTAGATACGTTTGTGCTAAGTTATAACTTTTTATAGACAACTTGTTGACTATGATTAGTCATGTAGCTTACTCGCAGACGCTATTTACCTAT |
| Tahromyces_munnarensis_isolate_TDFKJa193 | MT085675 | AACTTGATCTCAAATCAGATAAGAGTACCCGCTGAACTTAAGCATATCAATAAGCGGAGGAAAAGAAACTAACTAGGATTCCCTTAGTAACGGCGAGTGAAGCGGGAAGAGCTCAAATTTGAAATCTTCAAGGTTTTACCTTGACGAATTGTAGTTTAAAGAAGTGTTTTCTGTTGGAGTTTTGGTAAAAGTTCTTTGGAATAGGACATCATAGAGGGTGAGAATCCCGTATTTGACCATTTCTTCCAGCTTTGTGATACACTTTCAAAGAGTCGGATTGTTTGGGAATGCAGTCCAAAATGGGTGGTAAATTTCATCTAAAGCTAAATATTGGCGAGAGACCGATAGCGAACAAGTACCGTGAGGGAAAGATGAAAAGAACTTTGAAAAGAGAGTTAAACAGTACGTGAAATTGTCAAAAGGGAAGCGTTTGACACCAGTGTTGTTTTCTCGAAAATCAATTCATGATGGCTTGATGCTAAGGTAGTATTGACCTTAAAAGCTTGCTGCTTAGTTTTCTTGTCTTTGTGAATGCACTTTTCGTTTAACAAGTCAACATCAATTTCTTTTGCTGTAAAAGGGTAAGAGGAAGGTGGCTTCAGCTTCGGTTGAAGTGTTTATAGCCTTTTACCTCTGCAGTCGGAGAGATTGAGGTCTGCAGCGTAGACTCCTTCGGGGTTAGATACGTTTGTGCTAAGTTATAACTTTTTATAGACAACTTGTTGACTATGATTAGTCATGTAGCTTACTCGCAGACGCTATTACCTACGCTAGGA |
| Paucimyces_polynucleatus | MW694898.1 | AACTTGATCTCAAATCAGATAAGAGTACCCGCTGAACTTAAGCATATCAATAAGCGGAGGAAAAGAAACTAACAAGGATTCCCCTAGTAACGGCGAGTGAAGCGGGAAGAGCTCAAATTTGAAATCTTCAAGGGTTTCCCTTGACGAATTGTAGTTTATAGAAGTGTTTTCTGTTAGAGAGGTGGTAAAAGTTCTTTGGAATAGGACATCATAGAGGGTGAGAATCCCGTATTTGATCACTTTCTACTAACGTTGTGATACACTTTCAACGAGTCGGATTGTTTGGGAATGCAGTCCAAAATGGGTGGTAAATTTCATCTAAAGCTAAATATTGGCGAGAGACCGATAGCGAACAAGTACCGTGAGGGAAAGATGAAAAGAACTTTGAAAAGAGAGTTAAACAGTACGTGAAATTGTCAAAAGGGAAGCGTTTGACACCAGTGTTGTTTTTCCGAAAATCAATTACGAATGGCGGGATTCTAAGTTTCATGACCTTAAAAGCTTGTTTCTTTCTTTTCCTGTCTTTCTTAATGCACTTTTCGGTTAACAAGTCAACATCAATTTCTTTTGTTGTAAAAGGTTAAGAGGAAGGTGGCTTTACTTTCGGGTAAAGTGTTTATAGCCTTTTAACTCTGCAATCGGAGAGATTGAGGTCTGCAGCGTAGATCCTTCGGGGCTAGATACGTTTGTGCTAAGTTATAGCTTTTCATAGACAACTTGTTGACTATGTTTAGTCTTGTAGCTTACTCGCAGATGCTATTTTACCTACGCTTAGGATGT |
| Paucimyces_polynucleatus | MW694897.1 | AACTTGATCTCAAATCAGATAAGAGTACCCGCTGAACTTAAGCATATCAATAAGCGGAGGAAAAGAAACTAACAAGGATTCCCCTAGTAACGGCGAGTGAAGCGGGAAGAGCTCAAATTTGAAATCTTCAAGGGTTTCCCTTGACGAATTGTAGTTTATAGAAGTGTTTTCTGTTAGAGAGGTGGTAAAAGTTCTTTGGAATAGGACATCATAGAGGGTGAGAATCCCGTATTTGATCACTTTCTACTAACGTTGTGATACACTTTCAACGAGTCGGATTGTTTGGGAATGCAGTCCAAAATGGGTGGTAAATTTCATCTAAAGCTAAATATTGGCGAGAGACCGATAGCGAACAAGTACCGTGAGGGAAAGATGAAAAGAACTTTGAAAAGAGAGTTAAACAGTACGTGAAATTGTCAAAAGGGAAGCGTTTGACACCAGTGTTGTTTTTCCGAAAATCAATTACGAATGGCGGGATTCTAAGTTTCATGACCTTAAAAGCTTGTTTCTTTCTTTTCCTGTCTTTCTTAATGCACTTTTCGGTTAACAAGTCAACATCAATTTCTTTTGTTGTAAAAGGTTAAGAGGAAGGTGGCTTTACTTTCGGGTAAAGTGTTTATAGCCTTTTAACTCTGCAATCGGAGAGATTGAGGTCTGCAGCGTAGATCCTTCGGGGCTAGATACGTTTGTGCTAAGTTATAGCTTTTCATAGACAACTTGTTGACTATGTTTAGTCTTGTAGCTTACTCGCAGATGCTATTTTACCTACGCTTAGGATGT |
| Paucimyces_polynucleatus | MW694896.1 | AACTTGATCTCAAATCAGATAAGAGTACCCGCTGAACTTAAGCATATCAATAAGCGGAGGAAAAGAAACTAACAAGGATTCCCCTAGTAACGGCGAGTGAAGCGGGAAGAGCTCAAATTTGAAATCTTCAAGGGTTTCCCTTGACGAATTGTAGTTTATAGAAGTGTTTTCTGTTAGAGAGGTGGTAAAAGTTCTTTGGAATAGGACATCATAGAGGGTGAGAATCCCGTATTTGATCACTTTCTACTAACGTTGTGATACACTTTCAACGAGTCGGATTGTTTGGGAATGCAGTCCAAAATGGGTGGTAAATTTCATCTAAAGCTAAATATTGGCGAGAGACCGATAGCGAACAAGTACCGTGAGGGAAAGATGAAAAGAACTTTGAAAAGAGAGTTAAACAGTACGTGAAATTGTCAAAAGGGAAGCGTTTGACACCAGTGTTGTTTTTCCGAAAATCAATTACGAATGGCGGGATTCTAAGTTTCATGACCTTAAAAGCTTGTTTCTTTCTTTTCCTGTCTTTCTTAATGCACTTTTCGGTTAACAAGTCAACATCAATTTCTTTTGTTGTAAAAGGTTAAGAGGAAGGTGGCTTTACTTTCGGGTAAAGTGTTTATAGCCTTTTAACTCTGCAATCGGAGAGATTGAGGTCTGCAGCGTAGATCCTTCGGGGCTAGATACGTTTGTGCTAAGTTATAGCTTTTCATAGACAACTTGTTGACTATGTTTAGTCTTGTAGCTTACTCGCAGATGCTATTTACCTACGCTAGGATGT |
| Piromyces_sp._isolate_Jen1_Clone_1 | MT085712 | AACTTGATCTCAAATCAGATAAGAGTACCCGCTGAACTTAAGCATATCAATAAGCGGAGGAAAAGAAACTAACAAGGATTCCCTTAGTAACGGCGAGTGAAGCGGGAAGAGCTCAAATTTGAAATCTTCAAGGTTTTACCTTGACGAATTGTAGTTTAAAGAAGTGTTTTCTGTTGAAGTTTTGGTAAAAGTTCTTTGGAATAGGACATCATAGAGGGTGAGAATCCCGTATTTGACCATTATTTTCAGCTGTGTGATACACTTTCAAAGAGTCGGATTGTTTGGGAATGCAGTCCAAAATGGGTGGTAAATTTCATCTAAAGCTAAATATTGGCGAGAGACCGATAGCGAACAAGTACCGTGAGGGAAAGATGAAAAGAACTTTGAAAAGAGAGTTAAACAGTACGTGAAATTGTCAAAAGGGAAGCGTTTGACACCAGTGTTGTTTTCTCGAAAATCAATTAGAAAGAGTTGGATTTTGTGTTGTATTGACCTTAACAGCTTGCTTCACTCATTTCCTGCTTTTTTTAATGCACTTTTCGTTTAACAAGTCAACATCAGTTTCTTTTGTTGTAAAAGGGTCATTGGAAGGTGGCTTTCTCTTCGGAGAAAGTGTTTATAGCCTTTGATCTCTGCAATCGGAGAGACTGAGGTCTGCAGCGTAGATCCTTCGGGGTTAGATACATTTGTGCTAAGCTACAGCTTTTCATAGACAACTTGTTGACTATGTTTAGTCTTGTGACTTACCCGCTTATGCTATTTTACCTACGCTTAGGATGTTGA |
| Piromyces_sp._isolate_Jen1_Clone_2 | MT085713 | AACTTGATCTCAAATCAGATAAGAGTACCCGCTGAACTTAAGCATATCAATAAGCGGAGGAAAAGAAACTAACAAGGATTCCCTTAGTAACGGCGAGTGAAGCGGGAAGAGCTCAAATTTGAAATCTTCAAGGTTTTACCTTGACGAATTGTAGTTTAAAGAAGTGTTTTCTGTTGAAGTTTTGGTAAAAGTTCTTTGGAATAGGACATCATAGAGGGTGAGAATCCCGTATTTGACCATTATTTTCAGCTGTGTGATACACTTTCAAAGAGTCGGATTGTTTGGGAATGCAGTCCAAAATGGGTGGTAAATTTCATCTAAAGCTAAATATTGGCGAGAGACCGATAGCGAACAAGTACCGTGAGGGAAAGATGAAAAGAACTTTGAAAAGAGAGTTAAACAGTACGTGAAATTGTCAAAAGGGAAGCGTTTGACACCAGTGTTGTTTTCTCGAAAATCAATTAAAAAAAGTTGGATTTTGTGTTGTATTGACCTTAACAGCTTGCTTCACTCTTTTCCAGCTTTTTTTAATGCACTTTTCGTTTAACAAGTCAACATCAGTTTCTTTTGTTGTAAAAGGGTCATTGGAAGGTGGCTTTCTCTTCGGAGAAAGTGTTTATAGCCTTTGATCTCTGCAATCGGAGAGACTGAGGTCTGCAGCGTAGATCCTTCGGGGTTAGATACATTTGTGCTAAGCTACAGCTTTTCATAGACAACTTGTTGACTATGTTTAGTCTTGTGACTTACCCGCTTATGCTATTTTACCTACGCTTAGGATGTTGA |
| Piromyces_finnis_clone_1 | unpublished | ACGGCGAGTGAAGCGGGAAGAGCTCAAATTTGAAATCTTCAAGGTTTTACCTTGACGAATTGTAGTTTAAAGAAGTGTTTTCTGCTGAAGTTTTGGTAAAAGTTCTTTGGAATAGGACATCATAGAGGGTGAGAATCCCGTATTTGACCATTATTTTCAGCTTTGTGATACACTTTCAAAGAGTCGGATTGTTTGGGAATGCAGTCCAAAATGGGTGGTAAATTTCATCTAAAGCTAAATATTGGCGAGAGACCGATAGCGAACAAGTACCGTGAGGGAAAGATGAAAAGAACTTTGAAAAGAGAGTTAAACAGTACGTGAAATTGTCAAAAGGGAAGCGTTTGACACCAGTGTTGTTTTCTCGAAAATCAATTAAAAATGGCTCGATTTTGTATTGCTTGAGACCTTAAAAGCTCACTGTACATTTTTCGTGTCTTTTTTAATGCACTTTTCGTTTAACAAGTCAACATCAGTTTCTTTTGTTGTAAAAGGGTCATTGGAAGGTGGCTTTCTTTTCGGAGAAAGTGTTTATAGCCTTTGGCCTCTGCAATCGGAGAGACTGAGGTCTGCAGCGTAGACTCTTCGGGGTTAGATACATTTGTGCTAAGTTATAGCTTTTTATAGACAACTTGTTGACTATAATTAGTCTTGTAGCTTACTCGCTTATGCTATTTTACCTACGCTTAGGATGT |
| Piromyces_finnis_clone_2 | unpublished | ACGGCGAGTGAAGCGGGAAGAGCTCAAATTTGAAATCTTCAAGGTTTTACCTTGACGAATTGTAGTTTAAAGAAGTGTTTTCTGCTGAAGTTTTGGTAAAAGTTCTTTGGAATAGGACATCATAGAGGGTGAGAATCCCGTATTTGACCATTATTTTCAGCTTTGTGATACACTTTCAAAGAGTCGGATTGTTTGGGAATGCAGTCCAAAATGGGTGGTAAATTTCATCTAAAGCTAAATATTGGCGAGAGACCGATAGCGAACAAGTACCGTGAGGGAAAGATGAAAAGAACTTTGAAAAGAGAGTTAAACAGTACGTGAAATTGTCAAAAGGGAAGCGTTTGACACCAGTGTTGTTTTCTCGAAAATCAATTAAAAATGGCTCGATTTTGTATTGCTTGAGACCTTAAAAGCTCACTGTACATTTTTCGTGTCTTTTTTAATGCACTTTTCGTTTAACAAGTCAACATCAGTTTCTTTTGTTGTAAAAGGGTCATTGGAAGGTGGCTTTCTTTTCGGAGAAAGTGTTTATAGCCTTTGGCCTCTGCAATCGGAGAGACTGAGGTCTGCAGCGTAGACTCTTCGGGGTTAGATACATTTGTGCTAAGTTATAGCTTTTTATAGACAACTTGTTGACTATAATTAGTCTTGTAGCTTACTCGCTTATGCTATTTTACCTACGCTTAGGATG |
| Cyllamyces_Sp._isolate_TSB2_Clone_B12 | MT085703 | AACTTGATCTCAAATCAGATAAGAGTACCCGCTGAACTTAAGCATATCAATAAGCGGAGGAAAAGAAACTAACTAGGATTCCCTCAGTAACGGCGAGTGAAGCGGGAAGAGCTCAAATTTGAAATCTTCAAGGTTCTACCTTGACGAATTGTAGTTTAAAGAAGTGTTTTCTGTTGGAGTTTTGGTAAAAGTTCTTTGGAATAGGACATCATAGAGGGTGAGAATCCCGTATTTGACCATTATTTCCAACTTTGTGATACACTTTCAAAGAGTCGGATTGTTTGGGAATGCAGTCCAAAATGGGTGGTAAATTTCATCTAAAGCTAAATACTGGCGAGAGACCGATAGCGAACAAGTACCGTGAGGGAAAGATGAAAAGAACTTTGAAAAGAGAGTTAAACAGTACGTGAAATTGTCAAAAGGGAAGCGTTTGACACCAGTGTTGTTTTCTCGAAAATCAATTAAAAATGGCGGGATTTTGTGTTGTATTGACTTTATCAGCTTTCTGCACTTTTTTCCTGTCTTTCTTAATGCACTTTTCGTTTAACAAGTCAACATCAATTTCTTTTGTTGTAAAAGGGTCATTGGAAGGTGGCTTTCTTTTCGGAGAAAGTGTTTATAGCCTTTGGCCTCTGCAATCGGAGAGATTGAGGTCTGCAGCGTAGATCCTTCGGGGTTAGATACATTTGTGCTAAGCTATAGCCTTTCATAGACAACTTGTTGACTATGATCAGTCATGTAGCTTACTTGCTTATGCTATTTTACCTACGCTTAGGATGTTGA |
| Cyllamyces_Sp._isolate_TSB2_Clone_B9 | MT085706 | AACTTGATCTCAAATCAGATAAGAGTACCCGCTGAACTTAAGCATATCAATAAGCGGAGGAAAAGAAACTAACTAGGATTCCCTCAGTAACGGCGAGTGAAGCGGGAAGAGCTCAAATTTGAAATCTTCAAGGTTCTACCTTGACGAATTGTAGTTTAAAGAAGTGTTTTCTGTTGGAGTTTTGGTAAAAGTTCTTTGGAATAGGACATCATAGAGGGTGAGAATCCCGTATTTGACCATTATTTCCAACTTTGTGATACACTTTCAAAGAGTCGGATTGTTTGGGAATGCAGTCCAAAATGGGTGGTAAATTTCATCTAAAGCTAAATACTGGCGAGAGACCGATAGCGAACAAGTACCGTGAGGGAAAGATGAAAAGAACTTTGAAAAGAGAGTTAAACAGTACGTGAAATTGTCAAAAGGGAAGCGTTTGACACCAGTGTTGTTTTCTCGAAAATCAATTAAAAATGGCGGGATTTTGTGTTGTATTGACTTTATCAGCTTTCTGCACTTTTTTCCTGTCTTTTTTAATGCACTTTTCGTTTAACAAGTCAACATCAATTTCTTTTGTTGTAAAAGGGTCATTGGAAGGTGGCTTTCTTTTCGGAGAAAGTGTTTATAGCCTTTGGCCTCTGCAATCGGAGAGATTGAGGTCTGCAGCGTAGATCCTTCGGGGTTAGATACATTTGTGCTAAGCTATAGCCTTTCATAGACAACTTGTTGACTATGATCAGTCATGTAGCTTACTCGCTTATGCTATTTTACCTACGCTTAGGATGTTGA |
| Caecomyces_OF1_seq_2 | MZ044643 | AACTTGATCTCAAATCAGATAAGAGTACCCGCTGAACTTAAGCATATCAATAAGCGGAGGAAAAGGAACTAACTAGGATTCCCTCAGTAACGGCGAGTGAAGCGGGAAGAGCTCAAATTTGAAATCTTCAAGGTTTTACCTTGACGAATTGTAGTTTAAAGAAGTGTTTTCTGTTGGAATTTTGGTAAAAGTTCTTTGGAAAAGGACATCATAGAGGGTGAGAATCCCGTATTTGACCATTATTTCCAGCTTTGTGATACACTTTCAAAGAGTCGGATTGTTTGGGAATGCAGTCCAAAATGGGTGGTAAATTTCATCTAAAGCTAAATACTGGCGAGAGACCGATAGCGAACAAGTACCGTGAGGGAAAGATGAAAAGAACTTTGAAAAGAGAGTTAAACAGTACGTGAAATTGTCAAAAGGGAAGCGTTTGACACCAGTGTTGTTTTCTCGAAAATCAATTAAAAAAGACCGGATTTTGTGTTGTATTGACTTTATCAGCTTTCTGCACTCTTTTCTTGTCTTTTTTAATGCACTTTTCGTTTAACAAGTCAACATCAATTTCTTTTGTTGTAAAAGGGTCATTGGAAGGTGGCTTTCTTTTCGGAGAAAGTGTTTATAGCCTTTGGCCTCTGCAATCGGAGAGATTGAGGTCTGCAGCGTAGATCCTTCGGGGTTAGATACATTTGTGCTAAGTTATAGCCTTTCATAGACAACTTGTTGACTATGATCAGTCATGTAGCTTACTCGCTTATGCTATTTTACCTACGCTTAGGATGTTGA |
| Caecomyces_OF1_seq_3 | MZ044644 | AACTTGATCTCAAATCAGATAAGAGTACCCGCTGAACTTAAGCATATCAATAAGCGGAGGAAAAGAAACTAACTAGGATTCCCTCAGTAACGGCGAGTGAAGCGGGAAGAGCTCAAATTTGAAATCTTCAAGGTTTTACCTTGACGAATTGTAGTTTAAAGAAGTGTTTTCTGTTGGAGTTTTGGTAAAAGTTCTTTGGAAAAGGACATCATAGAGGGTGAGAATCCCGTATTTGACCATTATCTCCAGCTTTGTGATACACTTTCAAAGAGTCGGATTGTTTGGGAATGCAGTCCAAAATGGGTGGTAAATTTCATCTAAAGCTAAATACTGGCGAGAGACCGATAGCGAACAAGTACCGTGAGGGAAAGATGAAAAGAACTTTGAAAAGAGAGTTAAACAGTACGTGAAATTGTCAAAAGGGAAGCGTTTGACACCAGTGTTGTTTTCTCGAAAATCAATTAAAAAAGACCGGATTTTGTGTTGTATTGACTTTATCAGCTTTCTGCACTCTTTTCTTGTCTTTTTTAATGCACTTTTCGTTTAACAAGTCAACATCAATTTCTTTTGTTGTAAAAGGGTCATTGGAAGGTGGCTTTCTTTTCGGAGAAAGTGTTTATAGCCTTTGGCCTCTGCAATCGGAGAGATTGAGGTCTGCAGCGTAGATCCTTCGGGGTTAGATACATTTGTGCTAAGTTATAGCCTTTCATAGACAACTTGTTGACTATGATCAGTCATGTAGCTTACTCGCTTATGCTATTTTACCTACGCTTAGGATGTTGA |
| Caecomyces_OF1_seq_4 | MZ044645 | AACTTGATCTCAAATCAGATAAGAGTACCCGCTGAACTTAAGCATATCAATAAGCGGAGGAAAAGAAACTAACTAGGATTCCCTCAGTAACGGCGAGTGAAGCGGGAAGAGCTCAAATTTGAAATCTTCAAGGTTTTACCTTGACGAATTGTAGTTTAAAGAAGTGTTTTCTGTTGGAGTTTTGGTAAAAGTTCTTTGGAAAAGGACATCATAGAGGGTGAGAATCCCGTATTTGACCATTATCTCCAGCTTTGTGATACACTTTCAAAGAGTCGGATTGTTTGGGAATGCAGTCCAAAATGGGTGGTAAATTTCATCTAAAGCTAAATACTGGCGAGAGACCGATAGCGAACAAGTACCGTGAGGGAAAGATGAAAAGAACTTTGAAAAGAGAGTTAAACAGTACGTGAAATTGTCAAAAGGGAAGCGTTTGACACCAGTGTTGTTTTCTCGAAAATCAATTAAAAAAGACCGGATTTTGTGTTGTATTGACTTTATCAGCTTTCTGCACTCTTCTCTTGTCTTTTTTAATGCACTTTTCGTTTAACAAGTCAACATCAATTTCTTTTGTTGTAAAAGGGTCATTGGAAGGTGGCTTTCTTTTCGGAGAAAGTGTTTATAGCCTTTGGCCTCTGCAATCGGAGAGATTGAGGTCTGCAGCGTAGATCCTTCGGGGTTAGATACATTTGTGCTAAGTTATAGCCTTTCATAGGCAACTTGTTGACTATGATCAGTCATGTAGCTTACTCGCTTATGCTATTTTACCTACGCTTAGGATGTTGA |
| Feramyces_austinii_isolate_R4A_Clone_3 | MG584198.1 | AACTTGATCTCAAATCAGATAAGAGTACCCGCTGAACTTAAGCATATCAATAAGCGGAGGAAAAGAAACTAACAAGGATTCCCTTAGTAACGGCGAGTGAAGCGGGAAGAGCTCAAATTTGAAATCTTCAAGGTTCTACCTTGACGAATTGTAGTTTAAAGAAGTGTTTTCTGTTAGAGGAGTGGCAAAAGTTCTTTGGAGTAGGACATCATAGAGGGTGAGAATCCCGTATTTGGTCATTCTTTCTAGCTGTGTGATACACTTTCAAAGAGTCGGGCTGTTTGGGAATGCAGTCCAAAATGGGTGGTAAATTTCATCTAAAGCTAAATATTGGCGAGAGACCGATAGCGAACAAGTACCGTGAGGGAAAGATGAAAAGAACTTTGAAAAGAGAGTTAAACAGTACGTGAAATTGTCAAAAGGGAAGCGTTTGACACCAGTGTTGTTTTTCCGAAAATCAATTGTGCCTGGCTGGATTTTGTAGGAGTTTGACCTTACCAGCTTCTTCTGCTTTTTTCCGGTCTGGTGCAATGCACTTTTCGGTTAACAAGTCAACATCAATTTCTTTTGTTGTAAAAGGGTAATTGGAAGGTGGCTTTCTTTTCGGAGAAAGTGTTTATAGCCTTTTACCTCTACAATCGGAGAGATTGAGGTCTGCAGCGTAGATCCTTCGGGGTTAGATACATTTGTGCTAAGTTATAGCCCTGTATAGACAACTTGTTGACTATACTCGGTCATGTAGCTTACTCGCTTATGCTATTTTACCTACGCTTAGGATGTTGA |
| Feramyces_austinii_isolate_F3B_Clone_10 | MG584190.1 | AACTTGATCTCAAATCAGATAAGAGTACCCGCTGAACTTAAGCATATCAATAAGCGGAGGAAAAGAAACTAACAAGGATTCCCTTAGTAACGGCGAGTGAAGCGGGAAGAGCTCAAATTTGAAATCTTCAAGGTTCTACCTTGACGAATTGTAGTTTAAAGAAGTGTTTTCTGTTAGAGGAGTGGCAAAAGTTCTTTGGAATAGGACATCATAGAGGGTGAGAATCCCGTATTTGGTCATTCTTTCTAGCTGTGTGATACACTTTCAAAGAGTCGGATTGTTTGGGAATGCAGTCCAAAATGGGTGGTAAATTTCATCTAAAGCTAAATATTGGCGAGAGACCGATAGCGAACAAGTACCGTGAGGGAAAGATGAAAAGAACTTTGAAAAGAGAGTTAAACAGTACGTGAAATTGTCGAAAGGGAAGCGTTTGACACCAGTGTTGTTTTTCCGAAAATCAATTGTGCCTGGCTGGATTTTGTAGGAGTTTGACCTTATCAGCTTCTTCTGCTTTTTTCCGGTCTGGTGCAATGCACTTTTCGGTTAACAAGTCAACATCAATTTCTTTTGTTGTAAAAGGGTAATTGGAAGGTGGCTTTCTTTTCAGAGAAAGTGTTTATAGCCTTTTACCTCTACAATCGGAGAGATTGAGGTCTGCAGCGTAGATCCTTCAGGGTTAGATACATTTGTGCTAAGTTATAGCCCTGTATAGACAACTTGTTGACTATACTCGGTCATGTAGCTTACTCGCTTATGCTATTTACCTACGCTTAGGATGTTGA |
| Feramyces_austinii_isolate_F3A_Clone_3 | MG584193.1 | AACTTGATCTCAAATCAGATAAGAGTACCCGCTGAACTTAAGCATATCAATAAGCGGAGGAAAAGAAACTAACAAGGATTCCCTTAGTAACGGCGAGTGAAGCGGGAAGAGCTCAAATTTGAAATCTTCAAGGTTCTACCTTGACGAATTGTAGTTTAAAGAAGTGTTTTCTGTTAGAGGAGTGGCAAAAGTTCTTTGGAATAGGACATCATAGAGGGTGAGAATCCCGTATTTGGTCATTCTTTCTAGCTGTGTGATACACTTTCAAAGAGTCGGATTGTTTGGGAATGCAGTCCAAAATGGGTGGTAAATTTCATCTAAAGCTAAATATTGGCGAGAGACCGATAGCGAACAAGTACCGTGAGGGAAAGATGAAAAGAACTTTGAAAAGAGAGTTAAGCAGTACGTGAAATTGTCAAAAGGGAAGCGTTTGACACCAGTGTTGTTTTTCCGAAAATCAATTGTGCCTGGCTGGATTTTGTAGGAGTTTGACCTTATCAGCTTCTTCTGCTTTTTTCCGGTCTGGTGCAATGCACTTTTCGGTTAACAAGTCAACATCAATTTCTTTTGTTGTAAAAGGGTAATTGGAAGGTGGCTTTCTTTTCGGAGAAAGTGTTTATAGCCTTTTACCTCTACAATCGGAGAGATTGAGGTCTGCAGCGTAGATCCTTCGGGGTTAGATACATTTGTGCTAAGTTATAGCCCTGTATAGACAACTTGTTGACTATACTCGGTCATGTAGCTTACTCGCTTATGCTATTTTACCTACGCTTAGGATGTTGA |
| Neocallimastix_cameroonii_ABS_CaDo3a_TYPE | NG_60329.1 | GAAACTAACAAGGATTCCCTTAGTAACGGCGAGTGAAGCGGGAAGAGCTCAAATTTGAAATCTTCAAGGTTCTACCTTGACGAATTGTAGTTTAAAGAAGTGTTTTCTGTTAGAGGAGTAGCAAAAGTTCCTTGGAATGGGACATCATAGAGGGTGAGAATCCCGTATTTGGTTATTCTTTCTAGCTTTGTGATACACTTTCAAAGAGTCGGATTGTTTGGGAATGCAGTCCAAAATGGGTGGTAAATTTCATCTAAAGCTAAATATTGGCGAGAGACCGATAGCGAACAAGTACCGTGAGGGAAAGATGAAAAGAACTTTGAAAAGAGAGTTAAACAGTACGTGAAATTGTCAAAAGGGAAGCGTTTGACACCAGTGTTGTTTTTCCGAAAATCAATTAAGAAGGGCTTGATTTTGTGTTGTATTGACCTTATCAGCTTTCTTCACTCTTTTTCGAGTCTTTCTTAATGCACTTTTCGGTTAACAAGTCAACATCAATTTCTTTTGTTGTAAAAGGGTATTTGGAAGGTGGCTTTCTTTTCGGAGAAAGTGTTTATAGCCTTATACCTCTACAATCGGAGAGATTGAGGTCTGCAGCGTAGATCCTTCGGGGCTAGATACACTTGTGCTAAGTTATAGCTTTTCATAGACAACTTGTTGACTATGTTTAGTCATGTAGCTTACCCGCTTGTGCTATTTTACCTACGCTTAGGATGTTGA |
| Neocallimastix_cameroonii_28S | KR920745.1 | GAAACTAACAAGGATTCCCTTAGTAACGGCGAGTGAAGCGGGAAGAGCTCAAATTTGAAATCTTCAAGGTTCTACCTTGACGAATTGTAGTTTAAAGAAGTGTTTTCTGTTAGAGGAGTAGCAAAAGTTCCTTGGAATGGGACATCATAGAGGGTGAGAATCCCGTATTTGGTTATTCTTTCTAGCTTTGTGATACACTTTCAAAGAGTCGGATTGTTTGGGAATGCAGTCCAAAATGGGTGGTAAATTTCATCTAAAGCTAAATATTGGCGAGAGACCGATAGCGAACAAGTACCGTGAGGGAAAGATGAAAAGAACTTTGAAAAGAGAGTTAAACAGTACGTGAAATTGTCAAAAGGGAAGCGTTTGACACCAGTGTTGTTTTTCCGAAAATCAATTAAGAAGGGCTTGATTTTGTGTTGTATTGACCTTATCAGCTTTCTTCACTCTTTTTCGAGTCTTTCTTAATGCACTTTTCGGTTAACAAGTCAACATCAATTTCTTTTGTTGTAAAAGGGTATTTGGAAGGTGGCTTTCTTTTCGGAGAAAGTGTTTATAGCCTTATACCTCTACAATCGGAGAGATTGAGGTCTGCAGCGTAGATCCTTCGGGGCTAGATACACTTGTGCTAAGTTATAGCTTTTCATAGACAACTTGTTGACTATGTTTAGTCATGTAGCTTACCCGCTTGTGCTATTTTACCTACGCTTAGGATGTTGA |
| Neocallimastix_cameroonii_isolate_CaDo3a | ON695831.1 | GAAACTAACAAGGATTCCCTTAGTAACGGCGAGTGAAGCGGGAAGAGCTCAAATTTGAAATCTTCAAGGTTCTACCTTGACGAATTGTAGTTTAAAGAAGTGTTTTCTGTTAGAGGAGTAGCAAAAGTTCCTTGGAATGGGACATCATAGAGGGTGAGAATCCCGTATTTGGTTATTCTTTCTAGCTTTGTGATACACTTTCAAAGAGTCGGATTGTTTGGGAATGCAGTCCAAAATGGGTGGTAAATTTCATCTAAAGCTAAATATTGGCGAGAGACCGATAGCGAACAAGTACCGTGAGGGAAAGATGAAAAGAACTTTGAAAAGAGAGTTAAACAGTACGTGAAATTGTCAAAAGGGAAGCGTTTGACACCAGTGTTGTTTTTCCGAAAATCAATTAAGAAGGGCTTGATTTTGTGTTGTATTGACCTTATCAGCTTTCTTCACTCTTTTTCGAGTCTTTCTTAATGCACTTTTCGGTTAACAAGTCAACATCAATTTCTTTTGTTGTAAAAGGGTATTTGGAAGGTGGCTTTCTTTTCGGAGAAAGTGTTTATAGCCTTATACCTCTACAATCGGAGAGATTGAGGTCTGCAGCGTAGATCCTTCGGGGCTAGATACACTTGTGCTAAGTTATAGCTTTTCATAGACAACTTGTTGACTATGTTTAGTCATGTAGCTTACCCGCTTGTGCTATTTTACCTACGCTTAGGATGTTGA |
| Neocallimastix_cameroonii_clone_8 | MW175304.1 | AACTTGATCTCAAATCAGATAAGAGTACCCGCTGAACTTAAGCATATCAATAAGCGGAGGAAAAGAAACTAACAAGGATTCCCTTAGTAACGGCGAGTGAAGCGGGAAGAGCTCAAATTTGAAATCTTCAAGGTTCTACCTTGACGAATTGTAGTTTAAAAGAAGTGTTTTCTGTTAGAGGAGTAGCAAAAGTTCCTTGGAATGGGACATCATAGAGGGTGAGAATCCCGTATTTGGTTATTCTTTCTAGCTTTGTGATACACTTTCAAAGAGTCGGATTGTTTGGGAATGCAGTCCAAAATGGGTGGTAAATTTCATCTAAAGCTAAATATTGGCGAGAGACCGATAGCGAACAAGTACCGTGAGGGAAAGATGAAAAGAACTTTGAAAAGAGAGTTAAACAGTACGTGAAATTGTCAAAAGGGAAGCGTTTGACACCAGTGTTGTTTTTCCGAAAATCAATTAAGAGTGGCTTGATTTTGTGTTGTATTGACCTTATCAGCTTTCTTCACTCTTTTTCGAGTCTTTCTTAATGCACTTTTCGGTTAACAAGTCAACATCAATTTCTTTTGTTGTAAAAGGGTATTTGGAAGGTGGCTTTCTTTTCGGAGAAAGTGTTTATAGCCTTATACCTCTACAATCGGAGAGATTGAGGTCTGCAGCGTAGATCCTTCGGGGCTAGATACACTTGTGCTAAGTTATAGCTTTTCATAGACAACTTGTTGACTATGTTTAGTCATGTAGCTTACCCGCTTGTGCTATTTTACCTACGCTTAGGATGTTGA |
| Neocallimastix_cameroonii_isolate_G3_Clone7 | MT085722 | AACTTGATCTCAAATCAGATAAGAGTACCCGCTGAACTTAAGCATATCAATAAGCGGAGGAAAAGAAACTAACAAGGATTCCCTTAGTAACGGCGAGTGAAGCGGGAAGAGCTCAAATTTGAAATCTTCAAGGTTCTACCTTGACGAATTGTAGTTTAAAGAAGTGTTTTCTGTTAGAGGAGTAGCAAAAGTTCCTTGGAATGGGACATCATAGAGGGTGAGAATCCCGTATTTGGTTATTCTTTCTAGCTTTGTGATACACTTTCAAGGAGTCGGATTGTTTGGGAATGCAGTCCAAAATGGGTGGTAAATTTCATCTAAAGCTAAATATTGGCGAGAGACCGATAGCGAACAAGTACCGTGAGGGAAAGATGAAAAGAACTTTGAAAAGAGAGTTAAACAGTACGTGAAATTGTCAAAAGGGAAGCGTTTGACACCAGTGTTGTTTTTCCGAAAATCAATTACGAATGGCTTGATTTTGTGTTGTATTGACCTTATCAGCTTTCTTCACTCTTTTTCGAGTCTTTCTTAATGCACTTTTCGGTTAACAAGTCAACATCAATTTCTTTTGTTGTAAAAGGGTATTTGGAAGGTGGCTTTCTTTTCGGAGAAAGTGTTTATAGCCTTATACCTCTACAATCGGAGAGATTGAGGTCCGCAGCGTAGATCCTTCGGGGCTAGATACACTTGTGCTAAGTTATAGCTTTTCATAGACAACTTGTTGACTATGTTTAGTCATGTAGCTTACCCGCTTGTGCTATTTTACCTACGCTTAGGATGTTGA |
| Neocallimastix_cameroonii_clone_3 | MW175299.1 | AACTTGATCTCAAATCAGATAAGAGTACCCGCTGAACTTAAGCATATCAATAAGCGGAGGAAAAGAAACTAACAAGGATTCCCTTAGTAACGGCGAGTGAAGCGGGAAGAGCTCAAATTTGAAATCTTCAAGGTTCTACCTTGACGAATTGTAGTTTAAAGAAGTGTTTTCTGTTAGAGGAGTAGCAAAAGTTCCTTGGAATGGGACATCATAGAGGGTGAGAATCCCGTATTTGGTTATTCTTTCTAGCTTTGTGATACACTTTCAAAGAGTCGGATTGTTTGGGAATGCAGTCCAAAATGGGTGGTAAATTTCATCTAAAGCTAAATATTGGCGAGAGACCGATAGCGAACAAGTACCGTGAGGGAAAGATGAAAAGAACTTTGAAAAGAGAGTTAAACAGTACGTGAAATTGTCAAAAGGGAAGCGTTTGACACCAGTGTTGTTTTTCCGAAAATCAATTACGAATGGCTTGATTTTGTGTTGTATTGACCTTATCAGCTTTCTTCACTCTTTTTCGAGTCTTTCTTAATGCACTTTTCGGTTAACAAGTCAACATCAATTTCTTTTGTTGTAAAAGGGTATTTGGAAGGTGGCTTTCTTTTCGGAGAAAGTGTTTATAGCCTTATACCTCTACAATCGGAGAGATTGAGGTCTGCAGCGTAGATCCTTCGGGGCTAGATACACTTGTGCTAAGTTATAGCTTTTCATAGACAACTTGTTGACTATGTTTAGTCATGTAGCTTACCCGCTTGTGCTATTTTACCTACGCTTAGGATGTTGA |
| Neocallimastix_cameroonii_clone_4 | MW175300.1 | AACTTGATCTCAAATCAGATAAGAGTACCCGCTGAACTTAAGCATATCAATAAGCGGAGGAAAAGAAACTAACAAGGATTCCCTTAGTAACGGCGAGTGAAGCGGGAAGAGCTCAAATTTGAAATCTTCAAGGTTCTACCTTGACGAATTGTAGTTTAAAGAAGTGTTTTCTGTTAGAGGAGTAGCAAAAGTTCCTTGGAATGGGACATCATAGAGGGTGAGAATCCCGTATTTNGGTTATTCTTTCTAGCTTTGTGATACACTTTCAAAGAGTCGGATTGTTTGGGAATGCAGTCCAAAATGGGTGGTAAATTTCATCTAAAGCTAAATATTGGCGAGAGACCGATAGCGAACAAGTACCGTGAGGGAAAGATGAAAAGAACTTTGAAAAGAGAGTTAAACAGTACGTGAAATTGTCAAAAGGGAAGCGTTTGACACCAGTGTTGTTTTTCCGAAAATCAATTACGAATGGCTTGATTTTGTGTTGTATTGACCTTATCAGCTTTCTTCACTCTTTTTCGAGTCTTTCTTAATGCACTTTTCGGTTAACAAGTCAACATCAATTTCTTTTGTTGTAAAAGGGTATTTGGAAGGTGGCTTTCTTTTCGGAGAAAGTGTTTATAGCCTTATACCTCTACAATCGGAGAGATTGAGGTCTGCAGCGTAGATCCTTCGGGGCTAGATACACTTGTGCTAAGTTATAGCTTTTCATAGACAACTTGTTGACTATGTTTAGTCATGTAGCTTACCCGCTTGTGCTATTTTACCTACGCTTAGGATGTTGA |
| Neocallimastix_frontalis_NYF4 | JQ782545.1 | AACTTGATCTCAAATCAGATAAGAGTACCCGCTGAACTTAAGCATATCAATAAGCGGAGGAAAAGAAACTAACAAGGATTCCCTTAGTAACGGCGAGTGAAGCGGGAAGAGCTCAAATTTGAAATCTTCAAGGTTCTACCTTGACGAATTGTAGTTTAAAGAAGTGTTTTCTGTTAGATGAGTAGCAAAAGTTCCTTGGAATGGGACATCATAGAGGGTGAGAATCCCGTATTTGGTTATTTATTCTAGCTTTGTGATACACTTTCAAAGAGTCGGATTGTTTGGGAATGCAGTCCAAAATGGGTGGTAAATTTCATCTAAAGCTAAATATTGGCGAGAGACCGATAGCGAACAAGTACCGTGAGGGAAAGATGAAAAGAACTTTGAAAAGAGAGTTAAACAGTACGTGAAATTGTCAAAAGGGAAGCGTTTGACACCAGTGTTGTTTTTCCGAAAATCAATTACGAATGGCTTGATTTTGTGTTGTATTGACCTTATCAGCTTTCTTCACTTTTTTCGAGTCTTTCTTAATGCACTTTTCGGTTAACAAGTCAACATCAATTTCTTTTGTTGTAAAAGAGTATTCGGAAGGTGGCTTTCTTTTCGGAGAAAGTGTTTATAGCCCTATACTTCTACAATCGGAGAGATTGAGGTCTGCAGCGTAGATCCTTCGGGGTTAGATACATTTGTGCTAAGTTGTAGCTTTTCATAGACAACTTGTTGACTATGTTTAGTCATGCGGCTTACTCGCTTATGCTATTTTACCTACGCTTAGGATGTTGA |
| Neocallimastix_frontalis_NYF1 | JQ782542.1 | AACTTGATCTCAAATCAGATAAGAGTACCCGCTGAACTTAAGCATATCAATAAGCGGAGGAAAAGAAACTAACAAGGATTCCCTTAGTAACGGCGAGTGAAGCGGGAAGAGCTCAAATTTGAAATCTTCAAGGTTCTACCTTGACGAATTGTAGTTTAAAGAAGTGTTTTCTGTTAGATGAGTAGCAAAAGTTCCTTGGAATGGGACATCATAGAGGGTGAGAATCCCGTATTTGGTTATTCATTCTAGCTTTGTGATACACTTTCAAAGAGTCGGATTGTTTGGGAATGCAGTCCAAAATGGGTGGTAAATTTCATCTAAAGCTAAATATTGGCGAGAGACCGATAGCGAACAAGTACCGTGAGGGAAAGATGAAAAGAACTTTGAAAAGAGAGTTAAACAGTACGTGAAATTGTCAAAAGGGAAGCGTTTGACACCAGTGTTGTTTTTCCGAAAATCAATTACGAATGGCTTGATTTTGTGTTGTATTGACCTTATCAGCTTTCTTCACTTTTTTCGAGTCTTTCTTAATGCACTTTTCGGTTAACAAGTCAACATCAATTTCTTTTGTTGTAAAAGAGTATTCGGAAGGTGGCTTTCTTTTCGGAGAAAGTGTTTATAGCCCTATACTTCTACAATCGGAGAGATTGAGGTCTGCAGCGTAGATCCTTCGGGGTTAGATACATTTGTGCTAAGTTGTAGCTTTTCATAGACAACTTGTTGACTATGTTTAGTCATGCGGCTTACTCGCTTATGCTATTTTACCTACGCTTAGGTTGTTGA |
| Neocallimastix_frontalis_Hef5 | MT085723.1 | AACTTGATCTCAAATCAGATAAGAGTACCCGCTGAACTTAAGCATATCAATAAGCGGAGGAAAAGAAACTAACAAGGATTCCCTTAGTAACGGCGAGTGAAGCGGGAAGAGCTCAAATTTGAAATCTTCAAGGTTCTACCTTGACGAATTGTAGTTTAAAGAAGTGTTTTCTGTTAGATGAGTAGCAAAAGTTCCTTGGAATGGGACATCATAGAGGGTGAGAATCCCGTATTTGGTTATTCATTCTAGCTTTGTGATACACTTTCAAAGAGTCGGATTGTTTGGGAATGCAGTCCAAAATGGGTGGTAAATTTCATCTAAAGCTAAATATTGGCGAGAGACCGATAGCGAACAAGTACCGTGAGGGAAAGATGAAAAGAACTTTGAAAAGAGAGTTAAACAGTACGTGAAATTGTCAAAAGGGAAGCGTTTGACACCAGTGTTGTTTTTCCGAAAATCAATTACGAATGGCTTGATTTTGTGTTGTATTGACCTTATCAGCTTTCTTCACTTTTTTCGAGTCTTTCTTAATGCACTTTTCGGTTAACAAGTCAACATCAATTTCTTTTGTTGTAAAAGAGTATTCGGAAGGTGGCTTTCTTTTCGGAGAAAGTGTTTATAGCCCTATACTTCTACAATCGGAGAGATTGAGGTCTGCAGCGTAGATCCTTCGGGGTTAGATACATTTGTGCTAAGTTGTAGCTTTTCATAGACAACTTGTTGACTATGTTTAGTCGTGCGGCTTACTCGCTTATGCTATTTTACCTACGCTTAGGATGTTGA |
| Neocallimastix_frontalis_NYF2 | JQ782543.1 | AACTTGATCTCAAATCAGATAAGAGTACCCGCTGAACTTAAGCATATCAATAAGCGGAGGAAAAGAAACTAACAAGGATTCCCTTAGTAACGGCGAGTGAAGCGGGAAGAGCTCAAATTTGAAATCTTCAAGGTTCTACCTTGACGAATTGTAGTTTAAAGAAGTGTTTTCTGTTAGATGAGTAGCAAAAGTTCCTTGGAATGGGACATCATAGAGGGTGAGAATCCCGTATTTGGTTATTCATTCTAGCTTTGTGATACACTTTCAAAGAGTCGGATTGTTTGGGAATGCAGTCCAAAATGGGTGGTAAATTTCATCTAAAGCTAAATATTGGCGAGAGACCGATAGCGAACAAGTACCGTGAGGGAAAGATGAAAAGAACTTTGAAAAGAGAGTTAAACAGTACGTGAAATTGTCAAAAGGGAAGCGTTTGACACCAGTGTTGTTTTTCCGAAAATCAATTACGAATGGCTTGATTTTGTGTTGTATTGACCTTATCAGCTTTCTTCACTTTTTTCGAGTCTTTCTTAATGCACTTTTCGGTTAACAAGTCAACATCAATTTCTTTTGTTGTAAAAGAGTATTCGGAAGGTGGCTTTCTTTTCGGAGAAAGTGTTTATAGCCCTATACTTCTACAATCGGAGAGATTGAGGTCTGCAGCGTAGATCCTTCGGGGTTAGATACATTTGTGCTAAGTTGTAGCTTTTCATAGACAACTTGTTGACTATGTTTAGTCGTGCGGCTTACTCGCTTATGCTATTTTACCTACGCTTAGGATGTTGA |
| Neocallimastix_frontalis_NYR4 | JQ782549.1 | AACTTGATCTCAAATCAGATAAGAGTACCCGCTGAACTTAAGCATATCAATAAGCGGAGGAAAAGAAACTAACAAGGATTCCCTTAGTAACGGCGAGTGAAGCGGGAAGAGCTCAAATTTGAAATCTTCAAGGTTCTACCTTGACGAATTGTAGTTTAAAGAAGTGTTTTCTGTTAGATGAGTAGCAAAAGTTCCTTGGAATGGGACATCATAGAGGGTGAGAATCCCGTATTTGGGTTATTCATTCTAGCTTTGTGATACACTTTCAAAGAGTCGGATTGTTTGGGAATGCAGTCCAAAATGGGTGGTAAATTTCATCTAAAGCTAAATATTGGCGAGAGACCGATAGCGAACAAGTACCGTGAGGGAAAGATGAAAAGAACTTTGAAAAGAGAGTTAAACAGTACGTGAAATTGTCAAAAGGGAAGCGTTTGACACCAGTGTTGTTTTTCCGAAAATCAATTACGAATGGCTTGATTTTGTGTTGTATTGACCTTATCAGCTTTCTTCACTTTTTTCGAGTCTTTCTTAATGCACTTTTCGGTTAACAAGTCAACATCAATTTCTTTTGTTGTAAAAGAGTATTCGGAAGGTGGCTTTCTTTTCGGAGAAAGTGTTTATAGCCCTATACTTCTACAATCGGAGAGATTGAGGTCTGCAGCGTAGATCCTTCGGGGTTAGATACATTTGTGCTAAGTTGTAGCTTTTCATAGACAACTTGTTGACTATGTTTAGTCGTGCGGCTTACTCGCTTATGCTATTTTACCTACGCTTAGGATGTTGA |
| Neocallimastix_cameroonii_var_constans_1 | in this work | TAGTACGGCGAGTGAAGCGGGAAGAGCTCAAATTTGAAATCTTCAAGGTTCTACCTTGACGAATTGTAGTTTAAAGAAGTGTTTTCTGTTAGAGGAGTAGCAAAAGTTCCTTGGAATGGGACATCATAGAGGGTGAGAATCCCGTATTTGGTTATTCTTTCTAGCTTTGTGATACACTTTCAAAGAGTCGGATTGTTTGGGAATGCAGTCCAAAATGGGTGGTAAATTTCATCTAAAGCTAAATATTGGCGAGAGACCGATAGCGAACAAGTACCGTGAGGGAAAGATGAAAAGAACTTTGAAAAGAGAGTTAAACAGTACGTGAAATTGTCAAAAGGGAAGCGTTTGACACCAGTGTTGTTTTTCCGAAAATCAATTAAGAGTGGCTTGATTTTGTGTTGTATTGACCTTATCAGCTTTCTTCACTCTTTTTCGAGTCTTTCTTAATGCACTTTTCGGTTAACAAGTCAACATCAATTTCTTTTGTTGTAAAAGGGTATTTGGAAGGTGGCTTTCTTTTCGGAGAAAGTGTTTATAGCCTTATACCTCTACAATCGGAGAGATTGAGGTCTGCAGCGTAGATCCTTCGGGGCTAGATACACTTGTGCTAAGTTATAGCTTTTCATAGACAACTTGTTGACTATGTTTAGTCATGTAGCTTACCCGCTTGTGCTATTTTACCTACGCTTAGGATGTTGAAAGGGCGAATTCTGCAGATATCCATCACACTGGCGGCCGCTCGAGCATGCATCTAGAGGGCCCAATTCGCCCTATAGTGAGTCGTATTACAATTCACTGGCCGTCGTTTTACAACGTCGTGACTGGGAAAACCCTGGCGTTACCCACTA |
| Neocallimastix_cameroonii_var_constans_2 | in this work | AGTACGGCGAGTGAAGCGGGAAGAGCTCAAATTTGAAATCTTCAAGGTTCTACCTTGACGAATTGTAGTTTAAAGAAGTGTTTTCTGTTAGAGGAGTAGCAAAAGTTCCTTGGAATGGGACATCATAGAGGGTGAGAATCCCGTATTTGGTTATTCTTTCTAGCTTTGTGATACACTTTCAAAGAGTCGGATTGTTTGGGAATGCAGTCCAAAATGGGTGGTAAATTTCATCTAAAGCTAAATATTGGCGAGAGACCGATAGCGAACAAGTACCGTGAGGGAAAGATGAAAAGAACTTTGAAAAGAGAGTTAAACAGTACGTGAAATTGTCAAAAGGGAAGCGTTTGACACCAGTGTTGTTTTTCCGAAAATCAATTAAGAGTGGCTTGATTTTGTGTTGTATTGACCTTATCAGCTTTCTTCACTCTTTTTCGAGTCTTTCTTAATGCACTTTTCGGTTAACAAGTCAACATCAATTTCTTTTGTTGTAAAAGGGTATTTGGAAGGTGGCTTTCTTTTCGGAGAAAGTGTTTATAGCCTTATACCTCTACAATCGGAGAGATTGAGGTCTGCAGCGTAGATCCTTCGGGGCTAGATACACTTGTGCTAAGTTATAGCTTTTCATAGACAACTTGTTGACTATGTTTAGTCATGTAGCTTACCCGCTTGTGCTATTTTACCTACGCTTAGGATGTTGAAAGGGCGAATTCCAGCACACTGGCGGCCGTTACTAGTGGATCCGAGCTCGGTACCAAGCTTGGCGTAATCATGGTCATAGCTGTTTCCTGTGTGAAATTGTTATCCGCTCACAATTCCACACA |
| Neocallimastix_cameroonii_var_constans_3 | in this work | TAGTACGGCGAGTGAGCGGGAAGAGCTCAAATTTGAAATCTTCAAGGTTCTACCTTGACGAATTGTAGTTTAAAGAAGTGTTTTCTGTTAGAGGAGTAGCAAAAGTTCCTTGGAATGGGACATCATAGAGGGTGAGAATCCCGTATTTGGTTATTCTTTCTAGCTTTGTGATACACTTTCAAAGAGTCGGATTGTTTGGGAATGCAGTCCAAAATGGGTGGTAAATTTCATCTAAAGCTAAATATTGGCGAGAGACCGATAGCGAACAAGTACCGTGAGGGAAAGATGAAAAGAACTTTGAAAAGAGAGTTAAACAGTACGTGAAATTGTCAAAAGGGAAGCGTTTGACACCAGTGTTGTTTTTCCGAAAATCAATTAAGAGTGGCTTGATTTTGTGTTGTATTGACCTTATCAGCTTTCTTCACTCTTTTTCGAGTCTTTCTTAATGCACTTTTCGGTTAACAAGTCAACATCAATTTCTTTTGTTGTAAAAGGGTATTTGGAAGGTGGCTTTCTTTTCGGAGAAAGTGTTTATAGCCTTATACCTCTACAATCGGAGAGATTGAGGTCTGCAGCGTAGATCCTTCGGGGCTAGATACACTTGTGCTAAGTTATAGCTTTTCATAGACAACTTGTTGACTATGTTTAGTCATGTAGCTTACCCGCTTGTGCTATTTTACCTACGCTTAGGATGTTGAAAGGGCGAATTCTGCAGATATCCATCACACTGGCGGCCGCTCGAGCATGCATCTAGAGGGCCCAATTCGCCCTATAGTGAGTCGTATTACAATTCACTGGCCGTCGTTTTACAACGTCGTGACTGGGAAAACCCTGGCGTTACCAACTA |
| Neocallimastix_cameroonii_var_constans_4 | in this work | AGTACGGCGAGTGAGCGGGAAGAGCTCAAATTTGAAATCTTCAAGGTTCTACCTTGACGAATTGTAGTTTAAAGAAGTGTTTTCTGTTAGAGGAGTAGCAAAAGTTCCTTGGAATGGGACATCATAGAGGGTGAGAATCCCGTATTTGGTTATTCTTTCTAGCTTTGTGATACACTTTCAAAGAGTCGGATTGTTTGGGAATGCAGTCCAAAATGGGTGGTAAATTTCATCTAAAGCTAAATATTGGCGAGAGACCGATAGCGAACAAGTACCGTGAGGGAAAGATGAAAAGAACTTTGAAAAGAGAGTTAAACAGTACGTGAAATTGTCAAAAGGGAAGCGTTTGACACCAGTGTTGTTTTTCCGAAAATCAATTAAGAGTGGCTTGATTTTGTGTTGTATTGACCTTATCAGCTTTCTTCACTCTTTTTCGAGTCTTTCTTAATGCACTTTTCGGTTAACAAGTCAACATCAATTTCTTTTGTTGTAAAAGGGTATTTGGAAGGTGGCTTTCTTTTCGGAGAAAGTGTTTATAGCCTTATACCTCTACAATCGGAGAGATTGAGGTCTGCAGCGTAGATCCTTCGGGGCTAGATACACTTGTGCTAAGTTATAGCTTTTCATAGACAACTTGTTGACTATGTTTAGTCATGTAGCTTACCCGCTTGTGCTATTTTACCTACGCTTAGGATGTTGAAAGGGCGAATTCTGCAGATATCCATCACACTGGCGGCCGCTCGAGCATGCATCTAGAGGGCCCAATTCGCCCTATAGTGAGTCGTATTACAATTCACTGGCCGTCGTTTTACAACGTCGTGACTGGGAAAACCCTGGCGTTACCCACT |
| Neocallimastix_cameroonii_var_constans_5 | in this work | TAGTACGGCGAGTGAGCGGGAAGAGCTCAAATTTGAAATCTTCAAGGTTCTACCTTGACGAATTGTAGTTTAAAGAAGTGTTTTCTGTTAGAGGAGTAGCAAAAGTTCCTTGGAATGGGACATCATAGAGGGTGAGAATCCCGTATTTGGTTATTCTTTCTAGCTTTGTGATACACTTTCAAAGAGTCGGATTGTTTGGGAATGCAGTCCAAAATGGGTGGTAAATTTCATCTAAAGCTAAATATTGGCGAGAGACCGATAGCGAACAAGTACCGTGAGGGAAAGATGAAAAGAACTTTGAAAAGAGAGTTAAACAGTACGTGAAATTGTCAAAAGGGAAGCGTTTGACACCAGTGTTGTTTTTCCGAAAATCAATTAAGAGTGGCTTGATTTTGTGTTGTATTGACCTTATCAGCTTTCTTCACTCTTTTTCGAGTCTTTCTTAATGCACTTTTCGGTTAACAAGTCAACATCAATTTCTTTTGTTGTAAAAGGGTATTTGGAAGGTGGCTTTCTTTTCGGAGAAAGTGTTTATAGCCTTATACCTCTACAATCGGAGAGATTGAGGTCTGCAGCGTAGATCCTTCGGGGCTAGATACACTTGTGCTAAGTTATAGCTTTTCATAGACAACTTGTTGACTATGTTTAGTCATGTAGCTTACCCGCTTGTGCTATTTTACCTACGCTTAGGATGTTGAAAGGGCGAATTCTGCAGATATCCATCACACTGGCGGCCGCTCGAGCATGCATCTAGAGGGCCCAATTCGCCCTATAGTGAGTCGTATTACAATTCACTGGCCGTCGTTTTACAACGTCGTGACTGGGAAAACCCTGGCGTTACCCACTA |
| Neocallimastix_cameroonii_var_constans_6 | in this work | TAGTACGGCGAGTGAGCGGGAAGAGCTCAAATTTGAAATCTTCAAGGTTCTACCTTGACGAATTGTAGTTTAAAGAAGTGTTTTCTGTTAGAGGAGTAGCAAAAGTTCCTTGGAATGGGNCATCATAGAGGGTGAGAATCCCGTATTTGGTTATTCTTTCTAGCTTTGTGATACACTTTCAAAGAGTCGGATTGTTTGGGAATGCAGTCCAAAATGGGTGGTAAATTTCATCTAAAGCTAAATATTGGCGAGAGACCGATAGCGAACAAGTACCGTGAGGGAAAGATGAAAAGAACTTTGAAAAGAGAGTTAAACAGTACGTGAAATTGTCAAAAGGGAAGCGTTTGACACCAGTGTTGTTTTTCCGAAAATCAATTAAGAGTGGCTTGATTTTGTGTTGTATTGACCTTATCAGCTTTCTTCACTCTTTTTCGAGTCTTTCTTAATGCACTTTTCGGTTAACAAGTCAACATCAATTTCTTTTGTTGTAAAAGGGTATTTGGAAGGTGGCTTTCTTTTCGGAGAAAGTGTTTATAGCCTTATACCTCTACAATCGGAGAGATTGAGGTCTGCAGCGTAGATCCTTCGGGGCTAGATACACTTGTGCTAAGTTATAGCTTTTCATAGACAACTTGTTGACTATGTTTAGTCATGTAGCTTACCCGCTTGTGCTATTTTACCTACGCTTAGGATGTTGAAAGGGCGAATTCCAGCACACTGGCGGCCGTTACTAGTGGATCCGAGCTCGGTACCAAGCTTGGCGTAATCATGGTCATAGCTGTTTCCTGTGTGAAATTGTTATCCGCTCACAATTCACACA |
| Neocallimastix_cameroonii_var_constans_7 | in this work | AGTACGGCGAGTGANCGGGAAGAGCTCAAATTTGAAATCTTCAAGGTTCTACCTTGACGAATTGTAGTTTAAAGAAGTGTTTTCTGTTAGAGGAGTAGCAAAAGTTCCTTGGAATGGGACATCATAGAGGGTGAGAATCCCGTATTTGGTTATTCTTTCTAGCTTTGTGATACACTTTCAAAGAGTCGGATTGTTTGGGAATGCAGTCCAAAATGGGTGGTAAATTTCATCTAAAGCTAAATATTGGCGAGAGACCGATAGCGAACAAGTACCGTGAGGGAAAGATGAAAAGAACTTTGAAAAGAGAGTTAAACAGTACGTGAAATTGTCAAAAGGGAAGCGTTTGACACCAGTGTTGTTTTTCCGAAAATCAATTAAGAGTGGCTTGATTTTGTGTTGTATTGACCTTATCAGCTTTCTTCACTCTTTTTCGAGTTTTTCTTAATGCACTTTTCGGTTAACAAGTCAACATCAATTTCTTTTGTTGTAAAAGGGTATTTGGAAGGTGGCTTTCTTTTCGGAGAAAGTGTTTATAGCCTTATACCTCTACAATCGGAGAGATTGAGGTCTGCAGCGTAGATCCTTCGGGACTAGATACACTTGTGCTAAGTTATAGCTTTTCATAGACAACTTGTTGACTATGTTTAGTCATGTAGCTTACCCGCTTGTGCTATTTTACCTACGCTTAGGATGTTGAAGGGCGAATTCTGCAGATATCCATCACACTGGCGGCCGCTCGAGCATGCATCTAGAGGGCCCAATTCGCCCTATAGTGAGTCGTATTACAATTCACTGGCCGTCGTTTTACAACGTCGTGACTGGGAAACCCTGGCGTACCA |
| Neocallimastix_californiae_TN | MCOG00000000 | CGGCGAGTGAGCGGGAAGAGCTCAAATTTGAAATCTTCAAGGTTCTACCTTGACGAATTGTAGTTTAAAGAAGTGTTTTCTGTTAGAGGAGTAGCAAAAGTTCCTTGGAATGGGACATCATAGAGGGTGAGAATCCCGTATTTGGTTATTCTTTCTAGCTTTGTGATACACTTTCAAAGAGTCGGATTGTTTGGGAATGCAGTCCAAAATGGGTGGTAAATTTCATCTAAAGCTAAATATTGGCGAGAGACCGATAGCGAACAAGTACCGTGAGGGAAAGATGAAAAGAACTTTGAAAAGAGAGTTAAACAGTACGTGAAATTGTCAAAAGGGAAGCGTTTGACACCAGTGTTGTTTTTCCGAAAATCAATTAANAAGGACTTGATTTTGTGTTGTATTGACCTTATCAGCTTTCTTCACTCTTTTTCGAGTTTTTCTTAATGCACTTTTCGGTTAACAAGTCAACATCAATTTCTTTTGTTGTAAAAGGGTATTTGGAAGGTGGCTTTCTTTTCGGAGAAAGTGTTTATAGCCTTATACCTCTACAATCGGAGAGATTGAGGTCTGCAGCGTAGATCCTTCGGGGCTAGATACACTTGTGCTAAGTTATAGCTTTTCATAGACAACTTGTTGACTATGTTTAGTCATGTAGCTTACCCGCTTGTGCTATTTTACCTACGCTTAGGATGTTGAAAGGGCGAATTCCAGCACACTGGCGGCCGTTACTAGTGGATCCGAGCTCGGTACCAAGCTTGGCGTAATCATGGTCATAGCTGNTTCCTGTGTGAAANTGNTATCCGCTCACATTCCACACA |
| Neocallimastix_californiae_NCBI | OL957165.1 | GAAACTAACAAGGATTCCCTTAGTAACGGCGAGTGAAGCGGGAAGAGCTCAAATTTGAAATCTTCAAGGTTCTACCTTGACGAATTGTAGTTTAAAGAAGTGTTTTCTGTTAGAGGAGTAGCAAAAGTTCCTTGGAATGGGACATCATAGAGGGTGAGAATCCCGTATTTGGTTATTCTTTCTAGCTTTGTGATACACTTTCAAAGAGTCGGATTGTTTGGGAATGCAGTCCAAAATGGGTGGTAAATTTCATCTAAAGCTAAATATTGGCGAGAGACCGATAGCGAACAAGTACCGTGAGGGAAAGATGAAAAGAACTTTGAAAAGAGAGTTAAACAGTACGTGAAATTGTCAAAAGGGAAGCGTTTGACACCAGTGTTGTTTTTCCGAAAATCAATTAAGAAGGACTTGATTTTGTGTTGTATTGACCTTATCAGCTTTCTTCACTCTTTTTCGAGTTTTTCTTAATGCACTTTTCGGTTAACAAGTCAACATCAATTTCTTTTGTTGTAAAAGGGTATTTGGAAGGTGGCTTTCTTTTCGGAGAAAGTGTTTATAGCCTTATACCTCTACAATCGGAGAGATTGAGGTCTGCAGCGTAGATCCTTCGGGGCTAGATACACTTGTGCTAAGTTATAGCTTTTCATAGACAACTTGTTGACTATGTTTAGTCATGTAGCTTACCCGCTTGTGCTATTTTACCTACGCTTAGGATGTTGACAAAATGGTTTTAAACGACCCGTCTTGAAACACGGACCAAGGAGTCTAACAAATATGCGAGTATTTGAGTGGTAAACTCATATGCGTAATGAAAGTGATTTTGGTGGGATTTTCGATGCACCATCAACCGGTCAGGATTTTTTATGAGCGATCTGAGTCTAAGCATATTTGTTGGGACCCGAAAGATGGTGAACTATGCCTGAATAGGGTGAAGTCAGAGGAAACTCTGATGGAGGCTCGTAGCGGTTCTGACGTGCAAATC |
| Neocallimastix_lanati_TN | JACVTC000000000 | TCCCGTATTTGGTTATTCTTTCTAGCTTTGTGATACACTTTCAAAGAGTCGGATTGTTTGGGAATGCAGTCCAAAATGGGTGGTAAATTTCATCTAAAGCTAAATATTGGCGAGAGACCGATAGCGAACAAGTACCGTGAGGGAAAGATGAAAAGAACTTTGAAAAGAGAGTTAAACAGTACGTGAAATTGTCAAAAGGGAAGCGTTTGACACCAGTGTTGTTTTTCCGAAAATCAATTAAGAATGGCTTGATTTTGTGTTGTATTGACCTTATCAGCTTTCTTCACTCTTTTTCGAGTCTTTCTTAATGCACTTTTCGGTTAACAAGTCAACATCAATTTCTTTTGTTGTAAAAGGGTATTTGGAAGGTGGCTTTCTTTTCGGAGAAAGTGTTTATAGCCTTATACCTCTACAATCGGAGAGATTGAGGTCTGCAGCGTAGATCCTTCGGGGCTAGATACACTTGTGCTAAGTTATAGCTTTTCATAGACAACTTGTTGACTATGTTTAGTCATGTAGCTTACCCGCTTGTGCTATTTTACCTACGCTTAGGATGTTGACAAAATGGTTTTAAACGACCC |
| Pecoramyces_ruminatium_isolate_C1A_ctg7180000081852_whole_genome_shotgun_sequence | ASRE01020932.1 | AACTTGATCTCAAATCAGATAAGAGTACCCGCTGAACTTAAGCATATCAATAAGCGGAGGAAAAGAAACTAACAAGGATTCCCTTAGTAACGGCGAGTGAAGCGGGAAGAGCTCAAATTTGAAATCTTCAAGGTTCTACCTTGACGAATTGTAGTTTAAAGAAGTGTTTTCTGTTAGTGGAGTGGCAAAAGTTCTTTGGAATAGGACATCATAGAGGGTGAGAATCCCGTATTTGGTCATTCCTTCTAGCTTTGTGATACACTTTCAAAGAGTCGGATTGTTTGGGAATGCAGTCCAAAATGGGTGGTAAATTTCATCTAAAGCTAAATATTGGCGAGAGACCGATAGCGAACAAGTACCGTGAGGGAAAGATGAAAAGAACTTTGAAAAGAGAGTTAAACAGTACGTGAAATTGTCAAAAGGGAAGCGTTTGACACCAGTGTTGTTTTTCCGAAAATCAATTATAAATGTTTTGTTTCTGAGGAGTGATGACTTCATCGGCTTCCTTCTCTCTTTACGAAACTTTTGTAATGCACTTTTCGGTTAACAAGTCAACATCAATTTCTTTTGTTGTAAAAGAGTATTCGGAAGGTGGCTTTCTTTTCGGAGAAAGTGTTTATAGCCCTATACTTCTACAATCGGAGAGATTGAGGTCTGCAGCGTAGATCCTTCGGGGTTAGATACATTTGTGCTAAGTTATAGCTTTTCATAGACAACTTGTTGACTATGTTTAGTCTTGTAGCTTACTCGCTTATGCTATTTTACCTACGCTTAGGATGTTGA |
| Pecoramyces_ruminatium_isolate_C1A_ctg7180000078798_whole_genome_shotgun_sequence | ASRE01007038.1 | AACTTGATCTCAAATCAGATAAGAGTACCCGCTGAACTTAAGCATATCAATAAGCGGAGGAAAAGAAACTAACAAGGATTCCCTTAGTAACGGCGAGTGAAGCGGGAAGAGCTCAAATTTGAAATCTTCAAGGTTCTACCTTGACGAATTGTAGTTTAAAGAAGTGTTTTCTGTTAGTGGAGTGGCAAAAGTTCTTTGGAATAGGACATCATAGAGGGTGAGAATCCCGTATTTGGTCATTCCTTCTAGCTTTGTGATACACTTTCAAAGAGTCGGATTGTTTGGGAATGCAGTCCAAAATGGGTGGTAAATTTCATCTAAAGCTAAATATTGGCGAGAGACCGATAGCGAACAAGTACCGTGAGGGAAAGATGAAAAGAACTTTGAAAAGAGAGTTAAACAGTACGTGAAATTGTCAAAAGGGAAGCGTTTGACACCAGTGTTGTTTTTCCGAAAATCAATTATAAATGTTTTGTTTCTGAGGAGTGATGACTTCATCGGCTTCCTTCTCTCTTTACGAAACTTTTGTAATGCACTTTTCGGTTAACAAGTCAACATCAATTTCTTTTGTTGTAAAAGAGTATTCGGAAGGTGGCTTTCTTTTCGGAGAAAGTGTTTATAGCCCTATACTTCTACAATCGGAGAGATTGAGGTCTGCAGCGTAGATCCTTCGGGGTTAGATACATTTGTGCTAAGTTATAGCTTTTCATAGACAACTTGTTGACTATGTTTAGTCTTGTAGCTTACTCGCTTATGCTATTTTACCTACGCTTAGGATGTTGA |
| Pecoramyces_ruminatium_isolate_C1A_ctg7180000094010_whole_genome_shotgun_sequence | ASRE01022884.1 | AACTTGATCTCAAATCAGATAAGAGTACCCGCTGAACTTAAGCATATCAATAAGCGGAGGAAAAGAAACTAACAAGGATTCCCTTAGTAACGGCGAGTGAAGCGGGAAGAGCTCAAATTTGAAATCTTCAAGGTTCTACCTTGACGAATTGTAGTTTAAAGAAGTGTTTTCTGTTAGTGGAGTGGCAAAAGTTCTTTGGAATAGGACATCATAGAGGGTGAGAATCCCGTATTTGGTCATTCCTTCTAGCTTTGTGATACACTTTCAAAGAGTCGGATTGTTTGGGAATGCAGTCCAAAATGGGTGGTAAATTTCATCTAAAGCTAAATATTGGCGAGAGACCGATAGCGAACAAGTACCGTGAGGGAAAGATGAAAAGAACTTTGAAAAGAGAGTTAAACAGTACGTGAAATTGTCAAAAGGGAAGCGTTTGACACCAGTGTTGTTTTTCCGAAAATCAATTATAAATGTTTTGTTTCTGAGGAGTGATGACTTCATCGGCTTCCTTCTCTCTTTACGAAACTTTTGTAATGCACTTTTCGGTTAACAAGTCAACATCAATTTCTTTTGTTGTAAAAGAGTATTCGGAAGGTGGCTTTCTTTTCGGAGAAAGTGTTTATAGCCCTATACTTCTACAATCGGAGAGATTGAGGTCTGCAGCGTAGATCCTTCGGGGTTAGATACATTTGTGCTAAGTTATAGCTTTTCATAGACAACTTGTTGACTATGTTTAGTCTTGTAGCTTACTCGCTTATGCTATTTTACCTACGCTTAGGATGTTGA |
| Ghazallomyces_constrictus_isolate_AXS31_Clone_B2 | MT085695 | AACTTGATCTCAAATCAGATAAGAGTACCCGCTGAACTTAAGCATATCAATAAGCGGAGGAAAAGAAACTAACAAGGATTCCCTTAGTAACGGCGAGTGAAGCGGGAAGAGCTCAAATTTGAAATCTTCAAGGTTCTACCTTGACGAATTGTAGTTTAGAGAAGTGTTTTCTGCTAGTGGAGTGGCAAAAGTTCTTTGGAATAGGACATCATAGAGGGTGAGAATCCCGTATTTGGTCATTCTTTCTAGCTTTGTGATACACTTTCAAAGAGTCGGATTGTTTGGGAATGCAGTCCAAAATGGGTGGTAAATTTCATCTAAAGCTAAATATTGGCGAGAGACCGATAGCGAACAAGTACCGTGAGGGAAAGATGAAAAGAACTTTGAAAAGAGAGTTAAACAGTACGTGAAATTGTCAAAAGGGAAGCGTTTGACACCAGTGTTGTTTTTCCGAAAATCAATTATGAATGTTTTGTTTTTGAGGAGTTATGACTTCATCGGCTTGCTCCTTTCTTTACGGAACTTTCGTAATGCACTTTTCGGTTAACAAGTCAACATCAATTTCTTTTGTTGTAAAAGGGTACGAGGAAGGTGGCTTTCTTTTCGGAGAAAGTGTTTATAGCCTTGTGCCTCTGCAATCGGAGAGATTGAGGTCTGCAGCGTAGATCCTTCGGGGCTAGATACACTTGTGCTAAGTTATAGCTTTTCATAGACAACTTGTTGACTATGTTTAGTCTTGTAGCTTACCCGCTTGTGCTATTTTACCTACGCTTAGGATGTTGA |
| Ghazallomyces_constrictus_isolate_AXS31_Clone_B3 | MT085694 | AACTTGATCTCAAATCAGATAAGAGTACCCGCTGAACTTAAGCATATCAATAAGCGGAGGAAAAGAAACTAACAAGGATTCCCTTAGTAACGGCGAGTGAAGCGGGAAGAGCTCAAATTTGAAATCTTCAAGGTTCTACCTTGACGAATTGTAGTTTAGAGAAGTGTTTTCTGCTAGTGGAGTGGCAAAAGTTCTTTGGAATAGGACATCATAGAGGGTGAGAATCCCGTATTTGGTCATTCTTTCTAGCTTTGTGATACACTTTCAAAGAGTCGGATTGTTTGGGAATGCAGTCCAAAATGGGTGGTAAATTTCATCTAAAGCTAAATATTGGCGAGAGACCGATAGCGAACAAGTACCGTGAGGGAAAGATGAAAAGAACTTTGAAAAGAGAGTTAAACAGTACGTGAAATTGTCAAAAGGGAAGCGTTTGACACCAGTGTTGTTTTTCCGAAAATCAATTATGAATGTTTTGTTTTTGAGGAGTTATGACTTCATCGGCTTGCTCCTTTCTTTACGGAACTTTCGTAATGCACTTTTCGGTTAACAAGTCAACATCAATTTCTTTTGTTGTAAAAGGGTACGAGGAAGGTGGCTTTCTTTTCGGAGAAAGTGTTTATAGCCTTGTGCCTCTGCAATCGGAGAGATTGAGGTCTGCAGCGTAGATCCTTCGGGGCTAGATACACTTGTGCTAAGTTATAGCTTTTCATAGACAACTTGTTGACTATGTTTAGTCTTGTAGCTTACCCGCTTGTGCTATTTTACCTACGCTTAGGATGTTGA |
| Ghazallomyces_constrictus_isolate_AXS31_Clone_B1 | MT085693 | AACTTGATCTCAAATCAGATAAGAGTACCCGCTGAACTTAAGCATATCAATAAGCGGAGGAAAAGAAACTAACAAGGATTCCCTTAGTAACGGCGAGTGAAGCGGGAAGAGCTCAAATTTGAAATCTTCAAGGTTCTACCTTGACGAATTGTAGTTTAAAGAAGTGTTTTCTGCTAGTGGAGTGGCAAAAGTTCTTTGGAATAGGACATCATAGAGGGTGAGAATCCCGTATTTGGTCATTCTTTCTAGCTTTGTGATACACTTTCAAAGAGTCGGATTGTTTGGGAATGCAGTCCAAAATGGGTGGTAAATTTCATCTAAAGCTAAATATTGGCGAGAGACCGATAGCGAACAAGTACCGTGAGGGAAAGATGAAAAGAACTTTGAAAAGAGAGTTAAACAGTACGTGAAATTGTCAAAAGGGAAGCGTTTGACACCAGTGTTGTTTTTCCGAAAATCAGTTATGAATGTTTTTGTTTTTGAGGAGTTATGACTTCATCGGCTTGCTCCTTTCTTTACGGAACTTTCGTAATGCACTTTTCGGTTAACAAGTCAACATCAATTTCTTTTGTTGTAAAAGGGTATGAGGAAGGTGGCTTTCTTTTCGGAGAAAGTGTTTATAGCCTTGTACCTCTGCAATCGGAGAGATTGAGGTCTGCAGCGTAGATCCTTCGGGGCTAGATACACTTGTGCTAAGTTATAGCTTTTCATAGACAACTTGTTGACTATGTTTAGTCATGTAGCTTACCCGCTTGTGCTATTTTACCTACGCTTAGGATGTTAA |
| Orpinomyces_joyonii_isolate_D3A_Clone_H09 | MT085734 | AACTTGATCTCAAATCAGATAAGAGTACCCGCTGAACTTAAGCATATCAATAAGCGGAGGAAAAGAAACTAACAAGGATTCCCTTAGTAACGGCGAGTGAAGCGGGAAGAGCTCAAATTTGAAATCTTCAAGGTTCTACCTTGACGAATTGTAGTTTATAGAAGTGTTTTCTGTTAGTGGAGTGGCAAAAGTTCTTTGGAATAGGACATCATAGAGGGTGAGAATCCCGTATTTGGTCATTCCTCCTAGCGTTGTGATACACTTTCAACGAGTCGGATTGTTTGGGAATGCAGTCCAAAATGGGTGGTAAATTTCATCTAAAGCTAAATATTGGCGAGAGACCGATAGCGAACAAGTACCGTGAGGGAAAGATGAAAAGAACTTTGAAAAGAGAGTTAAACAGTACGTGAAATTGTCAAAAGGGAAGCGTTTGACACCAGTGTTGTTTTTCCGAAAATCAATTACGAATGTTTTGTTTTTAAGAAGTTATGACTTCATCGGCTTGCTTCTTTCTTTATGAAACTTTCTTAATGCACTTTTCGGTTAACAAGTCAACATCAATTTCTTTTGTTGTAAAGGGGTATGAGGAAGGTGGCTTTCTTTTCGGAGAAAGTGTTTATAGCCTTGTGCCTCTGCAATCGGAGAGATTGAGGTCTGCAGCGTAGATCCTTCGGGGTTAGATACATTTGTGCTAAGTTATAGCTTTTCATAGACAACTTGTTGACTATGTTTAGTCTTGTAGCTTACTCGCTTATGTTATTTTACCTACGCTTAGGATGTTGA |
| Orpinomyces_joyonii_isolate_D3A_Clone_F11 | MT085736 | AACTTGATCTCAAATCAGATAAGAGTACCCGCTGAACTTAAGCATATCAATAAGCGGAGGAAAAGAAACTAACAAGGATTCCCTTAGTAACGGCGAGTGAAGCGGGAAGAGCTCAAATTTGAAATCTTCAAGGTTCTACCTTGACGAATTGTAGTTTATAGAAGTGTTTTCTGTTAGTGGAGTGGCAAAAGTTCTTTGGAATAGGACATCATAGAGGGTGAGAATCCCGTATTTGGTCATTCCTCCTAGCGTTGTGATACACTTTCAACGAGTCGGATTGTTTGGGAATGCAGTCCAAAATGGGTGGTAAATTTCATCTAAAGCTAAATATTGGCGAGAGACCGATAGCGAACAAGTACCGTGAGGGAAAGATGAAAAGAACTTTGAAAAGAGAGTTAAACAGTACGTGAAATTGTCAAAAGGGAAGCGTTTGACACCAGTGTTGTTTTTCCGAAAATCAATTACGAATGTTTTATTTTTGAGGAGTTATGACTTCATCGGCTTGCTCCTTTCTTTATGAAACTTTCTTAATGCACTTTTCGGTTAACAAGTCAACATCAATTTCTTTTGTTGTAAAAGGGTATGAGGAAGGTGGCTTTCTTTTCGGAGAAAGTGTTTATAGCCTTGTGCCTCTGCAATCGGAGAGATTGAGGTCTGCAGCGTAGATCCTTCGGGGTTAGATACGTTTGTGCTAAGTTATAGCTTTTCATAGACAACTTGTTGACTATGTTTAGTCTTGTAGCTTACTCCCTTACGCTATTTTACCTACGCTTAGGATGTTGA |
| Orpinomyces_joyonii_isolate_D3A_Clone_G09 | MT085733 | AACTTGATCTCAAATCAGATAAGAGTACCCGCTGAACTTAAGCATATCAATAAGCGGAGGAAAAGAAACTAACAAGGATTCCCTTAGTAACGGCGAGTGAAGCGGGAAGAGCTCAAATTTGAAATCTTCAAGGTTCTACCTTGACGAATTGTAGTTTATAGAAGTGTTTTCTGTTAGTGGAGTGGCAAAAGTTCTTTGGAATAGGACATCATAGAGGGTGAGAATCCCGTATTTGGTCATTCCTCCTAGCGTTGTGATACACTTTCAACGAGTCGGATTGTTTGGGAATGCAGTCCAAAATGGGTGGTAAATTTCATCTAAAGCTAAATATTGGCGAGAGACCGATAGCGAACAAGTACCGTGAGGGAAAGATGAAAAGAACTTTGAAAAGAGAGTTAAACAGTACGTGAAATTGTCAAAAGGGAAGCGTTTGACACCAGTGTTGTTTTTCCGAAAATCAATTACGAATGTTTTATTTTTGAGGAGTTATGACTTCATCGGCTTGCTCCTTTCTTTATGAAACTTTCTTAATGCACTTTTCGGTTAACAAGTCAACATCAATTTCTTTTGTTGTAAAAGGGTATGAGGAAGGTGGCTTTCTTTTCGGAGAAAGTGTTTATAGCCTTGTGCCTCTGCAATCGGAGAGATTGAGGTCTGCAGCGTAGATCCTTCGGGGTTAGATACGTTTGTGCTAAGTTATAGCTTTTCATAGACAACTTGTTGACTATGTTTAGTCTTGTAGCTTACTCGCTTACGCTATTTTACCTACGCTTAGGATGTTGA |

**Tables S2 through S13**. Top differentially upregulated and downregulated CAZyme genes for the different pairwise conditions.

**Reed canary grass vs. Glucose**

most upregulated

|  | ProteinID | Annotation | Substrate | Log2 Fold Change | p adjusted |
| --- | --- | --- | --- | --- | --- |
| 1 | 821051 | Carbohydrate-Binding Module Family 6, Glycoside Hydrolase Family 43 | xylan, beta-glucan, cellulose | 11.17 | 2.41E-25 |
| 2 | 834799 | Glycoside Hydrolase Family 11 | xylan | 11.14 | 5.34E-09 |
| 3 | 821868 | Glycoside Hydrolase Family 11 | xylan | 11.06 | 9.86E-22 |
| 4 | 640910 | Glycoside Hydrolase Family 11 x2 | xylan | 10.91 | 9.86E-36 |
| 5 | 834800 | Glycoside Hydrolase Family 11 | xylan | 10.67 | 6.25E-11 |
| 6 | 912130 | Carbohydrate Esterase Family 4 | chitin | 10.67 | 3.26E-26 |
| 7 | 807834 | Carbohydrate-Binding Module Family 13, Dockerin | xylan | 10.62 | 1.17E-20 |
| 8 | 885928 | Carbohydrate-Binding Module Family 6, Glycoside Hydrolase Family 43 | xylan | 10.59 | 1.51E-27 |
| 9 | 909407 | Glycoside Hydrolase Family 6 | cellulose | 10.57 | 1.87E-17 |
| 10 | 909541 | Carbohydrate-Binding Module Family 1, Carbohydrate Esterase Family 3 | xylan | 10.44 | 2.59E-25 |

most downregulated

|  | ProteinID | Annotation | Substrate | Log2 Fold Change | p adjusted |
| --- | --- | --- | --- | --- | --- |
| 1 | 900407 | Dockerin, Glycoside Hydrolase Family 5 / Subf 4 | beta-glucan, xylan, beta-mannan | -11.06 | 1.68E-16 |
| 2 | 830849 | Glycoside Hydrolase Family 5 / Subf 4 | beta-glucan, xylan, beta-mannan | -9.79 | 2.40E-13 |
| 3 | 781227 | Dockerin x2, Glycoside Hydrolase Family 5 / Subf 4 | beta-glucan, xylan, beta-mannan | -8.99 | 7.85E-33 |
| 4 | 900393 | Dockerin, Glycoside Hydrolase Family 5 / Subf 4 | beta-glucan, xylan, beta-mannan | -8.24 | 4.68E-70 |
| 5 | 900404 | Dockerin, Glycoside Hydrolase Family 5 / Subf 4 | beta-glucan, xylan, beta-mannan | -8.21 | 2.59E-27 |
| 6 | 599515 | Carbohydrate-Binding Module Family 10 x3, Dockerin x2, Glycoside Hydrolase Family 5 / Subf 5 x2 | beta-glucan | -7.90 | 1.00E-89 |
| 7 | 777321 | Glycoside Hydrolase Family 5 / Subf 7 | - | -7.64 | 8.48E-59 |
| 8 | 777322 | Glycoside Hydrolase Family 5 / Subf 7 | - | -7.33 | 4.66E-40 |
| 9 | 606303 | Dockerin | - | -6.79 | 1.34E-23 |
| 10 | 626032 | Dockerin x2, Glycoside Hydrolase Family 48 | cellulose, cellulose, chitin | -5.89 | 1.22E-61 |

**Reed canary grass vs. Cellobiose**

most upregulated

|  | ProteinID | Annotation | Substrate | Log2 Fold Change | p adjusted |
| --- | --- | --- | --- | --- | --- |
| 1 | 885928 | Carbohydrate-Binding Module Family 6, Glycoside Hydrolase Family 43 | xylan, beta-glucan, cellulose | 15.23 | 9.01E-28 |
| 2 | 813000 | Carbohydrate-Binding Module Family 1, Glycoside Hydrolase Family 11 | xylan | 15.12 | 1.68E-18 |
| 3 | 781642 | Carbohydrate Esterase Family 1, Dockerin x2, Glycoside Hydrolase Family 11 | xylan | 12.95 | 1.62E-103 |
| 4 | 909005 | Carbohydrate Esterase Family 3, Dockerin | xylan | 12.65 | 3.91E-32 |
| 5 | 640910 | Glycoside Hydrolase Family 11 x2 | xylan | 12.59 | 2.57E-32 |
| 6 | 896678 | Glycoside Hydrolase Family 3 | beta-glucan | 12.49 | 4.20E-29 |
| 7 | 788482 | Carbohydrate-Binding Module Family 1, Glycoside Hydrolase Family 11 | xylan | 11.89 | 5.16E-24 |
| 8 | 817649 | Glycoside Hydrolase Family 3 | beta-glucan, xylan, beta-glucan, beta-glucan | 11.84 | 1.53E-05 |
| 9 | 909407 | Glycoside Hydrolase Family 6 | cellulose | 11.68 | 1.38E-19 |
| 10 | 214246 | Dockerin x2, Glycoside Hydrolase Family 43 x4 | xylan | 11.65 | 7.58E-21 |

most downregulated

|  | ProteinID | Annotation | Substrate | Log2 Fold Change | p adjusted |
| --- | --- | --- | --- | --- | --- |
| 1 | 830849 | Glycoside Hydrolase Family 5 / Subf 4 | beta-glucan, xylan, beta-mannan | -12.27 | 2.20E-20 |
| 2 | 900407 | Dockerin, Glycoside Hydrolase Family 5 / Subf 4 | beta-glucan, xylan, beta-mannan | -12.25 | 4.30E-20 |
| 3 | 781227 | Dockerin x2, Glycoside Hydrolase Family 5 / Subf 4 | beta-glucan, xylan, beta-mannan | -9.76 | 1.11E-38 |
| 4 | 900404 | Dockerin, Glycoside Hydrolase Family 5 / Subf 4 | beta-glucan, xylan, beta-mannan | -9.43 | 7.61E-36 |
| 5 | 900393 | Dockerin, Glycoside Hydrolase Family 5 / Subf 4 | beta-glucan, xylan, beta-mannan | -8.98 | 3.33E-83 |
| 6 | 599515 | Carbohydrate-Binding Module Family 10 x3, Dockerin x2, Glycoside Hydrolase Family 5 / Subf 5 x2 | beta-glucan | -8.20 | 9.52E-97 |
| 7 | 777322 | Glycoside Hydrolase Family 5 / Subf 7 | - | -7.96 | 3.76E-47 |
| 8 | 777321 | Glycoside Hydrolase Family 5 / Subf 7 | - | -7.85 | 4.06E-62 |
| 9 | 583142 | Glycoside Hydrolase Family 5 / Subf 7 | - | -7.52 | 5.35E-08 |
| 10 | 606303 | Dockerin | - | -7.50 | 9.67E-29 |

**Reed canary grass vs. Filter paper**

most upregulated

|  | ProteinID | Annotation | Substrate | Log2 Fold Change | p adjusted |
| --- | --- | --- | --- | --- | --- |
| 1 | 788622 | Glycoside Hydrolase Family 3 | beta-glucan, xylan | 10.70 | 9.66E-27 |
| 2 | 820094 | Glycoside Hydrolase Family 11 | xylan | 10.47 | 9.24E-22 |
| 3 | 821869 | Glycoside Hydrolase Family 11 | xylan | 10.21 | 1.07E-18 |
| 4 | 885925 | Carbohydrate-Binding Module Family 6, Glycoside Hydrolase Family 43 | xylan,beta-glucan, cellulose | 10.20 | 2.61E-20 |
| 5 | 817649 | Glycoside Hydrolase Family 3 | beta-glucan, xylan | 10.20 | 3.03E-04 |
| 6 | 821051 | Carbohydrate-Binding Module Family 6, Glycoside Hydrolase Family 43 | xylan, beta-glucan, cellulose | 10.15 | 9.29E-21 |
| 7 | 909541 | Carbohydrate-Binding Module Family 1, Carbohydrate Esterase Family 3 | xylan | 10.13 | 4.55E-23 |
| 8 | 820093 | Glycoside Hydrolase Family 11 | xylan | 10.09 | 4.57E-06 |
| 9 | 891947 | Glycoside Hydrolase Family 30 / Subf 3 | beta-glucan | 9.85 | 3.50E-18 |
| 10 | 885928 | Carbohydrate-Binding Module Family 6, Glycoside Hydrolase Family 43 | beta-glucan, xylan, cellulose | 9.80 | 3.77E-24 |

most downregulated

|  | ProteinID | Annotation | Substrate | Log2 Fold Change | p adjusted |
| --- | --- | --- | --- | --- | --- |
| 1 | 812635 | Dockerin x2, Glycoside Hydrolase Family 5 / Subf 4 x2 | beta-glucan, xylan, beta-mannan | -8.05 | 2.43E-08 |
| 2 | 900407 | Dockerin, Glycoside Hydrolase Family 5 / Subf 4 | beta-glucan, xylan, beta-mannan | -7.65 | 3.88E-08 |
| 3 | 629388 | Carbohydrate-Binding Module Family 10, Glycoside Hydrolase Family 45 | beta-glucan | -7.60 | 1.73E-08 |
| 4 | 900768 | Glycoside Hydrolase Family 48 | cellulose, chitin | -6.80 | 4.03E-52 |
| 5 | 781227 | Dockerin x2, Glycoside Hydrolase Family 5 / Subf 4 | beta-glucan, xylan, beta-mannan | -6.71 | 2.65E-18 |
| 6 | 599515 | Carbohydrate-Binding Module Family 10 x3, Dockerin x2, Glycoside Hydrolase Family 5 / Subf 5 x2 | beta-glucan | -6.43 | 3.01E-59 |
| 7 | 911147 | Glycoside Hydrolase Family 6 | cellulose | -6.32 | 3.83E-07 |
| 8 | 900393 | Dockerin, Glycoside Hydrolase Family 5 / Subf 4 | beta-glucan, xylan, beta-mannan | -5.78 | 2.20E-34 |
| 9 | 626032 | Dockerin x2, Glycoside Hydrolase Family 48 | cellulose, chitin | -5.40 | 9.52E-52 |
| 10 | 606303 | Dockerin | - | -5.25 | 3.98E-14 |

**Filter paper vs. Glucose**

most upregulated

|  | ProteinID | Annotation | Substrate | Log2 Fold Change | p adjusted |
| --- | --- | --- | --- | --- | --- |
| 1 | 631948 | Glycoside Hydrolase Family 11 | xylan | 7.66 | 1.07E-11 |
| 2 | 909407 | Glycoside Hydrolase Family 6 | cellulose | 7.50 | 3.87E-09 |
| 3 | 625228 | Glycoside Hydrolase Family 18 | chitin, peptidoglycan | 7.29 | 6.94E-26 |
| 4 | 641176 | Carbohydrate-Binding Module Family 18 x3, Glycoside Hydrolase Family 18 | chitin, peptidoglycan | 7.17 | 1.40E-31 |
| 5 | 900768 | Glycoside Hydrolase Family 48 | cellulose, chitin | 7.05 | 1.10E-64 |
| 6 | 633701 | Carbohydrate-Binding Module Family 1, Glycoside Hydrolase Family 11 | xylan | 6.85 | 1.01E-07 |
| 7 | 884701 | Glycoside Hydrolase Family 6 | cellulose | 6.51 | 1.42E-62 |
| 8 | 575826 | Carbohydrate-Binding Module Family 18 x3, Glycoside Hydrolase Family 18 | chitin, peptidoglycan | 6.46 | 4.21E-60 |
| 9 | 889956 | Dockerin | - | 6.45 | 1.89E-07 |
| 10 | 780556 | Dockerin x2, Glycoside Hydrolase Family 48 | cellulose, chitin | 6.23 | 2.40E-11 |

most downregulated

|  | ProteinID | Annotation | Substrate | Log2 Fold Change | p adjusted |
| --- | --- | --- | --- | --- | --- |
| 1 | 777321 | Glycoside Hydrolase Family 5 / Subf 7 | - | -4.95 | 2.78E-64 |
| 2 | 777322 | Glycoside Hydrolase Family 5 / Subf 7 | - | -4.67 | 1.96E-37 |
| 3 | 618105 | Carbohydrate-Binding Module Family 10 x2, Glycoside Hydrolase Family 5 / Subf 8 | beta-mannan | -4.31 | 5.64E-04 |
| 4 | 830849 | Glycoside Hydrolase Family 5 / Subf 4 | beta-glucan, xylan, beta-mannan | -3.61 | 1.48E-11 |
| 5 | 648997 | Carbohydrate Esterase Family 6, Dockerin x2 | xylan | -3.53 | 6.51E-13 |
| 6 | 900407 | Dockerin, Glycoside Hydrolase Family 5 / Subf 4 | beta-glucan, xylan, beta-mannan | -3.41 | 3.31E-10 |
| 7 | 269745 | Dockerin x2, Glycoside Hydrolase Family 6 | cellulose | -3.20 | 9.93E-06 |
| 8 | 900404 | Dockerin, Glycoside Hydrolase Family 5 / Subf 4 | beta-glucan, xylan, beta-mannan | -3.14 | 1.78E-07 |
| 9 | 789055 | Dockerin x2, Glycoside Hydrolase Family 5 / Subf 4 | - | -2.71 | 5.22E-05 |
| 10 | 897373 | Glycoside Hydrolase Family 5 / Subf 4 | beta-glucan, xylan, beta-mannan | -2.70 | 6.49E-07 |

**Filter paper vs. Cellobiose**

most upregulated

|  | ProteinID | Annotation | Substrate | Log2 Fold Change | p adjusted |
| --- | --- | --- | --- | --- | --- |
| 1 | 909407 | Glycoside Hydrolase Family 6 | cellulose | 8.61 | 5.06E-11 |
| 2 | 777905 | Carbohydrate-Binding Module Family 10 x2, Glycoside Hydrolase Family 45 | beta-glucan | 7.73 | 6.68E-09 |
| 3 | 884701 | Glycoside Hydrolase Family 6 | cellulose | 7.67 | 7.04E-51 |
| 4 | 625228 | Glycoside Hydrolase Family 18 | chitin, peptidoglycan | 7.51 | 1.78E-23 |
| 5 | 631948 | Glycoside Hydrolase Family 11 | xylan | 7.33 | 1.47E-10 |
| 6 | 781642 | Carbohydrate Esterase Family 1, Dockerin x2, Glycoside Hydrolase Family 11 | xylan | 7.27 | 1.15E-34 |
| 7 | 817812 | Carbohydrate-Binding Module Family 18, Glycoside Hydrolase Family 114 | - | 7.06 | 5.45E-07 |
| 8 | 780556 | Dockerin x2, Glycoside Hydrolase Family 48 | cellulose, chitin | 6.91 | 4.01E-13 |
| 9 | 545661 | Carbohydrate Esterase Family 4 | - | 6.83 | 2.52E-09 |
| 10 | 640448 | Carbohydrate-Binding Module Family 13, Dockerin x2 | xylan | 6.82 | 1.26E-09 |

most downregulated

|  | ProteinID | Annotation | Substrate | Log2 Fold Change | p adjusted |
| --- | --- | --- | --- | --- | --- |
| 1 | 830849 | Glycoside Hydrolase Family 5 / Subf 4 | beta-glucan, xylan, beta-mannan | -6.08 | 4.35E-32 |
| 2 | 583142 | Glycoside Hydrolase Family 5 / Subf 7 | - | -5.48 | 7.79E-07 |
| 3 | 777322 | Glycoside Hydrolase Family 5 / Subf 7 | - | -5.30 | 3.02E-48 |
| 4 | 52035 | Carbohydrate-Binding Module Family 1, Carbohydrate Esterase Family 12 | pectin | -5.17 | 4.99E-07 |
| 5 | 777321 | Glycoside Hydrolase Family 5 / Subf 7 | - | -5.16 | 1.10E-69 |
| 6 | 900407 | Dockerin, Glycoside Hydrolase Family 5 / Subf 4 | beta-glucan, xylan, beta-mannan | -4.60 | 2.76E-18 |
| 7 | 900404 | Dockerin, Glycoside Hydrolase Family 5 / Subf 4 | beta-glucan, xylan, beta-mannan | -4.36 | 7.35E-14 |
| 8 | 894808 | Glycoside Hydrolase Family 115 | xylan | -3.67 | 7.55E-10 |
| 9 | 900393 | Dockerin, Glycoside Hydrolase Family 5 / Subf 4 | beta-glucan, xylan, beta-mannan | -3.20 | 8.54E-22 |
| 10 | 781227 | Dockerin x2, Glycoside Hydrolase Family 5 / Subf 4 | beta-glucan, xylan, beta-mannan | -3.06 | 2.50E-19 |

**Cellobiose vs. Glucose**

most upregulated

|  | ProteinID | Annotation | Substrate | Log2 Fold Change | p adjusted |
| --- | --- | --- | --- | --- | --- |
| 1 | 911147 | Glycoside Hydrolase Family 6 | cellulose, cellulose | 5.40 | 5.07E-14 |
| 2 | 329678 | Dockerin, Glycoside Hydrolase Family 10 | xylan | 3.10 | 7.30E-05 |
| 3 | 830849 | Glycoside Hydrolase Family 5 / Subf 4 | beta-glucan, xylan, beta-glucan, beta-mannan | 2.47 | 1.43E-05 |
| 4 | 900768 | Glycoside Hydrolase Family 48 | cellulose, cellulose, chitin | 2.39 | 4.54E-07 |
| 5 | 820551 | Glycoside Hydrolase Family 48 | cellulose, cellulose, chitin | 2.22 | 9.95E-09 |
| 6 | 820550 | Glycoside Hydrolase Family 48 | cellulose, cellulose, chitin | 2.16 | 8.35E-09 |
| 7 | 894808 | Glycoside Hydrolase Family 115 | xylan | 2.01 | 2.59E-03 |
| 8 | 268491 | Dockerin x2, Glycoside Hydrolase Family 6 | cellulose, cellulose | 2.00 | 5.32E-09 |
| 9 | 911142 | Glycoside Hydrolase Family 6 | cellulose, cellulose | 1.70 | 3.29E-05 |
| 10 | 594700 | Carbohydrate Esterase Family 4 | chitin | 1.48 | 6.02E-05 |

most downregulated

|  | ProteinID | Annotation | Substrate | Log2 Fold Change | p adjusted |
| --- | --- | --- | --- | --- | --- |
| 1 | 777515 | Carbohydrate-Binding Module Family 1, Glycoside Hydrolase Family 6 | cellulose | -4.93 | 9.23E-17 |
| 2 | 781642 | Carbohydrate Esterase Family 1, Dockerin x2, Glycoside Hydrolase Family 11 | xylan | -4.80 | 3.87E-14 |
| 3 | 146718 | Dockerin x2, Glycoside Hydrolase Family 6 | cellulose | -4.38 | 7.16E-16 |
| 4 | 906540 | Glycoside Hydrolase Family 1 | beta-glucan | -4.31 | 5.91E-11 |
| 5 | 576612 | Polysaccharide Lyase Family 3 / Subf 2 | pectin | -3.92 | 2.06E-06 |
| 6 | 788477 | Carbohydrate-Binding Module Family 1, Glycoside Hydrolase Family 11 | xylan | -3.79 | 9.45E-06 |
| 7 | 153450 | Dockerin x2, Glycoside Hydrolase Family 6 | cellulose | -3.63 | 3.99E-24 |
| 8 | 915113 | Carbohydrate-Binding Module Family 26, Glycoside Hydrolase Family 31 | starch, xyloglucan | -3.62 | 9.43E-07 |
| 9 | 891591 | Glycoside Hydrolase Family 43 | xylan | -3.54 | 1.34E-31 |
| 10 | 888447 | Glycoside Hydrolase Family 43 | xylan | -3.39 | 4.61E-07 |

**Table S14**. Pairwise comparisons show differentially upregulated and downregulated genes encoding CAZymes for different carbon substrates.

| Pairwise substrate comparison | Total differentially expressed CAZymes* | Upregulated | Downregulated |
| --- | --- | --- | --- |
| reed canary grass vs. glucose | 1610 | 1307 | 303 |
| reed canary grass vs. cellobiose | 1696 | 1372 | 324 |
| reed canary grass vs. filter paper | 1496 | 1190 | 306 |
| filter paper vs. glucose | 731 | 507 | 224 |
| filter paper vs. cellobiose | 907 | 646 | 261 |
| cellobiose vs. glucose | 347 | 98 | 249 |

*Differentially expressed CAZymes include only genes predicted to encode CAZymes with > 2 average TPM for at least one of the conditions in comparison and padj < 0.05.

**Table S15**. AlphaFold predicted structures of proteins 782539, 640448, 650522, 774834, and 783149. Structural homology searching with Foldseek predicts that these proteins are feruloyl esterases.

| **Protein ID** | **Filter paper - glucose log_2_(FC)** | **Filter paper - glucose P_adj_** | **Reed canary grass -glucose log_2_(FC)** | **Reed canary grass - glucose P_adj_** |
| --- | --- | --- | --- | --- |
| **782539** | **-0.118** | **0.942** | **7.75** | **3.69 x 10^-36^** |
| **640448** | **5.21** | **1.60 x 10^-14^** | **7.52** | **1.19 x 10^-29^** |
| **650522** | **-0.483** | **0.653** | **4.6** | **2.84 x 10^-11^** |
| **774834** | **1.35** | **0.0165** | **3.43** | **7.04 x 10^-11^** |
| **783149** | **0.0822** | **0.914** | **2.11** | **3.75 x 10^-7^** |

**Table S16.** Enumerated CAZyme domains in six additional anaerobic fungi. Annotations were obtained from MycoCosm (Grigoriev et al. 2014) and the Carbohydrate Active Enzymes database (<http://www.cazy.org/>) (Drula et al. 2022).

| ***Anaeromyces robustus*** (Haitjema et al. 2017) | | | | | |
| --- | --- | --- | --- | --- | --- |
| CAZyme class | Protein count | Proteins containing dockerin domains | Fraction containing dockerin domains | Proteins with annotated CBM | Fraction with CBM |
| Carbohydrate esterase (CE) | 93 | 12 | 0.13 | 32 | 0.34 |
| Glycoside hydrolase (GH) | 252 | 85 | 0.34 | 66 | 0.26 |
| Glycosyltransferase (GT) | 118 | 0 | 0 | 0 | 0 |
| Polysaccharide lyase (PL) | 10 | 2 | 0.20 | 5 | 0.5 |
|  |  |  |  |  |  |
| ***Caecomyces churrovis*** (Brown et al. 2021) | | | | | |
| CAZyme class | Protein count | Proteins containing dockerin domains | Fraction containing dockerin domains | Proteins with annotated CBM | Fraction with CBM |
| Carbohydrate esterase (CE) | 90 | 18 | 0.20 | 37 | 0.41 |
| Glycoside hydrolase (GH) | 282 | 100 | 0.35 | 53 | 0.19 |
| Glycosyltransferase (GT) | 125 | 0 | 0 | 0 | 0 |
| Polysaccharide lyase (PL) | 7 | 3 | 0.43 | 1 | 0.14 |
|  |  |  |  |  |  |
| ***Neocallimastix californiae*** (Haitjema et al. 2017) | | | | | |
| CAZyme class | Protein count | Proteins containing dockerin domains | Fraction containing dockerin domains | Proteins with annotated CBM | Fraction with CBM |
| Carbohydrate esterase (CE) | 156 | 16 | 0.10 | 58 | 0.37 |
| Glycoside hydrolase (GH) | 517 | 168 | 0.32 | 123 | 0.24 |
| Glycosyltransferase (GT) | 177 | 0 | 0.00 | 0 | 0.00 |
| Polysaccharide lyase (PL) | 81 | 1 | 0.01 | 32 | 0.40 |
|  |  |  |  |  |  |
| ***Neocallimastix lanati*** (Wilken et al. 2021) | | | | | |
| CAZyme class | Protein count | Proteins containing dockerin domains | Fraction containing dockerin domains | Proteins with annotated CBM | Fraction with CBM |
| Carbohydrate esterase (CE) | 165 | 24 | 0.15 | 66.00 | 0.40 |
| Glycoside hydrolase (GH) | 678 | 271 | 0.40 | 158.00 | 0.23 |
| Glycosyltransferase (GT) | 197 | 0.0 | 0.0 | 0.0 | 0.0 |
| Polysaccharide lyase (PL) | 83 | 3 | 0.04 | 34.00 | 0.41 |
|  |  |  |  |  |  |
| ***Piromyces finnis*** (Haitjema et al. 2017) | | | | | |
| CAZyme class | Protein count | Proteins containing dockerin domains | Fraction containing dockerin domains | Proteins with annotated CBM | Fraction with CBM |
| Carbohydrate esterase (CE) | 82 | 11 | 0.13 | 41 | 0.50 |
| Glycoside hydrolase (GH) | 262 | 104 | 0.40 | 61 | 0.23 |
| Glycosyltransferase (GT) | 100 | 0 | 0 | 0 | 0 |
| Polysaccharide lyase (PL) | 15 | 2 | 0.13 | 8 | 0.53 |
|  |  |  |  |  |  |
| ***Piromyces sp. UH3-1*** |  |  |  |  |  |
| CAZyme class | Protein count | Proteins containing dockerin domains | Fraction containing dockerin domains | Proteins with annotated CBM | Fraction with CBM |
| Carbohydrate esterase (CE) | 186 | 47 | 0.25 | 65 | 0.35 |
| Glycoside hydrolase (GH) | 477 | 200 | 0.42 | 109 | 0.23 |
| Glycosyltransferase (GT) | 134 | 0 | 0.00 | 0 | 0.00 |
| Polysaccharide lyase (PL) | 37 | 4 | 0.11 | 23 | 0.62 |

**References**

Bertella S, Luterbacher JS. 2020. Lignin functionalization for the production of novel materials. Trends in Chemistry. 2(5):440–453. doi:10.1016/j.trechm.2020.03.001.

Brown JL, Swift CL, Mondo SJ, Seppala S, Salamov A, Singan V, Henrissat B, Drula E, Henske JK, Lee S, et al. 2021. Co‑cultivation of the anaerobic fungus *Caecomyces churrovis* with *Methanobacterium bryantii* enhances transcription of carbohydrate binding modules, dockerins, and pyruvate formate lyases on specific substrates. Biotechnol Biofuels. 14(1):234. doi:10.1186/s13068-021-02083-w.

Cantarel BL, Coutinho PM, Rancurel C, Bernard T, Lombard V, Henrissat B. 2009. The Carbohydrate-Active EnZymes database (CAZy): an expert resource for Glycogenomics. Nucleic Acids Research. 37(Database):D233–D238. doi:10.1093/nar/gkn663.

Drula E, Garron M-L, Dogan S, Lombard V, Henrissat B, Terrapon N. 2022. The carbohydrate-active enzyme database: functions and literature. Nucleic Acids Research. 50(D1):D571–D577. doi:10.1093/nar/gkab1045.

Grigoriev IV, Nikitin R, Haridas S, Kuo A, Ohm R, Otillar R, Riley R, Salamov A, Zhao X, Korzeniewski F, et al. 2014. MycoCosm portal: gearing up for 1000 fungal genomes. Nucl Acids Res. 42(D1):D699–D704. doi:10.1093/nar/gkt1183.

Haitjema CH, Gilmore SP, Henske JK, Solomon KV, de Groot R, Kuo A, Mondo SJ, Salamov AA, LaButti K, Zhao Z, et al. 2017. A parts list for fungal cellulosomes revealed by comparative genomics. Nat Microbiol. 2:17087. doi:10.1038/nmicrobiol.2017.87.

Wilken StE, Monk JM, Leggieri PA, Lawson CE, Lankiewicz TS, Seppälä S, Daum CG, Jenkins J, Lipzen AM, Mondo SJ, et al. 2021. Experimentally validated reconstruction and analysis of a genome-scale metabolic model of an anaerobic Neocallimastigomycota fungus. mSystems. 6(1):e00002-21. doi:10.1128/mSystems.00002-21.
